# Supplementary material for: Synaptically-targeted long non-coding RNA SLAMR promotes structural plasticity by increasing translation and CaMKII activity
Source: Nat Commun. 2024 Mar 27;15:2694. doi: 10.1038/s41467-024-46972-8 (PMC10973417; doi:10.1038/s41467-024-46972-8)
Supplement: Supplementary file 1 — Supplementary Information [file 41467_2024_46972_MOESM1_ESM.pdf]

SUPPLEMENTARY INFORMATION

Synaptic-targeted long non-coding RNA SLAMR promotes structural plasticity by increasing translation and CaMKII activity

Isabel Espadas[1#], Jenna L. Wingfield[1#], Yoshihisa Nakahata[2], Kaushik Chanda[1], Eddie Grinman[1], Ilika Ghosh[2], Karl E. Bauer[3], Bindu Raveendra[1], Michael A. Kiebler[3], Ryohei Yasuda[2], Vidhya Rangaraju[2], Sathyanarayanan Puthanveettil[1*]

Supplementary Methods

8 Supplementary Figures (S1-8)

12 Supplementary Tables (S1-12)

9 Supplementary Movies (S1-9)

SUPPLEMENTARY METHODS

RNAseq

Tissue preparation, LCM, and RNA isolation

One hour after finishing the training in CFC or MWM, n= 3-4 mice x group x condition were sacrificed by a fast decapitation and the brains were removed, briefly washed in D-PBS, placed in cryomolds with OCT, and frozen in dry ice, then stored at -80°C (Kadakkuzha et al., 2015). Fresh frozen brains were coronal sectioned at 14μm on a Leica 3050s cryostat at -20°C after 1h of acclimation to this temperature. Dorsal hippocampal sections were mounted on PEN membrane slides (Leica), ˜12 sections per slide with 3 slides per brain from a total of 6 brains. Staining and preparation of sections for the Laser Capture Microdissection (LCM) procedure was done using an LCM staining kit (Life Technologies) with Cresyl Violet following the manufacturer's recommendations. Following staining and dehydration, slides were kept at room temperature for 15 min before LCM. The microdissection of the CA1 and CA3 hippocampal regions were done using the Leica LCM microscope (Leica LMD7000) where the laser power was set at 60 mW. Tissue was collected in RNAase-free microtubes where 50μl of pre-chilled trizol was added just after finishing the micro sectioning. RNA was extracted using the trizol-Chloroform method and stored at -80°C before use.

Next-generation sequencing and data analysis

RNA collected from the previous step was used for RNAseq of CFC experiments using two animals x condition x group for a total of 6 mice. RNAseq analysis was carried out following the Wertheim UF Scripps Institute Genomics core protocol described in previous works from this laboratory (Kaddakuzha et al., 2015; Raveendra et al., 2018; Grinman et al., 2021). Analysis of the RNAseq results was performed using Tophat2 (v.2.0.9) and the Cufflinks suite (v.2.1.1). Trimming of the data was performed using the FastX-Toolkit (v.0.013) (Toolkit by Hannon Lab) (Raveendra et al., 2018). The alignment data were processed and quantified using HTSeq. The raw read counts generated in HTSeq were used to identify differentially expressed genes by using the Bioconductor DEseq2 for R. DEseq2 uses the Wald Test to generate *p*-values and the Benjamini and Hochberg

method to generate *p*-adjusted values. Comparisons were performed between samples of the three different CFC groups: Context+Shock, Context alone, and Immediate shock mice. Transcripts were extracted from the Ensembl annotation file (NCBIM37).

Interactome analysis

We used Metascape for pathway analysis (https://www.metascape.org) (Zhou et al., 2019) to analyze the list of significantly different genes in common between Context vs. Context+Shock & Shock vs. Context+Shock conditions, all of them based on a pVal<0.05 and create an interactome analysis. A network with the enriched ontology clusters was visualized with Cytoscape. Each term was represented by a circle node proportional to the number of genes included in that term identified by a specific color.

Quantitative real-time PCR (qRT-PCR)

The RNA from the LCM samples was reverse transcribed to cDNA using the same method previously reported from this laboratory (Kadakkuzha et al., 2013; Raveendra et al., 2018). 1µg of RNA was used with Quanta cDNA SuperMix (Quanta Biosciences, Gaithersburg, MD) according to the manufacturer's instructions, and the expression of transcripts were quantified by qRT-PCR using SYBR Green PCR master mix (Applied Biosystems Carlsbad, CA) for detection in ABI 7900 cycler (Applied Biosystems Carlsbad, CA). Quantification of each transcript was normalized to the mouse 18S reference gene following the $2^{-\Delta\Delta Ct}$ method (Livak and Schmittgen, 2001; Kadakkuzha et al., 2013). One-way ANOVA and Student-Newman test were used to select genes with statistically significant expression levels.

Localization studies of SLAMR

FISH

Mice were sacrificed by cervical dislocation and the brain was removed from the skull, briefly washed in pre-cold D-PBS, embedded in OCT, and frozen in dry ice. Then, the fresh frozen brains were cryosectioned at 16µm on a Leica cryostat 3050 (Leica Systems). A DIG-labeled ribo probe complementary to SLAMR lncRNA was prepared by

in vitro transcription of cDNA templates by using SP6/T& RNA polymerases (DIG RNA labeling Kit, Sigma). Sense and Antisense strands of 250nt were prepared by PCR using mouse hippocampus cDNA as a template and transcript-specific PCR primers and ligated to pCRII-TOPO Vector with dual promoters SP6 (for antisense strand) and T7 (sense strand).

After the sectioning the tissue was dried and acclimated at RT for 2 hours, washed with D-PBS fixed with a 4% PFA solution for 10 min at RT, washed again with D-PBS (3 times, 3 min each), washed with 0.2% glycine in D-PBS (5 min), D-PBS washes (2 x 5 min), acetylated in TEA solution for 10 min, pre-hybridized at 68°C for 1 hr and hybridize with the probes overnight (approx. 16 hrs). After hybridization, tissues were incubated with primary antibodies (1:1000 anti-DIG) for 1hr at room temperature and washed (3 x 10min) with D-PBS before the signal was visualized using the TSA Plus Fluorescein Systems from PerkinElmer (TSA plus Cyanine detection kit, Akoya) for DIG detection. Images were acquired by using Zeiss LSM 880 with Airyscan confocal microscope system.

Fractionation

Fractionation experiments were carried out using C57BL6 adult male mice hippocampus lysates. The SurePrep™ Nuclear or Cytoplasmic RNA purification kit (Fisher Scientific) was used following the manufacturer's manual. The RNA isolated was reverse transcribed and RT-qPCR was performed following the procedures described before in these methods. Actin mRNA that is enriched in the cytoplasm was used as a control for the fractionation and a normalization element. Following the same procedure previously published by our lab. CT values of each lncRNA were normalized to Actin, resulting in an ΔCT value. Relative differences between lncRNAs and Actin were determined by subtracting the ΔCTs from the nucleus fraction from the ΔCTs of cytoplasm fraction for each sample, resulting in ΔΔCT values (Kadakkuzha et al., 2015).

Primary Hippocampal Cell Cultures

Neuronal cultures from hippocampi were obtained from the brains of E17/18 mixed-sex CD1 mice embryos or from the brains of P0 Sprague Dawley rat pups following the same procedure described before from our laboratory (Raveendra et al., 2018). Embryonic or

P0brains were removed from the skulls and membranes were cleaned with dissection media. Then, brains were dissociated using papain (29.5 U/mg protein, Worthington). The cells obtained were plated in poly-D-lysine (PDL) treated plates with a $5x10^5$ density for experiments that required RNA isolation or a density of 1.5 $x10^5$ in glass coverslips for imaging experiments. Both types of cultures were maintained in Neurobasal medium (Invitrogen), penicillin/streptomycin, and 2% B27 (Invitrogen) at 37°C in 5% CO2.

Loss of function analysis using Gapmers

Three different antisense LNA Gapmers against 2610035D17Rik and a Negative Control Gapmer were designed by EXIQON (QIAGEN) and synthesized for *in vitro* (Gapmer_1: GGACAGGTCAATGGCG; Gapmer_2: TGATGTGAGTTTCTAC, Gapmer_3: ACAAACGAGATAGTAG) and/or *in vivo* experiments. (Gapmer_1: GGACAGGTCAATGGCG *in vivo* purified, named as: SLAMR_Gapmer in this work)

In vitro efficiency: Gapmer's efficiency were first tested in primary hippocampal cell cultures. Gapmers were transfected to primary hippocampal neurons (7-14 days in vitro (DIV)) using Lipofectamine RNAiMAX according to manufacturer's guidelines. For the transfection 6 pmol of Gapmer was mixed with 1µl of Lipofectamine RNAi Max in 100µl of Neurobasal medium and incubated for 15 min. The complexes of RNAi and Lipofectamine RNAiMAX were added to the cells in incubation at 37°C. 48 hrs and 72 hrs after the transfection with each Gapmer, cells were washed with PBS and collected into trizol to proceed with the RNA extraction using the trizol-chloroform protocol. Reverse transcription and RT-qPCR was performed following the same method described before in this work.

In vivo efficiency: We decided to determine the efficiency *in vivo* of Gapmer_1 (SLAMR_Gapmer for behavior) before the behavioral performance. For this, 8 weeks old C57BL6 mice were bilaterally infused in the dorsal-Hippocampus CA1 area with a single infusion of *in vivo* ready SLAMR_Gapmer or a Negative control using the transfection reagent JetSI (Polyplus-transfection S.A, Illkirch, France) (25pmols, 0.5µl per side at 0.1µl/min delivery rate). Using the following coordinates: AP=-2, DV=-1.5, L=±1.5. 72hrs after the infusion mice were sacrificed and the CA1 dorsal-hippocampal area was dissected and fast-freezed in dry ice. The tissue collected was processed using the kit

RNA Aqueous 4PCR kit (Ambion, Invitrogen). 200µl of lysis buffer from this kit was used to grind the tissue and then we proceeded according to the manufacturer's instructions. The RNA obtained was reverse transcribed and the levels of SLAMR were measured by RT-qPCR following the same method described before.

Constructs & Transfections

For lipofectamine transfection, about 1.50 x 10^5 cells/well primary mouse hippocampal neurons were plated on PDL-coated CellVis glass-bottom 24-well plates (P24-1.5H-N). For pLL3.7-GFP scramble control (Addgene #11795), SLAMR-shRNA (#CS-GS1731L, Genecopeia), TET-SLAMR (#CS-SH1343L, Genecopoeia), and OE-SLAMR (#CS-GS1731L-Lv235-01) arborization analysis were transfected 68-74 hrs before imaging (DIV14) using Lipofectamine 2000 (Thermo) following the manufacturer's instruction. Before adding the DNA-lipofectamine mixture, half of the conditioned culture medium was removed and saved for later. Four hours after incubation with DNA-lipofectamine mixture, the medium was removed, and immediately added 500µl of 1:1 of conditioned/fresh medium. Right before imaging, all culture medium was replaced by 500 µl of Hibernate E low fluorescence buffer (BrainBits) to maintain the ambient pH environment.

For magnetofection, about 6x10^4 cells/dish primary Rat hippocampal neurons were plated on the center of a PDL-coated coverglass region of MatTek dishes (P35G-1.5-14-C). For SLAMR-MS2: MCP (Plasmids: MS2-SLAMR #CS-CC1532L, Genecopoeia; tdMCP #98916 addgene; RFP-MCP #64541 addgene; MS2-SLAMRΔ92-289, CS-CC1532L-02 Genecopoeia; MS2-SLAMR-Δ898-1130 #CS-CC1532L-03, Genecopoeia) imaging, hippocampal neurons were transfected 12-16 hrs (DIV14-22) prior to imaging using Combimag (OZ biosciences) and Lipofectamine 2000 (Invitrogen) according to the manufacturer's instructions. Briefly, 1.2 µg DNA was incubated with 1 µl lipofectamine in 100 µl transfection medium (Neurobasal media supplemented with 2 mM Glutamax without B27 or antibiotics) for 5 min, then mixed with 1 µl Combimag for 10 min. The DNA-lipofectamine-Combimag mixture was further diluted in 250 µl of transfection medium. Then, the conditioned medium from cultured neurons was harvested and the neurons were immediately rinsed twice in 1 ml warm transfection medium. The transfection medium used for rinsing was removed and 150 µl of DNA-lipofectamine-Combimag

mixture was added to the neurons. Neurons were placed on a magnetic plate for 25 min inside a 37 °C and 5% CO2 incubator. After 25 min, neurons were rinsed once in 1 ml warm transfection medium and replaced with the previously harvested warm, conditioned medium. Right before imaging, all culture medium was replaced by 1ml of Hibernate E low fluorescence buffer (BrainBits) to maintain the ambient pH environment.

Lentiviral production

pLL3.7-GFP-ShLenti against SLAMR (SH1343L) and pLL3.7-GFP scramble control (Addgene #11795). Each plasmid was co-transfected with REV, MDL packing vectors and vesicular stomatitis virus (VSVG) envelop vector from Addgene into HEK293T cells using TransIT transfection reagent (Mirus) for lentiviral particle production. The supernatant was collected at two different time points 36 and 72hrs after transfection. The lentiviral particles were concentrated and pelleted through a sucrose cushion and re-suspended in sterile PBS and stored at -80°C. Viral titer was determined by measuring GFP fluorescence after infection of HEK293T cells with serial dilutions of the virus, using flow-cytometry (BD biosciences C6 Accuri and InterlliCyt sampler powered by FlowCyt software). Our lab-made SLAMR-OE lentivirus had a titer of 3.9×10^9 IFU/ml. Vigene synthesized NC-GFP and SLAMR-KD lentiviruses at titers of 1.07×10^9 IFU/ml and 9.75×10^8 IFU/mL respectively.

SLAMR-MS2: MCP transport timelapse video microscopy

Live imaging of rat hippocampal neuronal cultures was carried out using a state-of-the-art inverted Spinning Disk Confocal microscope (3i imaging systems, Yokogawa CSU-W1 confocal scanner unit) with 4 laser lines (405 nm 20 mW; 488 nm 50 mW, 561 nm 75 mW and 638 nm 75 mW) connected to a charged coupled device confocal camera (Andor iXon Life 888). The temperature of the sample stage was maintained at 37 °C by an Okolab Boldline Stage Top Incubation system. The image acquisition was controlled by SlideBook6 software. For MS2-SLAMR: MCP-tdGFP transport and PSD95-mcherry imaging, neurons were co-transfected and imaged within 12-20hrs post-transfection in Tyrodes buffer (in mM: 119 NaCl, 2.5 KCl, 2 $CaCl_2$, 2 $MgCl_2$, 25 HEPES, 30 D-Glucose; pH 7.4). Timelapse confocal imaging of dendrites at least 10 μm away from the soma was

performed with a 63x oil objective (1.46 N.A.). For each neuron, a movie was first captured in the GFP channel at 600 ms exposure and at a rate of 1-1.03 frames/s for a total recording time of 300 s. Following this acquisition, a movie was captured in the mCherry channel at 100 ms exposure at a rate of 10 frames/s for a total recording time of 120 s.

Time-series image data of reporter mRNAs was analyzed by kymographs. Dendritic 100-μm segments at ≥20 μm from the cell body were selected. The KymoResliceWide plugin of ImageJ was used to generate kymographs and trace tracks. Velocities were calculated from the angle of the traced tracks where Velocity (μm/s) = TAN ($\pi/180 \times \theta$) × fps/(pixels/μm); fps = frames per second. Only movements greater than 1.5 μm were considered for analysis. Tracks were terminated when a particle stopped, changed direction, or left the region of interest (ROI). Average speed and displacement were obtained by calculating the mean. Anterograde and retrograde tracks were counted to calculate the percent of anterograde transport. The sum of anterograde and retrograde displacement lengths was used to calculate the percentage of total anterograde displacement. Dual-color kymographs were generated by overlaying identical regions of interest from two separate channels. Events in dual-color kymographs were manually selected, and distances were manually measured in ImageJ (line tool). For the MS2-SLAMR:MCP spine interaction studies, MS2-SLAMR, and dendritic spines were considered to have an interaction with each other if the MS2-SLAMR (green track) came within 1 μm of a PSD95 puncta (red track) based on the kymograph. Data were processed and subjected to statistical analyses in GraphPad Prism 9.

MS2-SLAMR two-photon glutamate uncaging experiments

Rat primary hippocampal neuronal cultures were transfected with MS2-SLAMR: MCP-tdGFP and RCaMP1.07 16-20hrs prior to imaging. RCaMP1.07 fluorescence was used to identify responsive spines for glutamate uncaging. Prior to single spine stimulation, the imaging media was replaced with a modified Tyrodes buffer (as above but with 4 mM $CaCl_2$ and no Mg^{2+}) containing 1 μM TTX (citrate salt, made in water) and 2 mM 4-Methoxy-7-nitroindolinyl-caged-L-glutamate (MNI caged glutamate; Tocris Bioscience, 100 mM stock made in the modified Tyrodes buffer). Glutamate uncaging was performed

using a multiphoton laser at 720 nm (MaiTai HP 1040S) and a pockel cell (Conoptics) for controlling the pulses. Simultaneous confocal imaging and two-photon glutamate uncaging were performed using the SlideBook6 software in association with the MaiTai 2x software for controlling the laser beam. Spines (primarily mushroom-shaped) positioned at least 50 µm away from the soma, on secondary or tertiary branches, were selected for these experiments. Baseline timelapse recordings of the GFP (600 ms exposure) and RCaMP1.07 (100 ms exposure) fluorescence were acquired for 1 min at a rate of 1.2 frames/s prior to glutamate uncaging. To test if a spine responds to uncaging, an uncaging spot (~ 1 x 1 pixel or ~ 1 μm^2) was placed near the spine head, and one to two stimulation pulses were given at a pixel duration of 10 ms and with a laser power of 1 mW. The spines showing specific localized calcium transients measured by an increase in RCaMP1.07 fluorescence were chosen for the experiment. An uncaging protocol of 30 pulses at 0.5 Hz with 10 ms pixel duration and 1 mW laser power was used. Immediately following uncaging, another 45 s of continuous timelapse frames were recorded at the same frame rate as above. For the analysis of change in RNA granules following stimulation: Within 60 s after stimulation, timelapse movies with 3D Z-stacks (11 slices, ~ 5 µm depth and 0.5 µm step size) were acquired in both the GFP (600 ms) and RCaMP1.07 (100 ms) channels. The same Z stack timelapse (11 slices, ~ 5 µm depth, and 0.5 µm step size) imaging was repeated every 1 min until 5 min after stimulation, then every 2 mins until 30 min, and continued every 5 min until 60 min after stimulation. For the analysis of SLAMR velocity and trajectory, a 2 min timelapse movie at ~1.1 fps was taken in the GFP channel (600 ms) before stimulation, then stimulation occurred as above while taking a timelapse movie in the RCaMP1.07 (100 ms) and GFP (100 ms) channel, followed immediately by a single frame in the RCaMP1.07 channel and a 5 min timelapse at ~1.1 fps was taken in the GFP channel (600ms). Then, every ten minutes, a 2 min timelapse at ~1.1 fps was taken in the GFP channel (600 ms) until 50 min.

Synaptic protein extraction

Mouse primary cultured hippocampal neurons on 6-well plates were processed for synaptoneurosome preparation after transfections. Neuronal culture medium was carefully removed, and after two rinses in ice-cold PBS, cells were lysed manually in Syn-

Per buffer (Syn-PERTM Synaptic Protein Extraction Reagent, ThermoScientific) supplemented with 1 protease inhibitor cocktail tablet and 100 µL of phosphatase inhibitor cocktail 1 and 2 each. Samples were centrifuged at 1200 xg for 10 min at 4°C, the pellet was discarded, and the supernatant was transferred to a new tube. Thirty microliters from the sample of the supernatant were saved as homogenate for analysis. The supernatant was centrifuged at 15,000 xg for 20 min at 4°C. The supernatant was removed from the synaptosome pellet and saved as the cytosolic fraction for analysis. The synaptosome pellet was suspended in 30 µL Syn-PER.

Morphology Assessments

After 72 hrs of transfection and 24hrs of incubation with 0.5ug of Doxycycline, mice hippocampal neurons using shRNA plasmid expressing NC-GFP, TET-SLAMR, and OE-SLAMR images of dendrites were collected at 36°C in the light microscopy facility at UF Scripps Biomedical Research, using a confocal microscope (FV1000; Olympus; Apo N 60X/1.49 Oil) in Hibernate-E (Brainbits). Z-stack images were acquired using Fluoview1000 (64 bit) software (Olympus) and converted into a maximum projection intensity image in FIJI (ImageJ, NIH). The dendritic arbor was quantified via the Sholl analysis plugin in FIJI. The center of the soma is considered as the midpoint and the origin of the concentric radii was set from that point to the longest axis of the soma. The parameters set for analysis start at a radius of 20 µm, and end at a radius of 110 µm, with a radius step size of 10 µm. The maximum value of sampled intersections reflecting the highest number of processes/branches in the arbor was calculated and the number of intersections was plotted against the distance from the soma center in µm. Data was analyzed using Two-way ANOVA.

Spine morphology was analyzed using MATLAB software developed in the light microscopy facility at the Max Planck Florida Institute. By using a geometric approach, this software automatically detects and quantifies the structure of dendritic spines from the selected secondary branch (100 µm length) in the Z-stack confocal image. The software assigns the detected spines to one of the three morphological categories (thin, stubby, or mushroom) based on the difference in structural components of the spines i.e., head, neck, and shaft.

Two-photon fluorescence microscopy and two-photon glutamate uncaging for analysis of spine morphology

MNI-caged glutamate uncaging and timelapse structural imaging of spines were performed using a custom-built two-photon (2p) laser microscope as previously described (Colgan et al., 2018). 2p-imaging and uncaging was performed using two Ti-sapphire lasers (Coherent, Cameleon) at wavelengths of 920 nm (1.45–1.55 mW under the objective) for imaging and 720 nm (3.0–3.5 mW under the objective) for uncaging. Green fluorescence emission was collected using an immersion objective (LUMPlan FL N 60×, numerical aperture 1.0, Olympus), reflected by a dichroic mirror (565 nm LP) and passed a filter (Chroma, 510nm/70-2p) before entering the fast photoelectron multiplier tubes (PMT) (H7422-40p; Hamamatsu). Fluorescence images were acquired and quantified using TimeHarp 260 Pico card (PicoQuant, Inc) and custom-built software, FLIMage (Ver 2.0.20) written with #C (https://github.com/ryoheiyasuda/FLIMage_public). Fast-rate simultaneous image acquisitions with uncaging were collected by 128 × 128 pixels at a single z plane without averaging per frame (frame rate 3.91 Hz). Image acquisitions for slow-rate imaging were collected by 128 × 128 pixels as a z stack of five frames with 1 μm distance in each frame and averaging 6 scans per frame (frame rate 0.65 Hz). Maximum intensity projection images were generated by a z-stack of five frames for slow-rate imaging. MNI-caged L-glutamate (4-methoxy-7-nitroindolinyl-caged L-glutamate, Tocris) was uncaged with a train of 8–10 ms laser pulses (under the objective, 30 times at 0.5 Hz) near a spine of interest. Experiments were performed at room temperature (24–26 °C) in ACSF solution containing: NaCl (127 mM), KCl (2.5 mM), $NaHCO_3$ (25 mM), NaH_2PO_4 (1.25 mM), $CaCl_2$ (4 mM), glucose (25 mM), tetrodotoxin (1 μM), and 4-MNI-caged L-glutamate (4 mM), bubbled with 95% O_2 and 5% CO_2. We examined secondary or tertiary branches of apical dendrites of cultured hippocampal neurons at 21–25 days in vitro. Spine volume change was calculated by F/F_0, in which F_0 is the average spine intensity before stimulation. All values are presented as mean ± SEM. Number of independent measurements (n=[spines/neurons]). Mann-Whitney's U test and Two-way ANOVA, followed by Turkey's test were used to compare grouped data sets for fast and slow-rate imaging, respectively (Prism 9.4.1, GraphPad). Data were excluded if signs of

poor cellular health or procedural artifacts were apparent (for example, dendritic blebbing, and displacement of dendrites).

Antibodies used in this study

Puromycin (EMD Millipore, MABE342, Lot:3166081, clone 4G11,mouse), alpha-Tubulin (Invitrogen, PA1-38814, Lot:VI3090432, Rabbit), GFP (Novus Biologicals, NB100-1614, Lot:917979, chicken), Map2 (Synaptic systems, 188 004, Guinea Pig), CamKIIα (invitrogen, MA1-048, Lot:UH288077, Mouse), p-CaMKII-T286 (Cell Signaling Technology, #12716S, Lot:5, D21E4, Rabbit), Anti-DIG fab fragment antibody (Roche, 11207733910, Lot:35698000) , beta-actin (Abcam, Ab8227, Lot:LR288171-1, Rabbit), synaptophysin (Abcam, ab32594, Lot: GR198446-4, Rabbit), Vimentin (AbCam, ab137321, Lot:GR3356521-4, Rabbit), eIF2alpha (Cell Signaling Technology,mAb #5324, Lot:3, D7D3, Rabbit), Gapdh (Santa Cruz Biotechnology, sc-32233, Lot:A2319, 6C5, mouse), eIF3G (Novus Biologicals, NB100-93298, Lot:A1, Rabbit), GluR2 (EMD Millipore, MABN71, Lot:3460365, clone L21/32, mouse), P70S6K (Cell Signaling Technology, #9202S, Lot:3, Rabbit), Anti-rabbit IgG HRP-linked (Cell Signaling Technology, #7074S, Lot:31, Goat), Anti-mouse IgG HRP-linked (Cell Signaling Technology, #7076S, Lot:35, Horse), Anti-rabbit Alexa Fluor647 (Invitrogen, A31573, Lot:169297, donkey), Anti-mouse Alexa Fluor546 (invitrogen, A10036, Lot:771559, donkey).

Puromycin Labeling

DIV14 hippocampal neurons from mice were either transfected with SLAMR-OE, SLAMR-KD, or control NC-GFP via lipofection. After 72 hrs, before ICC, neurons were treated with 1µm Puromycin for 15 min to block protein synthesis and label newly synthesized proteins. Then they were fixed with 4%PFA in PBS, washed 3 times, and blocked with 10% horse serum in PBS-T (0.1%) for 1 hour. After blocking, neurons were labeled with anti-GFP antibody (1:100, chicken), anti-Puro antibody (1:200, mouse), anti-alpha-tubulin antibody (1:1000, rabbit), and incubated overnight at 4°C. After 3 washes, the cells were incubated with the corresponding secondary antibodies tagged with anti-chicken Alexa 488, anti-mouse Alexa 546, and anti-rabbit Alexa-647. After 3 more washes, the slides

were mounted, with a mounting medium containing DAPI to identify cells. Images were captured in Olympus FV 1000 with an average of 15 stacks per image in 3 channels (green, red, and far red).

After imaging, the Intensity calculator plugin was used in ImageJ to quantify the Corrected Total Cell Fluorescence (CTCF) from both the cell body and randomly selected areas of dendrites of neurons.

RNA pull-down assay: Proteomic, transcriptomic, and protected fragments analysis

Native proteomic and transcriptomic analysis

Sense SLAMR RNA sequence was full-length cloned for this experiment into a pCR-II-TOPO vector from a template (Thermo Fisher), linearized, and 5’ end Biotin-labeled (Roche) by *in vitro* transcription using a SP6 RNA polymerase enzyme for the sense strand and T7 for antisense strand that was used as a control.

For this experiment, we follow the same procedure described before in Grinman et al., 2021. C57BL6 mice (8 weeks old, n=5) were sacrificed by cervical dislocation, the brain was removed from the skull, briefly washed with D-PBS and the hippocampus was dissected and ground in lysis buffer (Tris-HCl 50mM, NaCl 150mM, EDTA, 0.5M, NP-40 0.25%, DTT 1mM, Protease inhibitor (Sigma), Phosphatase inhibitors cocktails 2 & 3 (Sigma), Superase-In RNAse inhibitor (Thermo fisher), UltraPure BSA (MCLAB). Tissue was incubated in a rotator with the lysis buffer for 1hr to finish the lysate and centrifuge at 1200xg for 20 min to remove the debris. Hippocampal lysate supernatant was transferred to a new tube and incubated with 2µl of the sense or antisense biotinylated probes for 2 hrs. During that time, the streptavidin-coated magnetic beads (NEB) were washed with the same lysis buffer and blocked with yeast tRNA and glycogen (0.2mg/ml) for 1 hr. Blocking medium was replaced with lysis buffer before use. 10ul of pre-cleared magnetic beads were added to each sample and incubated in slow rotation for 1 hr. After finishing the incubation, the beads were washed 3 times with the lysis buffer and plated in a 24-well plate for UV/Cross-link (320 mJ/cm^2). Then, the samples were placed again into 1.5 ml tubes and washed with a high stringency buffer (Tris-HCl 50mM, NaCl 600mM,

EDTA 0.5M, NP-40 0.25%, DTT 1mM, Superase-In RNAse inhibitor, Protease inhibitor, Phosphatase inhibitor cocktails 2 &3). Finally, the beads with the RNA/protein complexes were diluted in Tris buffer + 0.15% SDS and incubated at 37°C for 1 hr. For RNA experiments beads were incubated with trizol and isolated using the Direct-zol RNA miniprep kit (Zymo). The RNA was processed for RNAseq and analyzed following the same procedure described before by the TRSI Genomics Core.

Bound proteins were eluted by heating to 96°C in the presence of Laemmli sample buffer for 10 min for denaturation. After finishing this process, the samples were loaded in a pre-cast Tris-glycine polyacrylamide gradient gel (4-15%, 1mm, Bio-rad) to perform a denaturing SDS-PAGE. The gel was washed with HPLC water, fixed with a solution of 50% Ethanol-5% Acetic Acid for 1 h. After that, the bands were sectioned for each sample (sense and antisense). Then, whole gel lanes were in-gel digested with trypsin (Pierce Biotechnology, Rockford, IL) for 3 hours at 37ºC using ProteaseMax™ Surfactant trypsin enhancer following reduction and alkylation with dithiothreitol and iodoacetamide, respectively, according to the manufacturer's instructions (Promega Corporation, Madison, WI). LC-MS/MS analysis of extracted peptides was subsequently carried out using an Orbitrap Fusion Tribrid mass spectrometer, following 2mg capacity ZipTip (Millipore, Billeric, MA) C18 sample clean-up according to the manufacturer's instructions. Peptides were eluted from an Acclaim PepMap™ RSLC nano Viper analytical column (75-μm ID × 15 cm, Thermo Scientific, San Jose, CA) using a gradient of 5-25% solvent B (80/20 acetonitrile/water, 0.1% formic acid) in 180 min, followed by 25-44% solvent B in 60 min, 44-80% solvent B in 0.10 min, and finally a 5 min hold of 80% solvent B. All flow rates were 300nL/min delivered using a nEasy-LC1000 nano liquid chromatography system (Thermo Fisher Scientific, San Jose, CA). Solvent A consisted of water and 0.1% formic acid. Ions were created at 1.8kV using the Nanospray Flex™ ion source (Thermo Fisher Scientific, San Jose, CA). Data dependent scanning was performed by the Xcalibur v 4.0.27.10 software using a survey scan at 120, 000 resolution in the Orbitrap analyzer scanning mass/charge (m/z) 380-2000 followed by higher-energy collisional dissociation (HCD) tandem mass spectrometry (MS/MS) at a normalized collision energy of 30% of the most intense ions at maximum speed, at an automatic gain control of 1.0E4. Precursor ions were selected by the

monoisotopic precursor selection (MIPS) setting to peptide and MS/MS was performed on charged species of 1-8 or 2-8 charges at a resolution of 30,000. Dynamic exclusion was set to exclude ions after two times within a 30sec window, for 20sec. Tandem mass spectra were searched against a mouse FASTA database of reviewed proteins from UniprotKB downloaded on August 16, 2018, to which additional common contaminant proteins (e.g., trypsin; obtained at ftp://ftp.thegpm.org/fasta/cRAP) were appended. All MS/MS spectra were searched using Thermo Proteome Discoverer 1.4.1.14 (Thermo Fisher Scientific) considering fully tryptic peptides with up to 2 missed cleavage sites. Variable modifications considered during the search included methionine oxidation (15.995 Da), and asparagine and glutamine deamidation (0.984 Da). Cysteine carbamidomethylation (57.021 Da) was considered a static modification. At the time of the search, the protein database used, contained 17,068 entries. Proteins were identified at 99% confidence with XCorr score cut-offs (Quian et al., 2005) as determined by a reversed database search.

Protected fragments assay

C57BL6 mice brains (8 weeks old, n=6) were used for this study. The tissue was processed for pull-down following the same procedure described above. However, the samples were now incubated with RNAase A (1µl RNAse A, 10µg/µl, Thermo Fisher). After RNAase A incubation the samples were diluted in PBS and the protected fragments were purified and concentrated using sucrose cushion. Then, the supernatant was removed, and the RNA fragments pellet was diluted in Trizol reagent for their purification using the trizol-chloroform RNA purification method. The samples were sequenced by the Scripps Genomics core using HTseq and the reads alignment to transcription using Salmon software.

Western-Blot/Immuno-Blot

For the WB analysis, the protein concentration was determined using a BCA kit. Protein (10-25 µg) was used for WB analysis. The target proteins were detected using primary antibodies (1:1000 anti-eIF3g rabbit) (1:1000 anti-eIF2a rabbit) (1:1000 anti-P70S6k rabbit) (1:5000 β-actin rabbit) (1:500 anti-CaMKII mouse) (1:500 anti-pCaMKII rabbit)

(1:2000 anti-Gapdh mouse) (1:1000 anti-Vimentin rabbit) (1:500 anti-GluR2 mouse) (1:1000 anti-Synaptophysin rabbit) then anti-rabbit-HRP or anti-mouse-HRP secondary antibodies at a 1:5000 dilution and then visualized by chemiluminescence (Amersham Biosciences, Piscataway, NJ). The autoradiograms were analyzed by ImageJ.

Immunocytochemistry (ICC)

ICC experiments were performed using mouse primary hippocampal cell cultures in coverslips on DIV14 and/or 72hrs after the transfection. Briefly, culture media was first removed to wash the cells with PBS and fixed in a solution of PBS + 4% PFA (pH 7.4) for 10 min, then washed again 3 times with PBS. After complete removal of PFA, neurons were permeabilized in 0.5% TritonX-100 diluted in PBS for 10 min and incubated in 5% normal goat serum (NGS, Sigma) in PBS for 1 hr and incubated with primary antibody (1:1000 anti-MAP2 Guinea Pig) at 4°C over-night. The day after cells were rinsed 3 times in PBS to remove the primary antibody and incubated with the appropriate secondary Alexa antibodies 594nm, 488nm, or 405nm (1:500 Molecular Probes) 1h at RT. Finally, the cells were washed with PBS and mounted in slides using Fluoro gel II (Electron Microscopy Sciences) for imaging.

Organotypic hippocampal slice cultures and transfection

Organotypic hippocampal slice cultures were prepared from wild-type postnatal 5-7 day-old C57BL/6N mouse pups of both sexes as previously described (Stoppini et al., 1991). In brief, the mice were deeply anesthetized with isoflurane, and quickly decapitated. The brains were removed, and hippocampi were dissected into 350 µm thick coronal hippocampal slices using a McIlwain tissue chopper (Ted Pella, Inc) and plated on hydrophilic PTFE membranes (Millicell, Millipore) fed by culture medium containing MEM medium (Life Technologies), 20% horse serum, 1mM L-Glutamine, 1mM $CaCl_2$, 2mM $MgSO_4$, 12.9mM D-Glucose, 5.2mM $NaHCO_3$, 30mM HEPES, 0.075% Ascorbic Acid, 1 µg/ml insulin. The slices were incubated at 37 °C in 5% CO_2. After 7 days in vitro (DIV), CA1 pyramidal neurons were transfected with biolistic gene gun using 1.6 µm gold beads (8–12 mg) coated with mixed plasmids of CaMKIIα sensor CAG-2dV-Camuiα (Jain et al., 2023) and NC-mCherry (25 ug each), or CaMKIIα sensor and TET-SLAMR-mCherry (25

μg each) (O'Brien and Lummis, 2006). 50% of culture media was replaced every 2-3 days. Doxycycline was applied in culture media at the final concentration of 1 μg/ml after 14 DIV.

Two-photon fluorescence microscopy and two-photon glutamate uncaging for analyses of spine morphology and CaMKIIα activity

MNI-caged glutamate uncaging and timelapse structural imaging of spines were performed using a custom-built two-photon (2p) laser microscope as previously described (Colgan et al., 2018). 2p-imaging and uncaging were performed using two Ti-sapphire lasers (Coherent, Cameleon) at wavelengths of 920 nm (1.45–1.55 mW under the objective) for imaging and 720 nm (3.0–3.5 mW under the objective) for uncaging. Green fluorescence emission was collected using an immersion objective (LUMPlan FL N 60×, numerical aperture 1.0, Olympus), reflected by a dichroic mirror (565 nm LP) and passed a filter (Chroma, 510nm/70-2p) before entering the fast photoelectron multiplier tubes (PMT) (H7422-40p; Hamamatsu). Fluorescence images were acquired and quantified using TimeHarp 260 Pico card (PicoQuant, Inc) and custom-built software, FLIMage (Ver 2.0.20) written with #C (https://github.com/ryoheiyasuda/FLIMage_public). In primary dissociated culture imaging, fast-rate simultaneous image acquisitions with uncaging were collected by 128 × 128 pixels at a single z plane without averaging per frame (frame rate 3.91 Hz). Image acquisitions for slow-rate imaging were collected by 128 × 128 pixels as a z stack of five frames with 1 μm distance in each frame and averaging 6 scans per frame (frame rate 0.65 Hz). Maximum intensity projection images were generated by a z-stack of five frames for slow-rate imaging. In organotypic slice culture, image acquisitions were collected by 64 x 64 pixels at a single z plane without averaging per frame (frame rate 7.8 Hz). MNI-caged L-glutamate (4-methoxy-7-nitroindolinyl-caged L-glutamate, Tocris) was uncaged with a train of 8–10 ms (for primary dissociated cultures) and 6 ms (for organotypic cultures) laser pulses (under the objective, 30 times at 0.5 Hz) near a spine of interest. Experiments were performed at room temperature (24–26 °C) in ACSF solution containing: NaCl (127 mM), KCl (2.5 mM), NaHCO3 (25 mM), NaH2PO4 (1.25 mM), CaCl2 (4 mM), glucose (25 mM), tetrodotoxin (1 μM), and 4-MNI-caged L-glutamate

(4 mM), bubbled with 95% O2 and 5% CO2. We examined secondary or tertiary branches of apical dendrites of cultured hippocampal neurons at 21–25 days in vitro. Pyramidal neurons co-expressing mCherry and CaMKIIα sensor (CAG-2dV-Camuiα (Jain et al,. 2023)), in the CA1 region at organotypic culture were imaged 21-28 days after transfection. Spine volume change was calculated by F/F0, in which F0 is the average spine intensity before stimulation. All values are presented as mean ± SEM. Number of independent measurements (n=[spines/neurons]). Mann-Whitney's U test and Two-way ANOVA, followed by Turkey's test were used to compare grouped data sets for fast and slow-rate imaging, respectively (Prism 9.4.1, GraphPad). Data were excluded if signs of poor cellular health or procedural artifacts were apparent (for example, dendritic blebbing, and displacement of dendrites).

2-photon fluorescence lifetime imaging analysis

To measure the change in the fluorescence lifetime of a CaMKIIα FLIM-FRET sensor, we fit a fluorescence lifetime curve summing each target spine image before stimulation with a double exponential function convolved with the Gaussian pulse response function as follows:

$F(t) = F_0[P_D\, H(t, t_0, \tau_D, \tau_G) + P_{AD}\, H(t, t_0, \tau_{AD}, \tau_G)]$

where τ_{AD} is the fluorescence lifetime of the donor bound with the acceptor, P_D and P_{AD} are the fraction of free donor and the donor undergoing FRET with the acceptor, respectively, and $H(t)$ is a fluorescence lifetime curve with a single exponential function convolved with the Gaussian pulse response function as follows:

$$H(t, t_0, t_D, t_G) = \frac{1}{2}\exp\left(\frac{\tau_G^2}{2\tau_D^2} - \frac{t - t_0}{\tau_i}\right)\text{erfc}\left(\frac{\tau_G^2 - \tau_D(t - t_0)}{\sqrt{2}\tau_D\tau_G}\right),$$

in which τ_D is the fluorescence lifetime of the free donor, τ_G is the width of the Gaussian pulse response function, F_0 is the peak fluorescence before convolution, t_0 is the time offset, and erfc is the complementary error function.

We fixed τ_D to the fluorescence lifetime obtained from free mEGFP (2.6 ns) and τ_{AD} to these values to obtain stable fitting. To generate the fluorescence lifetime image, we calculated the mean photon arrival time, $<t>$, in each pixel as follows:

$\langle t \rangle = \int tF(t)\, dt / \int F(t)\, dt,$

Then, the mean fluorescence lifetime, $\langle\tau\rangle$, is calculated as the mean photon arrival time minus offset arrival time, t_0, which is obtained by fitting the whole image:

$\langle\tau\rangle = \langle t\rangle - t0.$

Change in lifetime is calculated as mean fluorescence lifetime, $\langle\tau\rangle$, in ROI subtracted by the average lifetime in the ROI before stimulation.

CaMKII Activation Assay

CaMKII Activation assay was performed following the manufacturer's protocol using the Cyclex CaM Kinase II assay kit. Briefly, for the generation of the standard curve, fixed concentration of CaMKII positive control (Cat# CY-E1173) (1.5 m units/µL) were added to each microtiter well with varying concentrations of 100X Calmodulin (Cat# CY-E1173) (25, 10, 5 and 0 ng/ml). The kinase reaction was initiated by the addition of 90 µL of Kinase Reaction Buffer (Ca/CaM plus) per well, covered with plate sealer, and incubated at 30°C for 30 minutes. Then, wells were washed five times with wash buffer making sure each well was filled completely. Residual wash buffer was removed by gentle tapping or aspiration. Next, 100 µL of HRP conjugated Detection Antibody was pipetted into each well, covered with a plate sealer, and incubated at room temperature (25°C) for 60 min. Any unused conjugate was discarded. This was followed by another five washes with wash buffer. After this, 100 µL of Substrate Reagent was added to each well and incubated at room temperature (25°C) for 5–15 min. Finally, 100 µL of Stop Solution was added to each well in the same order as the previously added Substrate Reagent. The absorbance was measured in each well using a spectrophotometric plate reader (TECAN) at a wavelength of 450nm.

For the SLAMR-mediated CaMKII Activation, sense, and antisense, full length in-vitro transcribed SLAMR RNA (1:10 dilution) were added to varying Calmodulin concentrations as above. Additional controls were antisense SLAMR and IVT reactions with no templated DNA added (NTC) (n=4 biological replicates per group).

Behavior

Before starting each behavioral training, male C57BL6 mice were acclimated to the transportation and waiting room for one hour followed by a handling manipulation of 1 min per mouse for three days.

Basal behavior

To analyze transcriptional changes in different hippocampal subregions induced by learning/memory processes, two different experiments were carried out:

Contextual Fear Conditioning (CFC): CFC was performed using a modified version of Noldus PhenoTyper Model 300 chambers (30x30x40) (Leesburg, VA) that includes a grid floor (30x30) prepared with a shock delivered system (Shock Scrambler ENV-414S; Med Associates, ST. Alvans, VT) and equipped with a white light inside (Rizzo et al., 2017). Automated tracking and shock delivery control were performed using EthoVision 8.5 software (Noldus Information Technology, Leesburg, VA). Each chamber was cleaned with 70% ethanol before and after finishing each training session. 72db white noise was played in the room to mask any unintended noise that may be added to the context. During the fear conditioning training session, mice received three 2 s 0.75 mA scramble foot shocks 2.5, 3.5, and 4.5 min after placement into the chamber. Mice were promptly removed from the chamber after 5.5 min. Mice were divided into 3 different groups: those who received the training with the three shocks (Context + Shock group), those who were exposed to the context without the shock (Context alone group), and the third group who received a single and immediate shock (2s) and were quickly removed from the box (Immediate Shock group) (Rizzo et al., 2017). All the mice were sacrificed 1 hr after finishing their respective training.

Morris Water Maze (MWM): MWM consisted of a circular tank of 120 cm diameter filled with 22°C water located in a white light-illuminated room with visible external cues. The water was colored with a non-toxic white paint to hide the position of the platform. The escape platform consists of a 5 cm diameter plastic circle that was submerged 1.5 cm under the water and separated 8 cm from the tank wall. During acquisition/training trials mice were trained to escape from water by swimming from variable starting points around

the tank to the hidden platform and allowed to remain there for 15s with a limited exploration time of 1 min for each trial of 4 total training trials a day. After each trial, mice were dried and returned to their home cages. All sessions were recorded by a video camera located above the tank and analyzed using the tracking software EthoVision 8.5. For basal line experiments, mice were divided into three different groups: 1 week of swimming training, 1 day of training, and a swimming session without platform. All the mice were sacrificed 1 hr after finishing the training.

Genetic manipulation of SLAMR

8 weeks old C57BL6 mice were stereotaxically implanted with bilateral cannulas for CA1 or individual cannulas for CA3. Mice were anesthetized with 1% Isoflurane and secured in a Kopf stereotaxic apparatus. For dorsal CA1 infusion, bilateral stainless-steel guide cannula (26G, Plastics one) were implanted using the coordinates AP= -2, ML=±1, and DV =-1 (from the surface of the skull on bregma). For dorsal CA3 infusion, individual stainless-still guided mini cannulas (26G, Plastics one) were implanted on each side of the brain using the coordinates AP=-1.95, ML=±2.5, and DV=-1.5. Clearance through the guide cannula was maintained with 33G obturators (Plastics One) without projection beyond the tip of the cannula and protected with a dust cap (Plastics One). After surgery, animals were given 1 week of recovery during which they were monitored daily to check their health condition. Animals with abnormal motor behavior were excluded from the experiment. Gapmer infusions were delivered into the target area using an infusor with 1 mm of projection below the cannula, 0.5μl of Gapmer (25 pmol per side, 50 pmol total) was delivered in a 0.1μl/min ratio and let 5min for diffusion per side of the mouse hippocampus. 3 days before the infusions mice were acclimated to the procedure room for 1 hour followed by 1 min of handling and the manipulation of the cannula (remove/replace caps and dummies) to habituate the mice to this manipulation.

CFC: Three different experiments were designed to study the role of D17Rik in different memory aspects: Acquisition, Consolidation, Extinction, and Recall. Each of these experiments was comprised of 3 different experimental groups: Sham, Negative Control Gapmer, and SLAMR_Gapmer. The training was performed following the same protocol described before in this work. For acquisition evaluation, mice were tested in the same

context 1 hr after finishing the training without any shock delivery. Learning was measured by the total percentage of freezing in blocks of 1 min for 5 min total. For long-term memory consolidation, mice were tested in the same context 24 hrs after training without any shock delivery, measured by total % of freezing behavior for 5 min in blocks of 1 min. For extinction, mice spent 30 min in the same context of training without any shock delivery. The extinction was measured by % of freezing time by blocks of 5 min for 30 min total. Recall test after extinction was measured 24hr after finishing the extinction training by measuring the freezing time during a 5 min test in blocks of 1 min.

MWM: For this experiment mice were divided into three different groups: Sham, Negative Control Gapmer, and SLAMR_Gapmer. 72hrs after the Gapmers infusion, All the mice receive 7 days of training with 4 trials each day. 24hrs after finishing the training the long-term memory consolidation was evaluated in a single test session. For this test, the platform was removed from its position and the mice were placed in a new starting point for a single trial of 1 min. The total latency time, distance, and velocity was used as a measure of learning and the time spent in the target quadrant of the platform was used to indicate the long-term memory consolidation.

Histological analysis

To verify the position of the cannula and the point of delivery location for the reagents, we perform a cresyl violet staining and light microscope analysis. After finishing each experiment all the mice were sacrificed and the brain was removed from the skull and placed in a 4% PFA solution. After 24hr, the brains were transferred to a 30% sucrose solution for 48hrs and stored in cryomolds with OCT compound (Tissue-Tek, Sakura Finetek) at -80°C before sectioning. Then, the hippocampus was cryosectioned in 35μm coronal slides and mounted in superfrost pre-coated slides (Fisher brand). To localize the placement of the cannula and the infuser the slides were stained following the cresyl-violet method and analyzed by light-microscope (AF6000 Leica Microsystem). Only those mice who present a bilateral infusion in the dorsal CA1 were included in the study.

SUPPLEMENTARY FIGURES

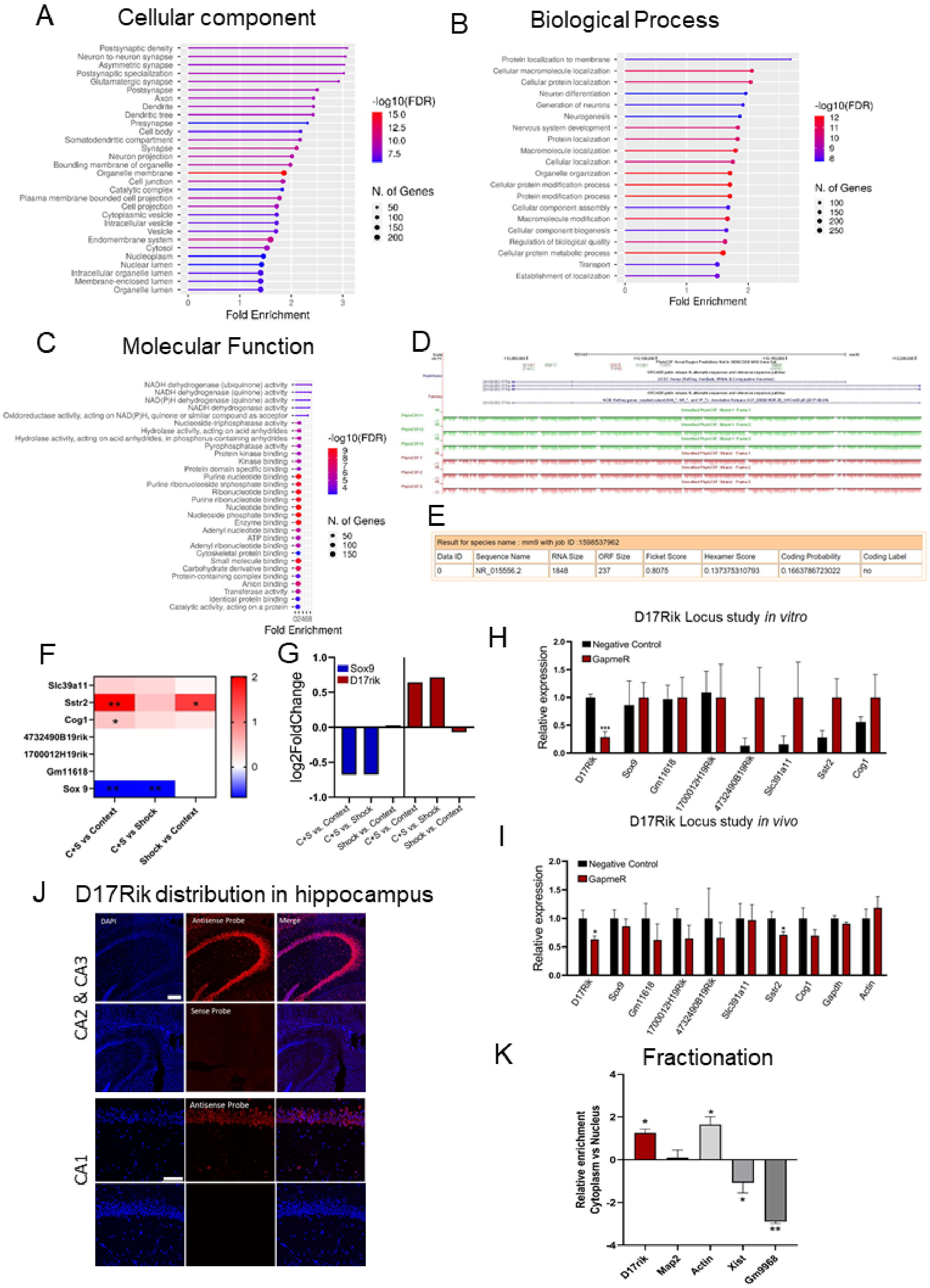

A
Cellular component
Postsynaptic density
Neuron to neuron synapse
Asymmetric synapse
Postsynaptic specialization
Glutamatergic synapse
Postsynapse
Axon
Dendrite
Dendritic tree
Presynapse
Cell body
Somatodendritic compartment
Synapse
Neuron projection
Bounding membrane of organelle
Organelle membrane
Cell junction
Catalytic complex
Plasma membrane bounded cell projection
Cell projection
Cytoplasmic vesicle
Intracellular vesicle
Vesicle
Endomembrane system
Cytosol
Nucleoplasm
Nuclear lumen
Intracellular organelle lumen
Membrane-enclosed lumen
Organelle lumen
Fold Enrichment
-log10(FDR)
N. of Genes
B
Biological Process
Protein localization to membrane
Cellular macromolecule localization
Cellular protein localization
Neuron differentiation
Generation of neurons
Neurogenesis
Nervous system development
Protein localization
Macromolecule localization
Cellular localization
Organelle organization
Cellular protein modification process
Protein modification process
Cellular component assembly
Macromolecule modification
Cellular component biogenesis
Regulation of biological quality
Cellular protein metabolic process
Transport
Establishment of localization
Fold Enrichment
-log10(FDR)
N. of Genes
C
Molecular Function
NADH dehydrogenase (ubiquinone) activity
NADH dehydrogenase (quinone) activity
NAD(P)H dehydrogenase (quinone) activity
NADH dehydrogenase activity
Oxidoreductase activity, acting on NAD(P)H, quinone or similar compound as acceptor
Nucleoside-triphosphatase activity
Hydrolase activity, acting on acid anhydrides
Hydrolase activity, acting on acid anhydrides, in phosphorus-containing anhydrides
Pyrophosphatase activity
Protein kinase binding
Kinase binding
Protein domain specific binding
Purine nucleotide binding
Purine ribonucleoside triphosphate binding
Ribonucleotide binding
Purine ribonucleotide binding
Nucleotide binding
Nucleoside phosphate binding
Enzyme binding
Adenyl nucleotide binding
ATP binding
Adenyl ribonucleotide binding
Cytoskeletal protein binding
Small molecule binding
Carbohydrate derivative binding
Protein-containing complex binding
Anion binding
Transferase activity
Identical protein binding
Catalytic activity, acting on a protein
Fold Enrichment
-log10(FDR)
N. of Genes
D
E
Result for species name : mm9 with job ID :1598537962
Data ID | Sequence Name | RNA Size | ORF Size | Ficket Score | Hexamer Score | Coding Probability | Coding Label
0 | NR_015556.2 | 1848 | 237 | 0.8075 | 0.137375310793 | 0.1663786723022 | no
F
Slc39a11
Sstr2
Cog1
4732490B19rik
1700012H19rik
Gm11618
Sox 9
C+S vs Context
C+S vs Shock
Shock vs Context
G
Sox9
D17rik
log2FoldChange
C+S vs. Context
C+S vs. Shock
Shock vs. Context
H
D17Rik Locus study in vitro
Negative Control
GapmeR
Relative expression
D17Rik
Sox9
Gm11618
1700012H19Rik
4732490B19Rik
Slc391a11
Sstr2
Cog1
I
D17Rik Locus study in vivo
Negative Control
GapmeR
Relative expression
D17Rik
Sox9
Gm11618
1700012H19Rik
4732490B19Rik
Slc391a11
Sstr2
Cog1
Gapdh
Actin
J
D17Rik distribution in hippocampus
DAPI
Antisense Probe
Merge
Sense Probe
CA2 & CA3
CA1
K
Fractionation
Relative enrichment Cytoplasm vs Nucleus
D17rik
Map2
Actin
Xist
Gm9968

Supplementary Figure S1. D17Rik KD does not affect the expression of its neighbor genes. A-C GO analysis of cellular component (A), biological process (B), and molecular function (C). These results are based on the analysis of significant genes in the RNAseq data from C+S condition that overlap between C+S vs Context and C+S vs Shock comparisons. **D.** UCSC genome browser with PhyloCSF 6-frame translation indicates no predicted open reading frames for D17Rik. **E.** CPAT analysis. Coding Potential Assessment Tool identified a single ORF with non-coding potential in D17Rik. **F.** Heat-map representing significant changes in the expression of genes in the D17Rik locus detected in CA1 after CFC by RNAseq. **G**. Plot indicating log2Fold changes in D17Rik and Sox9 between different groups after fear conditioning. **H.** *In vitro* study of genes in the D17Rik locus study after KD D17Rik in hippocampal cell cultures (n=3 per condition, per group). **I.** *In vivo* study of genes in D17Rik locus after silencing D17Rik in dorsal CA1 of the hippocampus (*$p<0.05$, unpaired student t-test, n=7). **J.** Fluorescence in situ hybridization (FISH) shows that the lncRNA D17Rik is expressed in the pyramidal layer of CA2 and CA3 and hilus of the mouse hippocampus. High magnification details from the pyramidal layer in dorsal CA1 indicate a mainly cytoplasmic subcellular localization of D17Rik. Confocal microscopy photomicrographs show D17Rik signal in red and DAPI signal from the nucleus in blue. Scale bars = 100 μm. **K.** These observations are also supported by fractionation studies that show significant enrichment in the cytoplasm of D17Rik in these neurons compared to the nucleus using Actin levels as a reference-normalization element (*p-value <0.05, **p-value= 0.0005, Student t Test, n= 3).

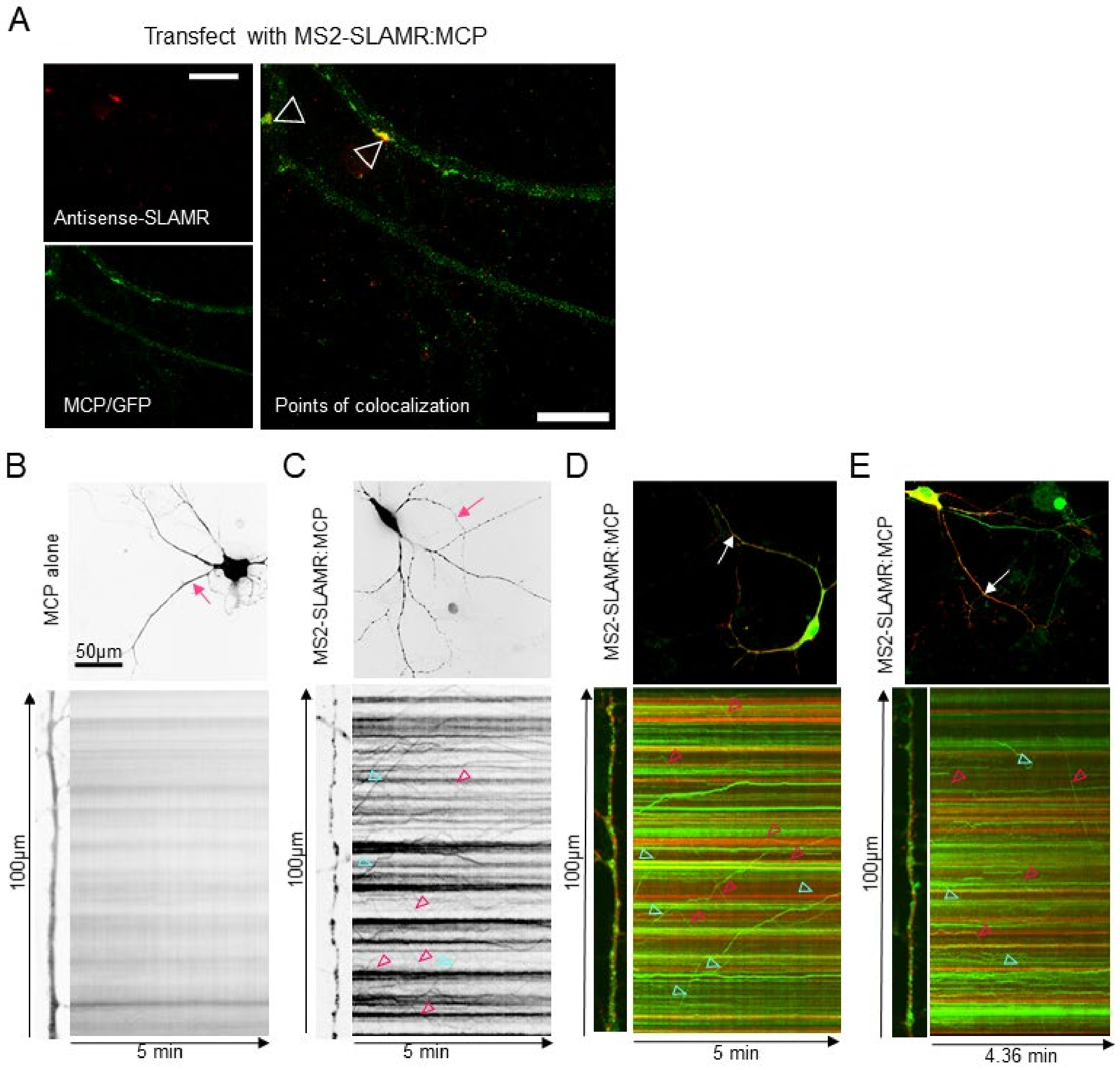

A
Transfect with MS2-SLAMR:MCP
Antisense-SLAMR
MCP/GFP
Points of colocalization
B
MCP alone
50µm
100µm
5 min
C
MS2-SLAMR:MCP
100µm
5 min
D
MS2-SLAMR:MCP
100µm
5 min
E
MS2-SLAMR:MCP
100µm
4.36 min

Supplementary Figure S2. A. FISH showing colocalization of SLAMR (red) and GFP(green) in a distal dendrite of a primary hippocampal neuron transfected with MS2-SLAMR and MCP-GFP. White arrowheads point to regions of colocalization. **B-C.** Cell body images (top), dendrite(left), and kymographs(right) of neurons transfected with MCP alone or MS2-SLAMR+MCP. Pink arrows point to the base of the selected dendrite. Blue arrowheads point to a few selected anterograde tracks, pink arrowheads point to a few selected retrograde tracks. **D-E.** Cell body images (top), dendrite (left), and kymographs (right) of neurons transfected with MS2-SLAMR+MCP (green) and PSD95-mCherry (red). White arrows point to the base of the selected dendrite. Blue arrowheads point to a few selected anterograde tracks, pink arrowheads point to a few selected retrograde tracks.

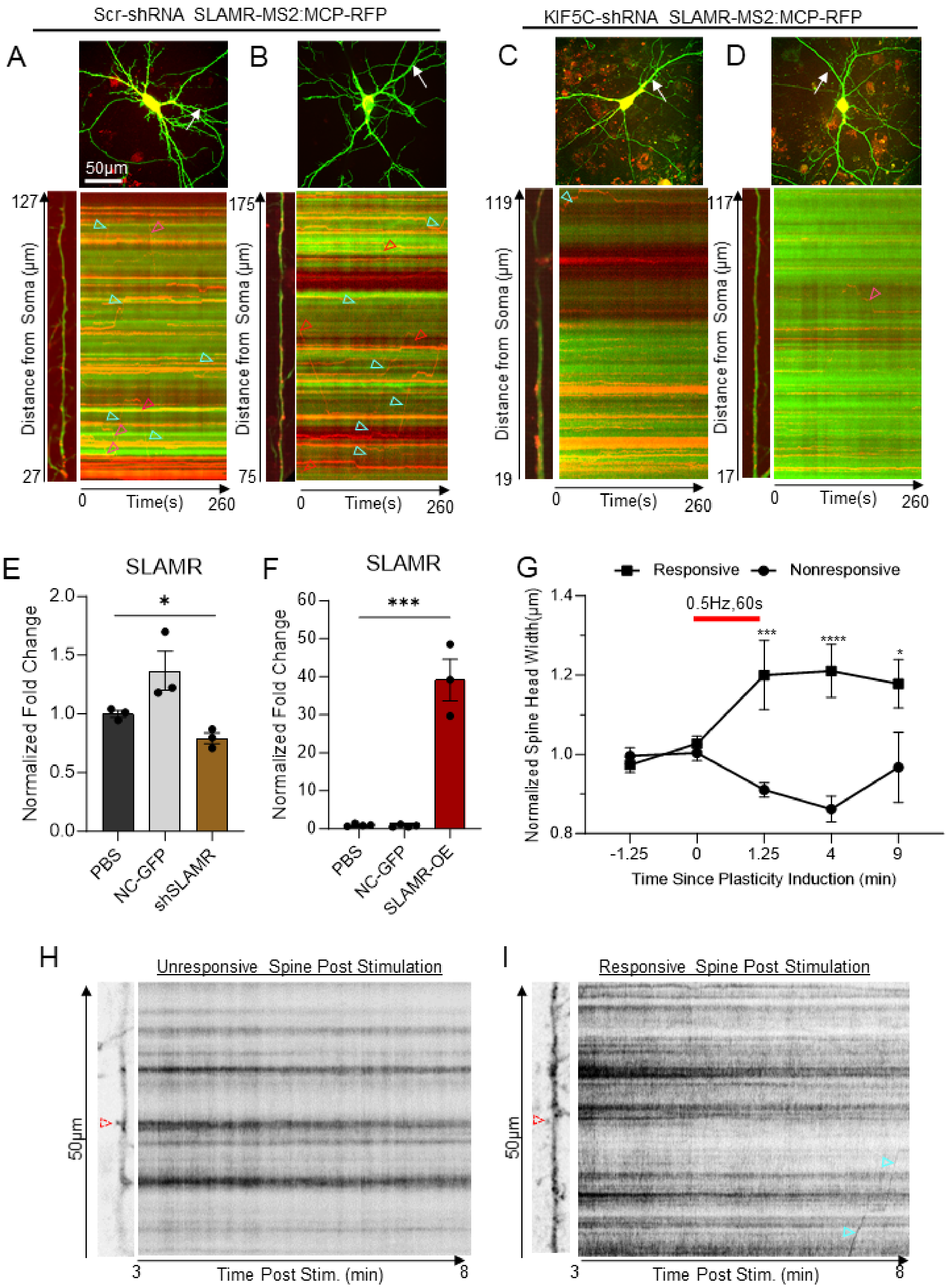

Scr-shRNA SLAMR-MS2:MCP-RFP
KIF5C-shRNA SLAMR-MS2:MCP-RFP
A
B
C
D
50μm
Distance from Soma (μm)
Time(s)
127
27
175
75
119
19
117
17
0
260
E
SLAMR
Normalized Fold Change
PBS
NC-GFP
shSLAMR
F
SLAMR-OE
G
Responsive
Nonresponsive
0.5Hz,60s
Normalized Spine Head Width(μm)
Time Since Plasticity Induction (min)
-1.25
0
1.25
4
9
H
Unresponsive Spine Post Stimulation
I
Responsive Spine Post Stimulation
50μm
Time Post Stim. (min)
3
8

Supplementary Figure S3. A-D. Replicative cell body images, dendrite, and kymographs of neurons transfected with MS2-SLAMR+MCP-RFP (red) and control scrambled Scr-shRNA (green) (**A-B**) or KIF5C-shRNA (green) (**C-D**). Blue arrowheads point to anterograde tracks, pink arrowheads point to retrograde tracks. White arrows in the cell body image point to the base of the selected dendrite. **E.** qPCR analysis validating SLAMR knockdown in cultures transduced by shSLAMR virus. Error bars=SEM. Student's t-test, $^{*}p<0.05$. **F.** qPCR analysis validating SLAMR enrichment in cultures transduced by SLAMR-OE virus. Error bars=SEM. Student's t-test, $^{***}p<0.001$. **G.** Quantification of spine width during the time course of the 30-pulse glutamate uncaging experiment, normalized to the average of the –1.25 and 0 min time points. Error bars=SEM. Two-way ANOVA + Sidak's multiple comparisons test. $^{*}p<0.05$, $^{***}p<0.001$, $^{****}p<0.0001$. **H-I.** Representative dendritic region and kymographs after the stimulation experiment of an unresponsive (**H**) and responsive spine (**I**). Red arrowheads in the dendrite point to the stimulated spine, and blue arrows point to the tracks moving toward the stimulated spine.

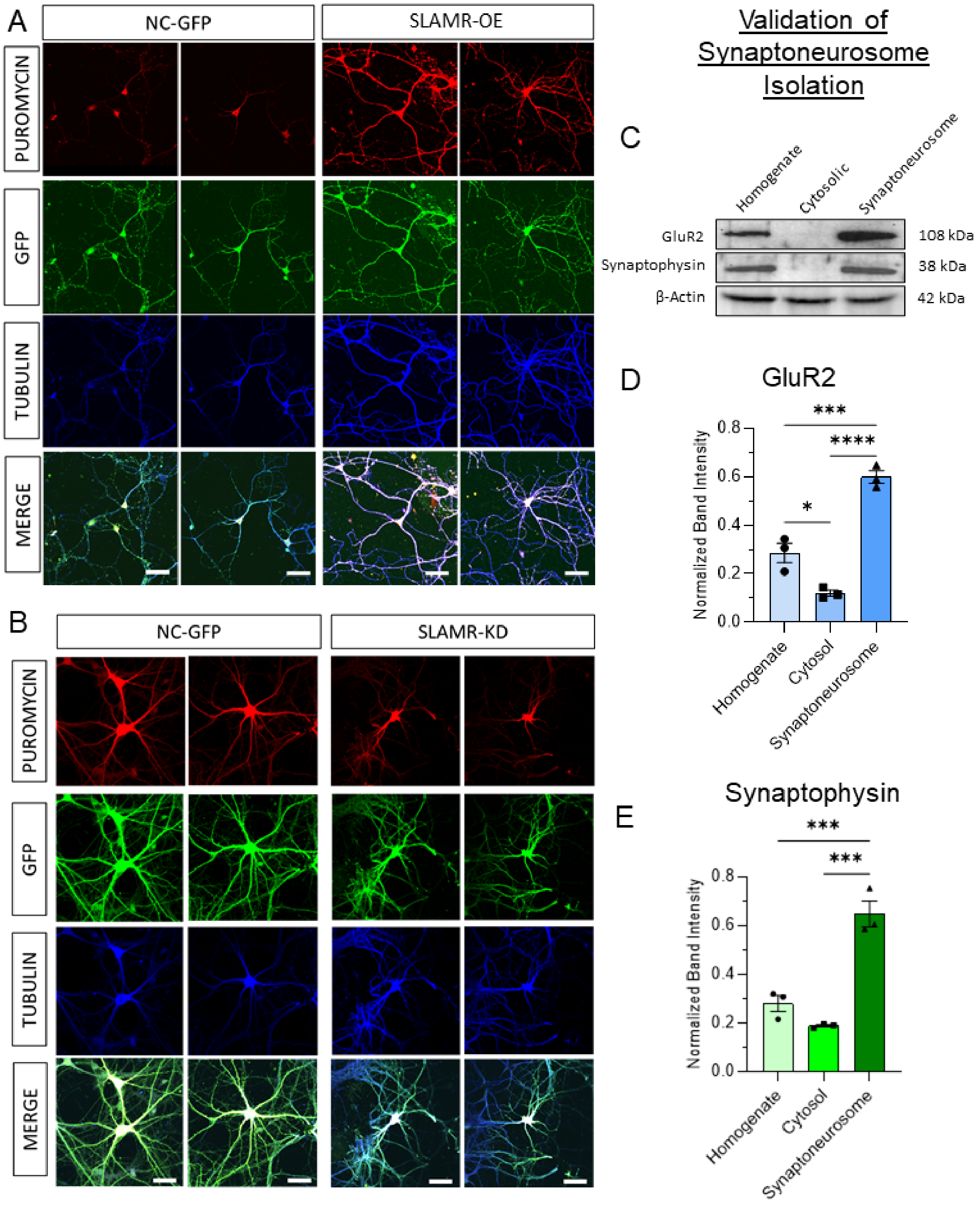

A
NC-GFP
SLAMR-OE
PUROMYCIN
GFP
TUBULIN
MERGE
B
NC-GFP
SLAMR-KD
PUROMYCIN
GFP
TUBULIN
MERGE
Validation of Synaptoneurosome Isolation
C
Homogenate
Cytosolic
Synaptoneurosome
GluR2
Synaptophysin
β-Actin
108 kDa
38 kDa
42 kDa
D
GluR2
Normalized Band Intensity
0.0
0.2
0.4
0.6
0.8
***
****
*
Homogenate
Cytosol
Synaptoneurosome
E
Synaptophysin
Normalized Band Intensity
0.0
0.2
0.4
0.6
0.8
***
***
Homogenate
Cytosol
Synaptoneurosome

Supplementary Figure S4. Validation of Synaptoneurosome Isolation. A. Confocal projection images of NC-GFP and SLAMR-OE transfected images stained for Puromycin, GFP, α-tubulin, and the merged image. Scale Bar=40μm. **B.** Confocal projection images of NC-GFP and SLAMR-KD transfected images stained for Puromycin, GFP, α-tubulin, and the merged image. Scale Bar=20μm. **C.** Western blot analysis of total homogenate from primary hippocampal neurons, the cytosolic fraction, and the synaptoneurosome fraction, probed for GluR2, Synaptophysin, and β-actin. **D, E.** Quantification of C, normalized with β-actin. One-way ANOVA+ Tukey's multiple comparison. *p-value<0.01, **p-value<0.05, ***p-value<0.005. Error bars=SEM.

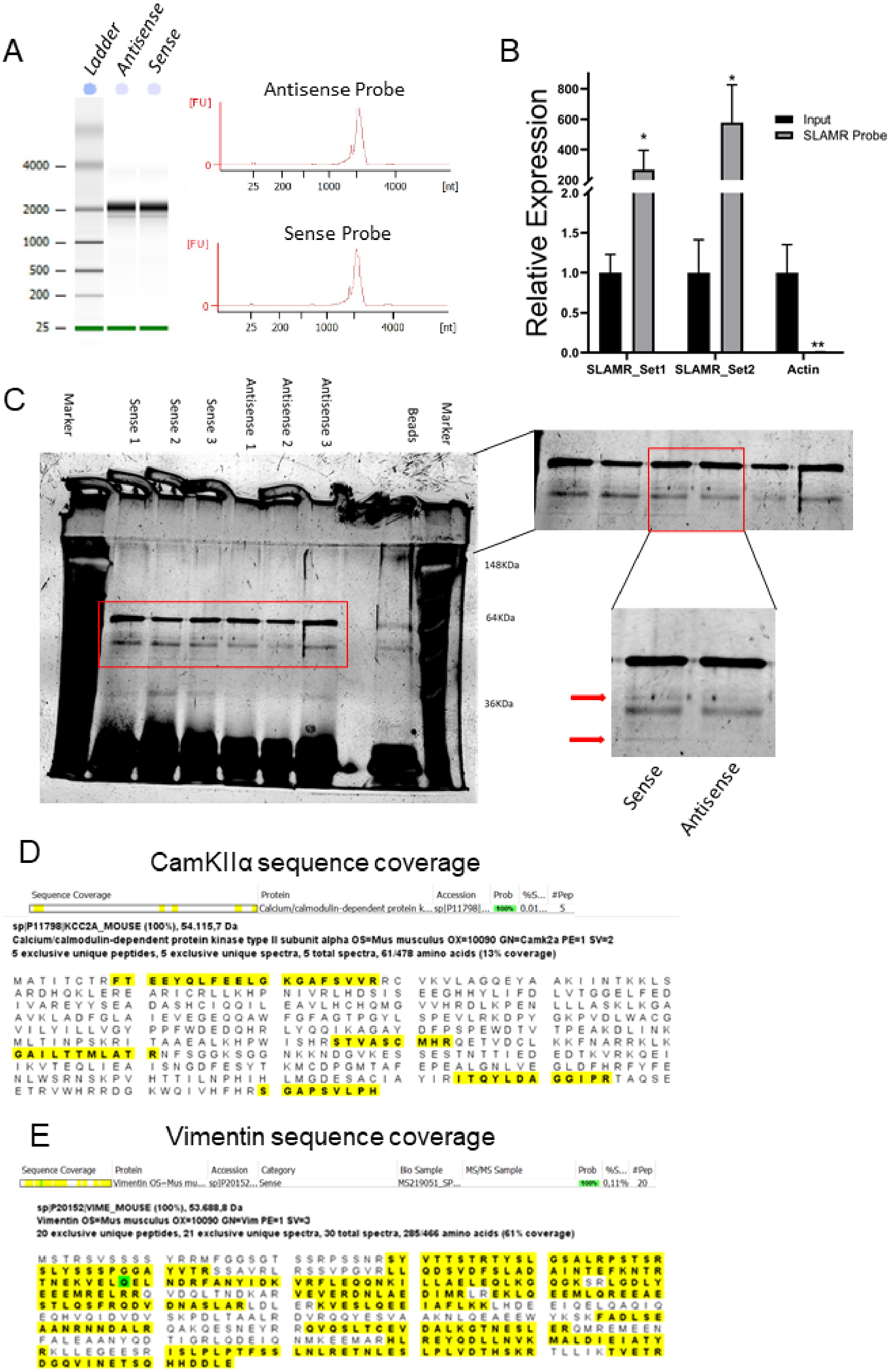
A
Ladder
Antisense
Sense
4000
2000
1000
500
200
25
Antisense Probe
[FU]
0
25
200
1000
4000
[nt]
Sense Probe
B
Relative Expression
Input
SLAMR Probe
SLAMR_Set1
SLAMR_Set2
Actin
C
Marker
Sense 1
Sense 2
Sense 3
Antisense 1
Antisense 2
Antisense 3
Beads
148KDa
64KDa
36KDa
Sense
Antisense
D
CamKIIα sequence coverage
sp|P11798|KCC2A_MOUSE (100%), 54,115,7 Da
Calcium/calmodulin-dependent protein kinase type II subunit alpha OS=Mus musculus OX=10090 GN=Camk2a PE=1 SV=2
5 exclusive unique peptides, 5 exclusive unique spectra, 5 total spectra, 61/478 amino acids (13% coverage)
E
Vimentin sequence coverage
sp|P20152|VIME_MOUSE (100%), 53,688,8 Da
Vimentin OS=Mus musculus OX=10090 GN=Vim PE=1 SV=3
20 exclusive unique peptides, 21 exclusive unique spectra, 30 total spectra, 285/466 amino acids (61% coverage)

Supplementary Figure S5. Pull-down validation of SLAMR. A. Bioanalyzer results testing the length and quality of the SLAMR full-length sense and antisense probes used for pull-down purification. **B**. Bar plots represent the relative gene expression of SLAMR and Actin control of qRT-PCR results indicating enrichment of SLAMR after the RNA isolation from PD samples compared to input and using two different sets of primers for SLAMR. Multiple t-tests, significance determined by Holm-Sidak method, *p-val<0.05, **p-val<0.01. **C.** Representative SDS-PAGE gel after silver staining that shows specific enrichments of proteins in the sense condition compared to the antisense. **D**. CamkIIα sequence coverage on LC-MS/MS showing the unique peptide counts of this protein. **E.** Vimentin sequence coverage on LC-MS/MS showing the unique peptides for this protein.

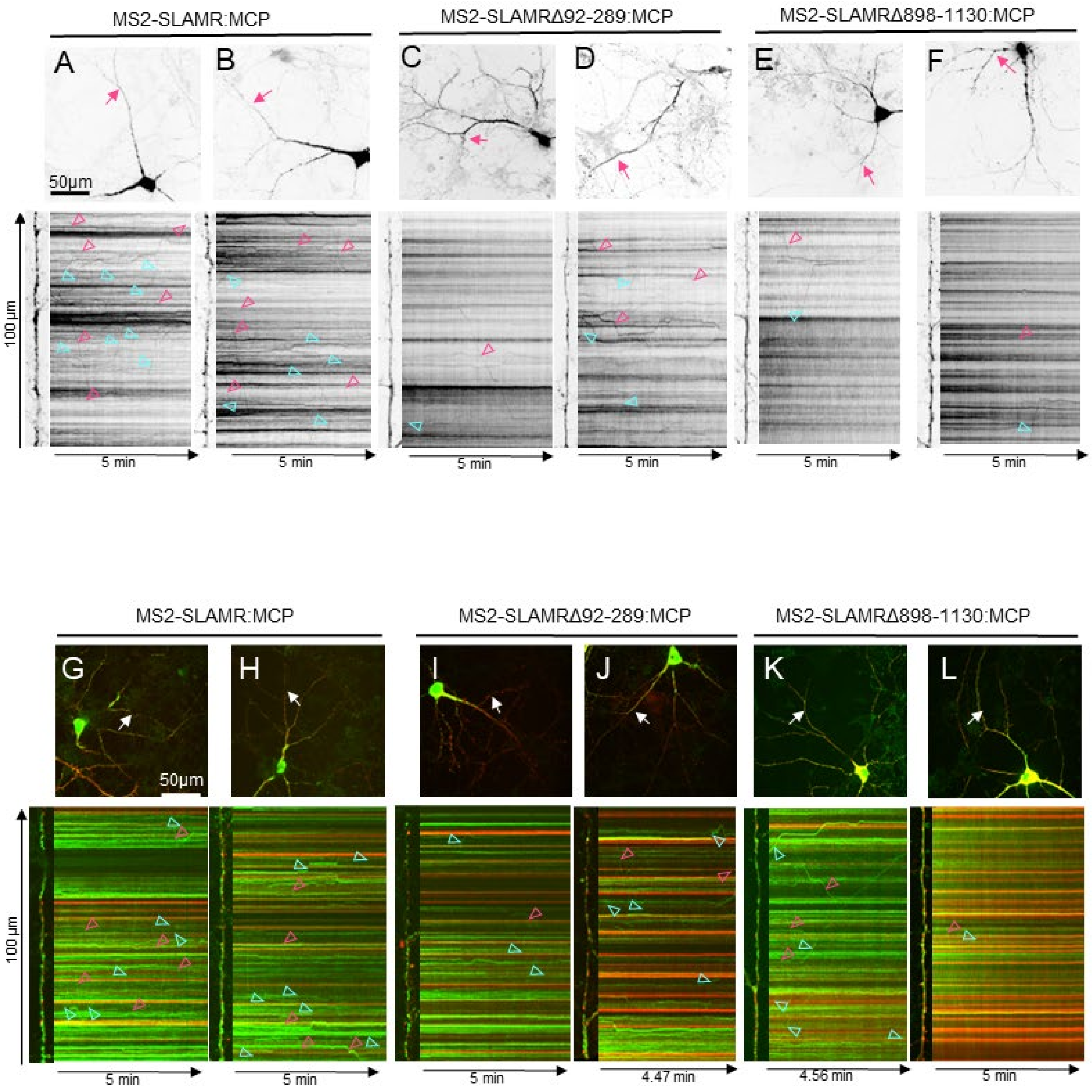
MS2-SLAMR:MCP
MS2-SLAMRΔ92-289:MCP
MS2-SLAMRΔ898-1130:MCP
A
B
C
D
E
F
50μm
100 μm
5 min
5 min
5 min
5 min
5 min
5 min
MS2-SLAMR:MCP
MS2-SLAMRΔ92-289:MCP
MS2-SLAMRΔ898-1130:MCP
G
H
I
J
K
L
50μm
100 μm
5 min
5 min
5 min
4.47 min
4.56 min
5 min

Supplementary Figure S6. A-F Cell body (top), dendrite (left), and kymographs of neurons transfected with MS2-SLAMR (A-B), MS2-SLAMRΔ92-289 (C-D) and MS2-SLAMRΔ898-1130 (E-F). Pink arrows point to the base of the selected dendrite, blue arrowheads point to anterograde tracks, and pink arrowheads point to retrograde tracks. **G-L.** Cell body (top), dendrite (left), and kymographs of neurons transfected with MS2-SLAMR (G-H), MS2-SLAMRΔ92-289 (I-J), and MS2-SLAMRΔ898-1130 (K-L) (green) and PSD95-mCherry(red). White arrows point to the base of the selected dendrite. Blue arrowheads point to a few selected anterograde tracks, pink arrowheads point to a few selected retrograde tracks.

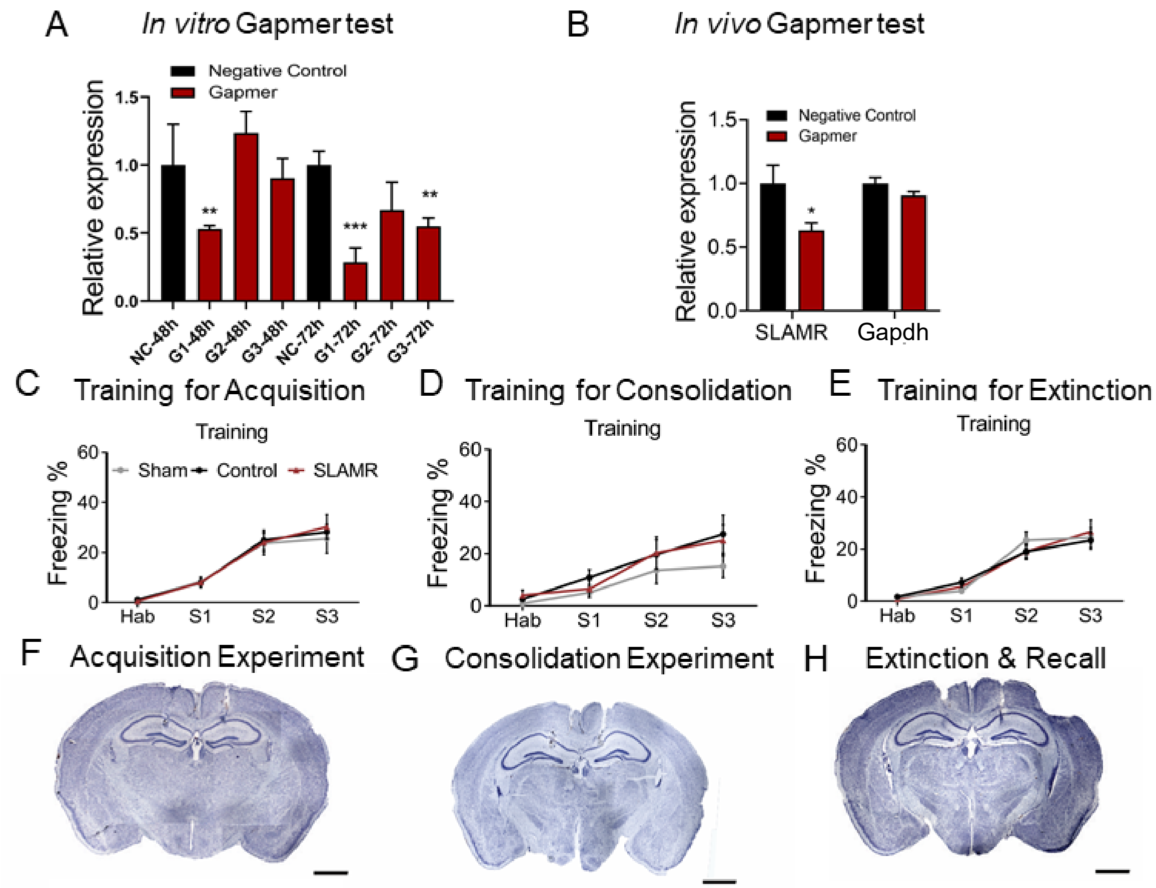

Supplementary Figure S7. SLAMR is not implicated in fear expression. A. Gapmers *in vitro* optimization for silencing SLAMR Dunnett's t-test * p<0.05, **p<0.005, ****p<0.0001. **B.** Gapmers *in vivo* optimization for silencing SLAMR by Bilateral stereotaxic infusions in dorsal CA1 at 72h (n=7). Student *t-test* * p<0.05. **C.** The training curve did not show any significant differences in the expression of fear between groups for acquisition experiments. **D.** Training curve of fear expression representing the % of freezing time for memory consolidation experiment. Data does not indicate significant differences between groups. **E.** Training curve before silencing the expression of SLAMR in CA1 indicates no differences between groups at the basal level. **F.** Photomicrograph represents the position of the infusion into the dorsal hippocampus CA1 for Acquisition experiment on CFC. **G.** Photomicrograph represents the position of the infusion into the dorsal hippocampus CA1 for the consolidation experiment. **H.** Photomicrograph

represents the position of the infusion into the dorsal hippocampus CA1 for Extinction and recall experiments. Scale bars = 1mm

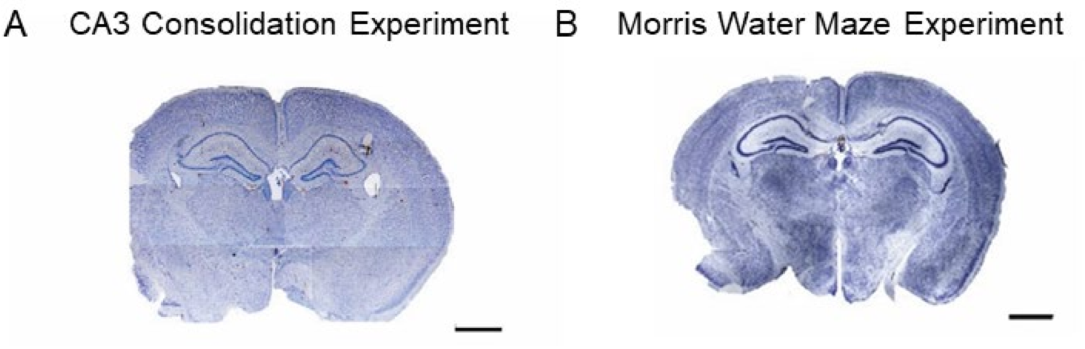

Supplementary Figure S8. A. CA3 infusions of Gapmers for consolidation experiments. **B**. Photomicrograph represents the position of the infusion into the dorsal hippocampus for MWM mice. Scale bar = 1mm.

Supplementary Tables

Supplementary Table S1. List of significant genes from DEseq in dorsal CA1 after CFC training pertaining to Figure 1 and Supplementary Figure S1. Statistical analyses of all data related to Figures 1 and Supplementary Figures S1.

Supplementary Table S2. Statistical analyses of all data related to Figure 2.

Supplementary Table S3. Statistical analyses of all data related to Figure 3 and Supplementary Figure S3.

Supplementary Table S4. Statistical analyses of all data related to Figure 4 and Supplementary Figure S4.

Supplementary Table S5. Statistical analyses of all data related to Figure 5 and Supplementary Figure S5.

Supplementary Table S6 RNA sequencing and LC-MS/MS data from SLAMR Biotin immunoprecipitation pertaining to Figure 6. Statistical analyses of all data related to Figure 6.

Supplementary Table S7. Statistical analyses of all data related to Figure 7. Supplementary Figures S7

Supplementary Table S8. Statistical analyses of all data related to Figure 8.

Supplementary Table S9. Statistical analyses of all data related to Figure 9.

Supplementary Table S10. Statistical analyses of all data related to Figure 10.

Supplementary Table S11. Primers used for all qRT-PCR experiments

Supplementary Table S12. Sequence for sense and antisense in situ probes.

Supplementary movies

File Name: Movie S1

Description: Representative time-lapse movies of top: MCP alone lacking movement and bottom: *MS2-SLAMR* reporter mRNA granules moving in a dendrite of a DIV15 hippocampal neuron. Playback speed 30x real time. Total dendrite length=100μm. Related to Figure 2C(top) and 2D(bottom).

File Name: Movie S2

Description: Representative time-lapse movies of top: MCP alone lacking movement and bottom: *MS2-SLAMR* reporter mRNA granules moving in a dendrite of a DIV15

hippocampal neuron. Playback speed 30x real time. Total dendrite length=100μm. Related to Supplementary Figure S2B (top) and S2C (bottom).

File Name: Movie S3

Description: Two representative time-lapse movies of *MS2-SLAMR* reporter mRNA granules (green) moving and dendritic spines labeled with PSD95-mCherry (red) in dendrites of DIV15 hippocampal neuron. Playback speed 30x real time. Total dendrite length=100 μm. Related to Supplementary Figure S2D(top) and S2E(bottom).

File Name: Movie S4

Description: Top: Two representative time-lapse movies of *MS2-SLAMR:MCP-RFP* reporter mRNA granules (red) moving in DIV15 hippocampal neurons transfected with Scr-shRNA (GFP). Bottom: Two representative time-lapse movies of *MS2-SLAMR:MCP-RFP* reporter mRNA granules (red) moving in DIV15 hippocampal neuron transfected with KIF5C-shRNA (GFP). Playback speed 30x real time. Total dendrite length=100 μm. Related to Supplementary Figures S3A (topmost), S3B (second from top), S3C (second from bottom), and S3D (bottommost).

File Name: Movie S5

Description: Top: Representative time-lapse movie of *MS2-SLAMR:MCP-RFP* MCP lacking movement in a dendrite of a DIV19 hippocampal neuron, taken 3 minutes after spine stimulation in a nonresponsive spine. Bottom: Representative time-lapse movie of *MS2-SLAMR:MCP-RFP* MCP showing movement toward the spine in a dendrite of a DIV19 hippocampal neuron, taken 3 minutes after spine stimulation in a responsive spine. Stimulated spines positioned in the middle. Playback speed 30x real time. Total dendrite length=50μm. Related to Supplementary Figures S3H(top) and S3I (bottom).

File Name: Movie S6

Description: Representative time-lapse movies of *MS2-SLAMR* (top), *MS2-SLAMRΔ92-289* (middle), *MS2-SLAMRΔ898-1130* (bottom) reporter mRNA granules moving in dendrites of DIV15 hippocampal neurons. Playback speed 30x real time. Total dendrite

length 100 µm. Related to Supplementary Figures S6A(top), S6C(middle), and S6E (bottom).

File Name: Movie S7

Description: Representative time-lapse movies of *MS2-SLAMR* (top), *MS2-SLAMRΔ92-289* (middle), *MS2-SLAMRΔ898-1130* (bottom) reporter mRNA granules moving in dendrites of DIV15 hippocampal neurons. Playback speed 30x real time. Total dendrite length 100=µm. Related to Supplementary Figures S6B(top), S6D(middle), and S6F(bottom).

File Name: Movie S8

Description: Representative time-movies of *MS2-SLAMR* (top), *MS2-SLAMRΔ92-289* (middle), *MS2-SLAMRΔ898-1130* (bottom) reporter mRNA granules (green) moving and dendritic spines labeled with PSD95-mCherry (red) in dendrites of a DIV15 hippocampal neurons. Playback speed 30x real time. Total dendrite length 100=µm. Related to Supplementary Figures S6G(top), S6I(middle), and S6K(bottom).

File Name: Movie S9

Description: Representative time-movies of *MS2-SLAMR* (top), *MS2-SLAMRΔ92-289* (middle), *MS2-SLAMRΔ898-1130* (bottom) reporter mRNA granules (green) moving and dendritic spines labeled with PSD95-mCherry (red) in dendrites of a DIV15 hippocampal neurons. Playback speed 30x real time. Total dendrite length=100 µm. Related to Supplementary Figures S6H(top), S6J(middle), and S6L(bottom).
