# Supplementary material for: Synaptically-targeted long non-coding RNA SLAMR promotes structural plasticity by increasing translation and CaMKII activity
Source: Nat Commun. 2024 Mar 27;15:2694. doi: 10.1038/s41467-024-46972-8 (PMC10973417; doi:10.1038/s41467-024-46972-8)
Supplement: Supplementary file 3 — Description of Additional Supplementary Files [file 41467_2024_46972_MOESM3_ESM.pdf]

Description of Additional Supplementary Files

File name: Supplementary Data 1

Description: List of significant genes from DEseq in dorsal CA1 after CFC training pertaining to Figure 1 and Supplementary Figure S1. Statistical analyses of all data related to Figures 1 and Supplementary Figures S1.

File name: Supplementary Data 2

Description: Statistical analyses of all data related to Figure 2.

File name: Supplementary Data 3

Description: Statistical analyses of all data related to Figure 3 and Supplementary Figure S3.

File name: Supplementary Data 4

Description: Statistical analyses of all data related to Figure 4 and Supplementary Figure S4.

File name: Supplementary Data 5

Description: Statistical analyses of all data related to Figure 5 and Supplementary Figure S5.

File name: Supplementary Data 6

Description: RNA sequencing and LC-MS/MS data from SLAMR Biotin immunoprecipitation pertaining to Figure 6. Statistical analyses of all data related to Figure 6.

File name: Supplementary Data 7

Description: Statistical analyses of all data related to Figure 7. Supplementary Figures S7

File name: Supplementary Data 8

Description: Statistical analyses of all data related to Figure 8.

File name: Supplementary Data 9

Description: Statistical analyses of all data related to Figure 9.

File name: Supplementary Data 10

Description: Statistical analyses of all data related to Figure 10.

File name: Supplementary Data 11

Description: Primers used for all qRT-PCR experiments

File name: Supplementary Data 12

Description: Sequence for sense and antisense in situ probes.

File Name: Supplementary Movie 1

Description: Representative time-lapse movies of top: MCP alone lacking movement and bottom: *MS2-SLAMR* reporter mRNA granules moving in a dendrite of a DIV15 hippocampal neuron. Playback speed 30x real time. Total dendrite length=100µm. Related to Figure 2C(top) and 2D(bottom).

File Name: Supplementary Movie 2

Description: Representative time-lapse movies of top: MCP alone lacking movement and bottom: *MS2-SLAMR* reporter mRNA granules moving in a dendrite of a DIV15 hippocampal neuron. Playback speed 30x real time. Total dendrite length=100µm. Related to Supplementary Figure S2B (top) and S2C (bottom).

File Name: Supplementary Movie 3

Description: Two representative time-lapse movies of *MS2-SLAMR* reporter mRNA granules (green) moving and dendritic spines labeled with PSD95-mCherry (red) in

dendrites of DIV15 hippocampal neuron. Playback speed 30x real time. Total dendrite length=100 µm. Related to Supplementary Figure S2D(top) and S2E(bottom).

File Name: Supplementary Movie 4

Description: Top: Two representative time-lapse movies of *MS2-SLAMR:MCP-RFP* reporter mRNA granules (red) moving in DIV15 hippocampal neurons transfected with Scr-shRNA (GFP). Bottom: Two representative time-lapse movies of *MS2-SLAMR:MCP-RFP* reporter mRNA granules (red) moving in DIV15 hippocampal neuron transfected with KIF5C-shRNA (GFP). Playback speed 30x real time. Total dendrite length=100 µm. Related to Supplementary Figures S3A (topmost), S3B (second from top), S3C (second from bottom), and S3D (bottommost).

File Name: Supplementary Movie 5

Description: Top: Representative time-lapse movie of *MS2-SLAMR:MCP-RFP* MCP lacking movement in a dendrite of a DIV19 hippocampal neuron, taken 3 minutes after spine stimulation in a nonresponsive spine. Bottom: Representative time-lapse movie of *MS2-SLAMR:MCP-RFP* MCP showing movement toward the spine in a dendrite of a DIV19 hippocampal neuron, taken 3 minutes after spine stimulation in a responsive spine. Stimulated spines positioned in the middle. Playback speed 30x real time. Total dendrite length=50µm. Related to Supplementary Figures S3H(top) and S3I (bottom).

File Name: Supplementary Movie 6

Description: Representative time-lapse movies of *MS2-SLAMR* (top), *MS2-SLAMRΔ92-289* (middle), *MS2-SLAMRΔ898-1130* (bottom) reporter mRNA granules moving in dendrites of DIV15 hippocampal neurons. Playback speed 30x real time. Total dendrite length 100 µm. Related to Supplementary Figures S6A(top), S6C(middle), and S6E (bottom).

File Name: Supplementary Movie 7

Description: Representative time-lapse movies of *MS2-SLAMR* (top), *MS2-SLAMRΔ92-289* (middle), *MS2-SLAMRΔ898-1130* (bottom) reporter mRNA granules moving in

dendrites of DIV15 hippocampal neurons. Playback speed 30x real time. Total dendrite length 100=µm. Related to Supplementary Figures S6B(top), S6D(middle), and S6F(bottom).

File Name: Supplementary Movie 8

Description: Representative time-movies of *MS2-SLAMR* (top), *MS2-SLAMRΔ92-289* (middle), *MS2-SLAMRΔ898-1130* (bottom) reporter mRNA granules (green) moving and dendritic spines labeled with PSD95-mCherry (red) in dendrites of a DIV15 hippocampal neurons. Playback speed 30x real time. Total dendrite length 100=µm. Related to Supplementary Figures S6G(top), S6I(middle), and S6K(bottom).

File Name: Supplementary Movie 9

Description: Representative time-movies of *MS2-SLAMR* (top), *MS2-SLAMRΔ92-289* (middle), *MS2-SLAMRΔ898-1130* (bottom) reporter mRNA granules (green) moving and dendritic spines labeled with PSD95-mCherry (red) in dendrites of a DIV15 hippocampal neurons. Playback speed 30x real time. Total dendrite length=100 µm. Related to Supplementary Figures S6H(top), S6J(middle), and S6L(bottom).
