# Supplementary material for: Synaptically-targeted long non-coding RNA SLAMR promotes structural plasticity by increasing translation and CaMKII activity
Source: Nat Commun. 2024 Mar 27;15:2694. doi: 10.1038/s41467-024-46972-8 (PMC10973417; doi:10.1038/s41467-024-46972-8)
Supplement: Supplementary file 14 — Reporting Summary [file 41467_2024_46972_MOESM14_ESM.pdf]

nature portfolio

Corresponding author(s): Sathyanarayanan Puthanveettil

Last updated by author(s): Feb 19, 2024

Reporting Summary

Nature Portfolio wishes to improve the reproducibility of the work that we publish. This form provides structure for consistency and transparency in reporting. For further information on Nature Portfolio policies, see our Editorial Policies and the Editorial Policy Checklist.

Statistics

For all statistical analyses, confirm that the following items are present in the figure legend, table legend, main text, or Methods section.

n/a	Confirmed	
☐	☒	The exact sample size (*n*) for each experimental group/condition, given as a discrete number and unit of measurement
☐	☒	A statement on whether measurements were taken from distinct samples or whether the same sample was measured repeatedly
☐	☒	The statistical test(s) used AND whether they are one- or two-sided<br>*Only common tests should be described solely by name; describe more complex techniques in the Methods section.*
☒	☐	A description of all covariates tested
☐	☒	A description of any assumptions or corrections, such as tests of normality and adjustment for multiple comparisons
☐	☒	A full description of the statistical parameters including central tendency (e.g. means) or other basic estimates (e.g. regression coefficient) AND variation (e.g. standard deviation) or associated estimates of uncertainty (e.g. confidence intervals)
☐	☒	For null hypothesis testing, the test statistic (e.g. *F*, *t*, *r*) with confidence intervals, effect sizes, degrees of freedom and *P* value noted<br>*Give P values as exact values whenever suitable.*
☒	☐	For Bayesian analysis, information on the choice of priors and Markov chain Monte Carlo settings
☐	☒	For hierarchical and complex designs, identification of the appropriate level for tests and full reporting of outcomes
☐	☒	Estimates of effect sizes (e.g. Cohen's *d*, Pearson's *r*), indicating how they were calculated

Our web collection on statistics for biologists contains articles on many of the points above.

Software and code

Policy information about availability of computer code

Data collection	ImageJ (Fiji), Ethovision XT 8.5, SlideBook6, Olympus Fluoview1000 (64bit), Salmon software. Fluorescence images were quantified using TimeHarp 260 Pico card (PicoQuant, Inc) and custom-built software, FU Mage (Ver 2.0.20) written with #C (https:// github.com/ryoheiyasuda/ FLIMage_public).
Data analysis	GraphPad Prism 8,9 and 10, Microsoft Excel, MATLAB, Tophat2 (v.2.0.9), Cufflinks suite (v.2.1.1), FastX-Toolkit (v.0.013) (Toolkit by Hannon Lab).

For manuscripts utilizing custom algorithms or software that are central to the research but not yet described in published literature, software must be made available to editors and reviewers. We strongly encourage code deposition in a community repository (e.g. GitHub). See the Nature Portfolio guidelines for submitting code & software for further information.

Data

Policy information about availability of data

All manuscripts must include a data availability statement. This statement should provide the following information, where applicable:

- Accession codes, unique identifiers, or web links for publicly available datasets
- A description of any restrictions on data availability
- For clinical datasets or third party data, please ensure that the statement adheres to our policy

RNAseq data related to Figures 1 and 6 were deposited to NCBI Gene Expression Omnibus with the accession numbers GSE214838 and GSE214839 respectively.

Also,LC-MS/MS data included in Figure 6 was deposited to MassIVE with the project number MSV000091477. All relevant data are available from the authors upon reasonable request.

Human research participants

Policy information about studies involving human research participants and Sex and Gender in Research.

Reporting on sex and gender	N/A
Population characteristics	N/A
Recruitment	N/A
Ethics oversight	N/A

Note that full information on the approval of the study protocol must also be provided in the manuscript.

Field-specific reporting

Please select the one below that is the best fit for your research. If you are not sure, read the appropriate sections before making your selection.

- [x] Life sciences
- [ ] Behavioural & social sciences
- [ ] Ecological, evolutionary & environmental sciences

For a reference copy of the document with all sections, see nature.com/documents/nr-reporting-summary-flat.pdf

Life sciences study design

All studies must disclose on these points even when the disclosure is negative.

Sample size	Sample sizes are indicated in the results section and figure legends. In general, no statistical methods were used to determine the sample size, just prior experience and published data (Swarnkar et al. 2021, Bauer et al. 2019, etc.) was used to determine a proper N. However, for experiments that involved the use of animals, this was determined to maximized the volume of information collected, with the optimal statistical result considering the lower number of animals used for each experiment. For example, for in vivo experiments the estimate by power analysis is 5-16 samples. In the power analysis tests, a variance= 0.8-1.05 was determined; a treatment effect (µ-µOJ= 1.2-2; and a statistical power of greater than/equal to 0.95. The statistical test used was Ruth Lenth's.
Data exclusions	For behavioral studies that implicate specific manipulations in hippocampus, animals that did not show bilateral infusions of the GapmR were excluded from the studies, as these animals would not have a reduction in SLAMR in the CA1. For in vitro studies, any data from unhealthy cells or cultures were excluded as described in the methods, as we would not be able to distinguish between results cause by our treatment or results caused due to pathways activated in a dying cell. ROUT's (Q=0.5%, to identify any number of outliers) or GRUBB's (alpha=0.05 to identify single outliers) tests were used to identify possible outliers.
Replication	In vitro experiments that involve cell cultures (ex. morphological studies) or tissue analyses (ex. Pull down experiments) were repeated at least in 3 independent experiments to confirm the results. Only experiments where all attempts at replication were successful were included in this paper. Other experiments like those that implicate the use of an important number of animals (like behavioral studies) were not replicate for ethical reasons.
Randomization	For in vivo experiments and tissue collection, mice were randomly assigned to each experimental group. We tried to distribute littermates, evenly between groups, where numbers allowed, to ensure the litter did not bias results. For imaging experiments we alternated the order of imaging control and treatment plates between experimental days to ensure the timing from transfection/transduction to imaging did not bias results.
Blinding	Researchers performing behavioral experiments were blinded to which treatment group the mice were in. For MS2-SLAMR analysis following single spine stimulation (Figure 3N,M) the researcher analyzing the MS2-SLAMR Puncta was blinded to the responsiveness of the spines. For all other analyses results were unambiguous and a researcher's unconscious bias would not have an effect on the results.

Reporting for specific materials, systems and methods

We require information from authors about some types of materials, experimental systems and methods used in many studies. Here, indicate whether each material, system or method listed is relevant to your study. If you are not sure if a list item applies to your research, read the appropriate section before selecting a response.

Materials & experimental systems

n/a	Involved in the study
☐	☒ Antibodies
☐	☒ Eukaryotic cell lines
☒	☐ Palaeontology and archaeology
☐	☒ Animals and other organisms
☒	☐ Clinical data
☒	☐ Dual use research of concern

Methods

n/a	Involved in the study
☒	☐ ChIP-seq
☒	☐ Flow cytometry
☒	☐ MRI-based neuroimaging

Antibodies

Antibodies used

Puromycin (EMD Millipore, MABE342, Lot:3166081, clone 4G11,mouse), alpha-Tubulin (Invitrogen, PA1-38814, Lot:VI3090432, Rabbit), GFP (Novus Biologicals, NB100-1614, Lot:917979, chicken), Map2 (Synaptic systems, 188 004, Guinea Pig), CamKIIα (invitrogen, MA1-048, Lot:UH288077, Mouse), p-CaMKII-T286 (Cell Signaling Technology, #12716S, Lot:5, D21E4, Rabbit), Anti-DIG fab fragment antibody (Roche, 11207733910, Lot:35698000) , beta-actin (Abcam, Ab8227, Lot:LR288171-1, Rabbit), synaptophysin (Abcam, ab32594, Lot: GR198446-4, Rabbit), Vimentin (AbCam, ab137321, Lot:GR3356521-4, Rabbit), eIF2alpha (Cell Signaling Technology,mAb #5324, Lot:3, D7D3, Rabbit), Gapdh (Santa Cruz Biotechnology, sc-32233, Lot:A2319, 6C5, mouse), eIF3G (Novus Biologicals, NB100-93298, Lot:A1, Rabbit), GluR2 (EMD Millipore, MABN71, Lot:3460365, clone L21/32, mouse), P70S6K (Cell Signaling Technology, #9202S, Lot:3, Rabbit), Anti-rabbit IgG HRP-linked (Cell Signaling Technology, #7074S, Lot:31, Goat), Anti-mouse IgG HRP-linked (Cell Signaling Technology, #7076S, Lot:35, Horse), Anti-rabbit Alexa Fluor647 (Invitrogen, A31573, Lot:169297, donkey), Anti-mouse Alexa Fluor546 (invitrogen, A10036, Lot:771559, donkey).

Validation

For all Cell Signaling Technology Antibodies (from the company website): This product is thoroughly validated with CST primary antibodies and will work optimally with the CST western immunoblotting protocol, ensuring accurate and reproducible results. Puromycin: Evaluated by Western Blotting in HEK293 cell lysates treated with Puromycin and Cyclohexamide, or with Puromycin only. Demonstrated to react with Human test sample, preincubated with Puromycin. Predicted to react with all species when test sample is incubated with Puromycin. alpha-Tubulin (Invitrogen cites Mao et al., 2023 PMID: 34502373). Map2(Synaptic systems cites Hauser et al., 2022PMID: 35550065 and others). CamKIIα (From the company website: MA1-048 detects CaM kinase II from rat and mouse tissues. This antibody has been shown to detect both the phosphorylated and non-phosphorylated alpha subunit of CaM kinase II only. MA1-048 does not detect the ~60 kDa beta subunit in either phosphorylation state. MA1-048 has been successfully used in Western blot, immunohistochemistry, immunofluorescence and immunoprecipitation procedures. By Western blot, this antibody detects an ~50 kDa protein representing the alpha subunit of CaM kinase II). Anti-DIG (From manufacturer: The polyclonal antibody from sheep is specific to digoxigenin and digoxin and shows no cross-reactivity with other steroids, such as human estrogens and androgens.). beta-actin (from Abcam: Positive control WB: A431, HeLa, Jurkat, HEK-293, NIH/3T3, MDCK, EBTr, SL-29, CHO and PC-12 whole cell lysate. Rat liver tissue lysate. HeLa nuclear lysate. Fish and rabbit liver. Xenopus laevis embryo. ICC: SV40LT-SMC and NIH/3T3 cells. IHC-P: Rat small intestine tissue. Human colon tissue.). Synaptophysin (From Abcam: Validated with WB: Mouse and rat brain tissue lysates. ICC/IF: rat hippocampal neurons). Vimentin (Abcam cited Juntong Wang et. al., 2022 PMID32776110). Gapdh (from santa cruz biotechnology: Validated with GAPDH Antibody (6C5): sc-32233. Western blot analysis of GAPDH expression in Hep G2, A549, and Raji whole cell lysates.). eIF3G (Mouse reactivity reported in scientific literature (PMID: 25349259)). GluR2 (From Manufacturer: Detect GluR2 using this Anti-GluR2 Antibody, clone L21/32 validated for use in western blot, immunohistochemistry). Anti-Rabbit Alexa Fluor647 (Specificity of secondary antibody was demonstrated by specific detection of the target immunoglobulin. Antibody specificity was demonstrated by specific detection of Rabbit IgG. A band at ~50 kDa corresponding to Rabbit IgG Heavy Chain was observed in Rabbit IgG but not in other species using Donkey anti-Rabbit IgG (H+L) Highly Cross-Adsorbed Secondary Antibody, Alexa Fluor™ 647 (Product # A-31573) in Western Blot.). Anti-Mouse Alexa Fluor546 (These donkey anti-mouse IgG whole secondary antibodies have been affinity-purified and show minimum cross-reactivity to bovine, chicken, goat, guinea pig, hamster, horse, human, rabbit, rat, and sheep serum proteins. Cross-adsorption or pre-adsorption is a purification step to increase specificity of the antibody resulting in higher sensitivity and less background staining. The secondary antibody solution is passed through a column matrix containing immobilized serum proteins from potentially cross-reactive species. Only the nonspecific-binding secondary antibodies are captured in the column, and the highly specific secondaries flow through. The benefits of this extra step are apparent in multiplexing/multicolor-staining experiments (e.g., flow cytometry) where there is potential cross-reactivity with other primary antibodies or in tissue/cell fluorescent staining experiments where there may be the presence of endogenous immunoglobulins.).

Eukaryotic cell lines

Policy information about cell lines and Sex and Gender in Research

Cell line source(s)

Human embryonic kidney (HEK)293T cells (CRL-11268), used to generate lentivirus, came from from the American Type Culture Collection (ATCC; Manassas, VA, USA).

Authentication

HEK293T cells were not authenticated.

Mycoplasma contamination

Cell lines were not tested for mycoplasma contamination.

Commonly misidentified lines
(See ICLAC register)

No commonly misidentified cell lines were used.

Animals and other research organisms

Policy information about studies involving animals; ARRIVE guidelines recommended for reporting animal research, and Sex and Gender in Research

Laboratory animals	This study used male 8-10 week old C57BL6 mice from Jackson Laboratories; mixed-sex CD1 E16/17 mouse pups from Charles River, and mixed-sex sprague dawley rat PO pups. Mice at the Wertheim UF Scripps Institute were kept at an ambient temperature of 22C and 28 - 72 % humidity. Rats at the Max Planck Florida Institute for Neuroscience were kept at an ambient temperature of 22C and 35 - 60 % humidity. Both facilities keep a 12hr light/dark cycle.
Wild animals	No wild animals were used in this study.
Reporting on sex	With the aim of reduce the total number of animals used in this study and facilitate the handling and housing conditions, only male mice were used in this study.
Field-collected samples	No field-collected were used in this study.
Ethics oversight	Housing and experimental procedures were approved and supervised by the Institutional Animal Care and Use Committee of the Herbert Wertheim UF Scripps Institute for Biomedical Innovation & Technology and Max Planck Florida Institute for Neuroscience.

Note that full information on the approval of the study protocol must also be provided in the manuscript.
