# Supplementary material for: Synaptically-targeted long non-coding RNA SLAMR promotes structural plasticity by increasing translation and CaMKII activity
Source: Nat Commun. 2024 Mar 27;15:2694. doi: 10.1038/s41467-024-46972-8 (PMC10973417; doi:10.1038/s41467-024-46972-8)
Supplement: Supplementary file 15 — Source Data [file 41467_2024_46972_MOESM15_ESM.zip › Espadas et al. 2024 Source Files/Espadas et al. 2024 Western Blots/Figure 5L eIFg eIF2a P70S6K.pptx]

## Slide 1
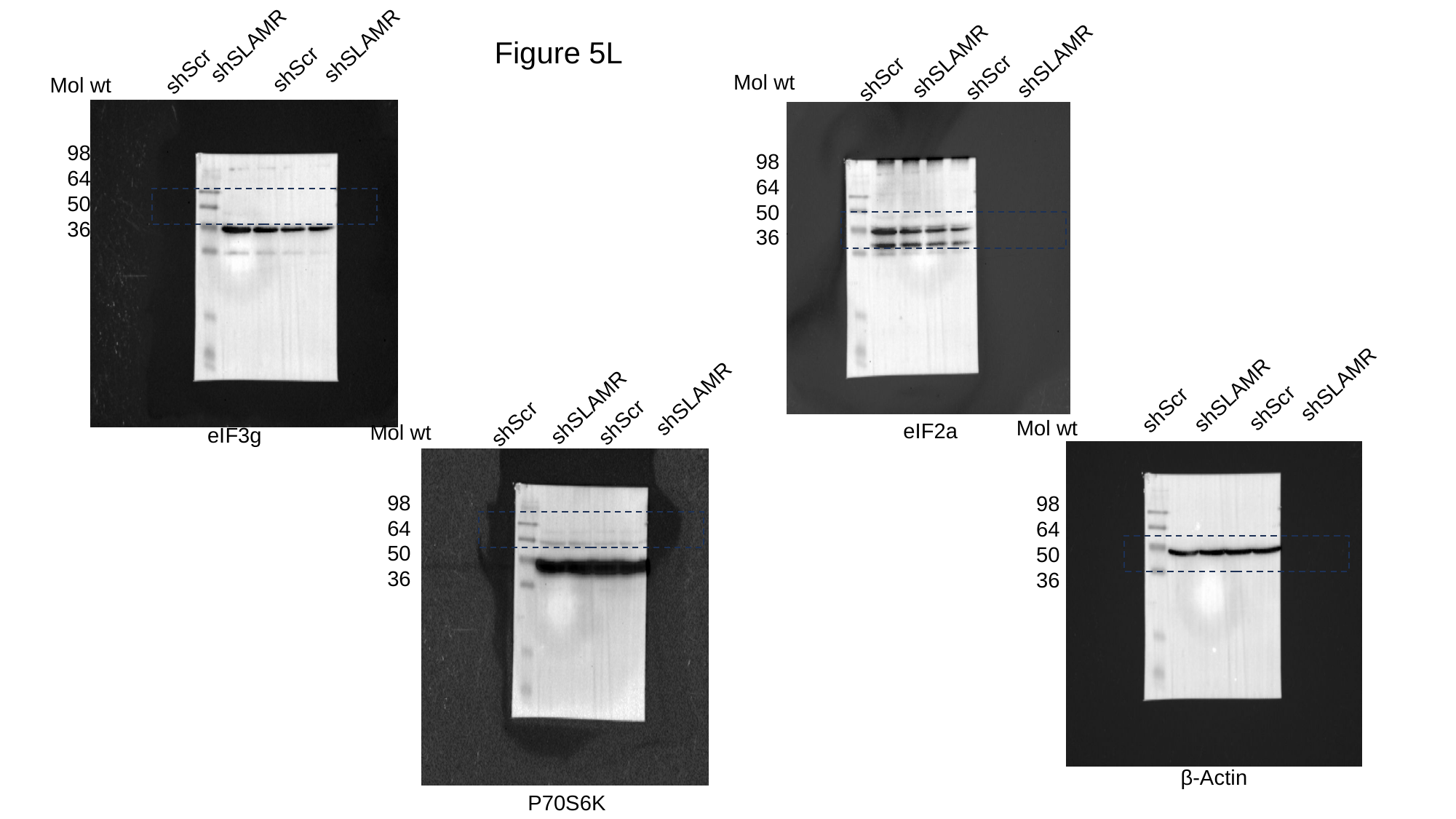

shSLAMR
shSLAMR
Figure 5L
shSLAMR
shSLAMR
shScr
shScr
shScr
shScr
Mol wt
Mol wt
98
64
50
36
98
64
50
36
shSLAMR
shSLAMR
shSLAMR
shSLAMR
shScr
shScr
shScr
shScr
Mol wt
eIF2a
Mol wt
eIF3g
98
64
50
36
98
64
50
36
β-Actin
P70S6K
