# Supplementary material for: Synaptically-targeted long non-coding RNA SLAMR promotes structural plasticity by increasing translation and CaMKII activity
Source: Nat Commun. 2024 Mar 27;15:2694. doi: 10.1038/s41467-024-46972-8 (PMC10973417; doi:10.1038/s41467-024-46972-8)
Supplement: Supplementary file 15 — Source Data [file 41467_2024_46972_MOESM15_ESM.zip › Espadas et al. 2024 Source Files/Espadas et al. 2024 Western Blots/Figure 6H pCaMKII and CaMKII.pptx]

## Slide 1
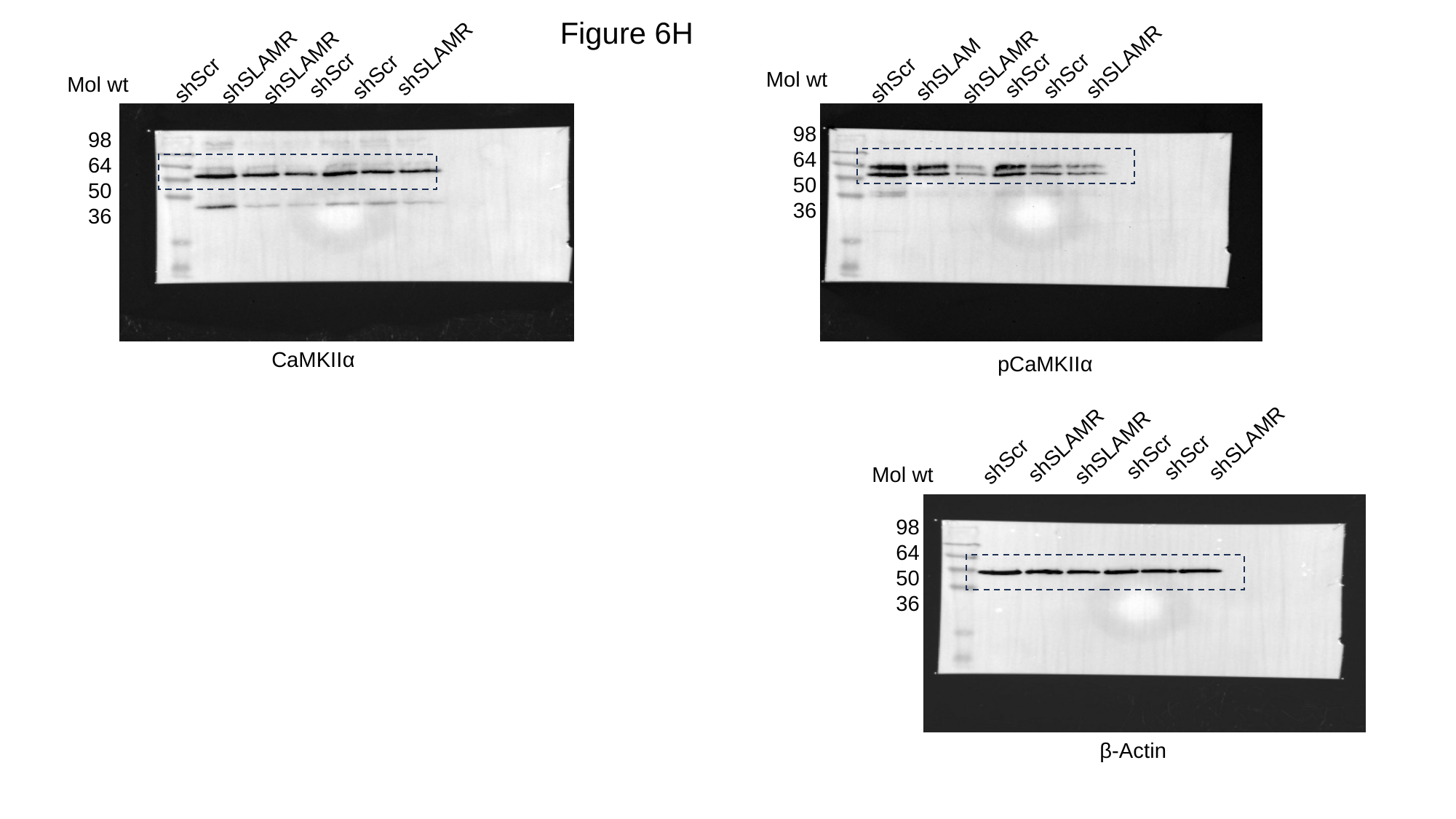

Figure 6H
shSLAMR
shSLAMR
shSLAMR
shSLAMR
shSLAM
shSLAMR
shScr
shScr
shScr
shScr
shScr
shScr
Mol wt
Mol wt
98
64
50
36
98
64
50
36
CaMKIIα
pCaMKIIα
shSLAMR
shSLAMR
shSLAMR
shScr
shScr
shScr
Mol wt
98
64
50
36
β-Actin
