# Supplementary material for: Synaptically-targeted long non-coding RNA SLAMR promotes structural plasticity by increasing translation and CaMKII activity
Source: Nat Commun. 2024 Mar 27;15:2694. doi: 10.1038/s41467-024-46972-8 (PMC10973417; doi:10.1038/s41467-024-46972-8)
Supplement: Supplementary file 15 — Source Data [file 41467_2024_46972_MOESM15_ESM.zip › Espadas et al. 2024 Source Files/Espadas et al. 2024 Western Blots/Supplementary Figure S4C Validation of Synatoneurosome Isolation.pptx]

## Slide 1
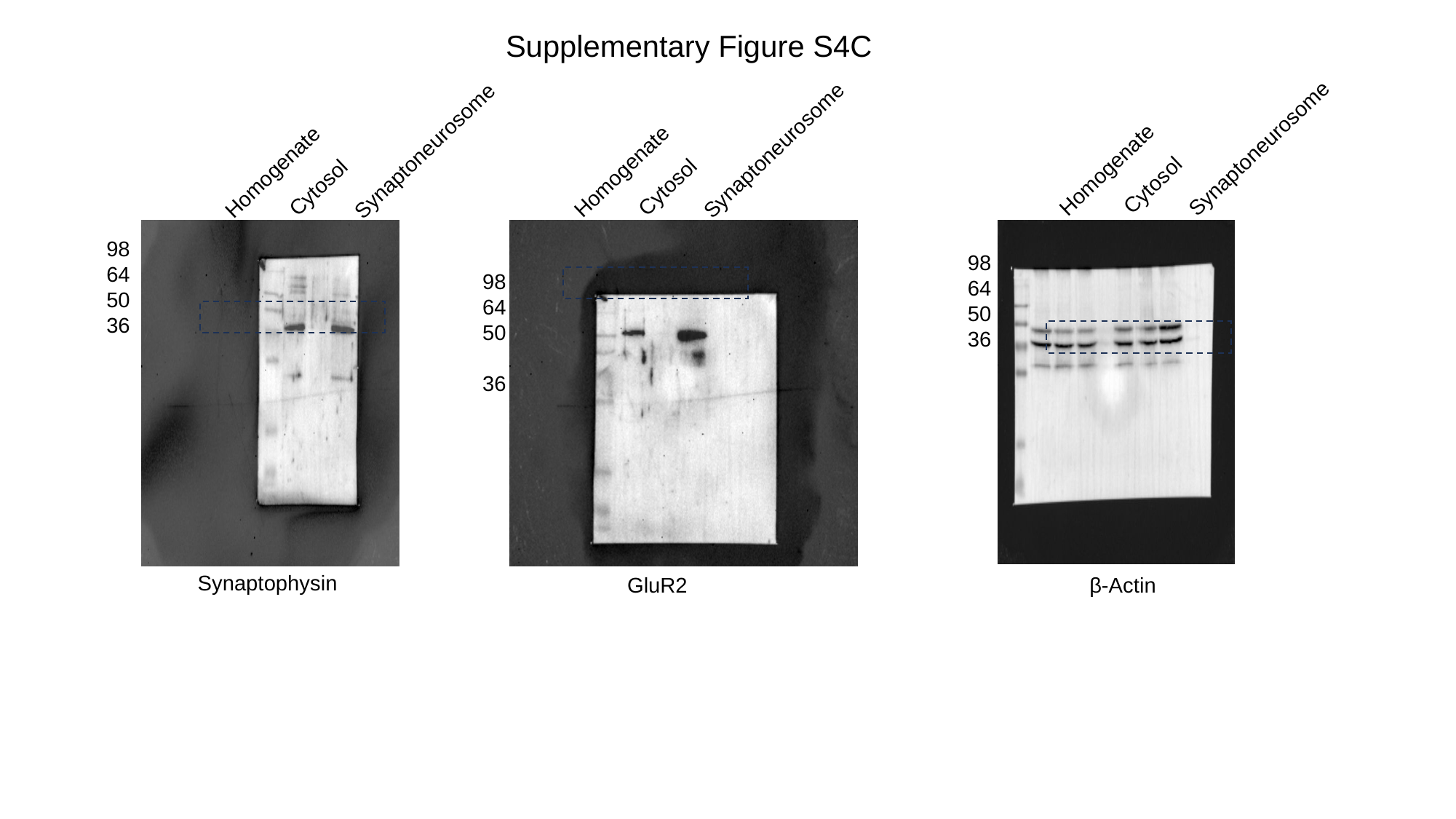

Supplementary Figure S4C
Synaptoneurosome
Synaptoneurosome
Synaptoneurosome
Homogenate
Homogenate
Homogenate
Cytosol
Cytosol
Cytosol
98
64
50
36
98
64
50
36
98
64
50
36
Synaptophysin
GluR2
β-Actin
