# Supplementary figures and images for: Synaptically-targeted long non-coding RNA SLAMR promotes structural plasticity by increasing translation and CaMKII activity
Source: Nat Commun. 2024 Mar 27;15:2694. doi: 10.1038/s41467-024-46972-8 (PMC10973417; doi:10.1038/s41467-024-46972-8)

## Slide 1
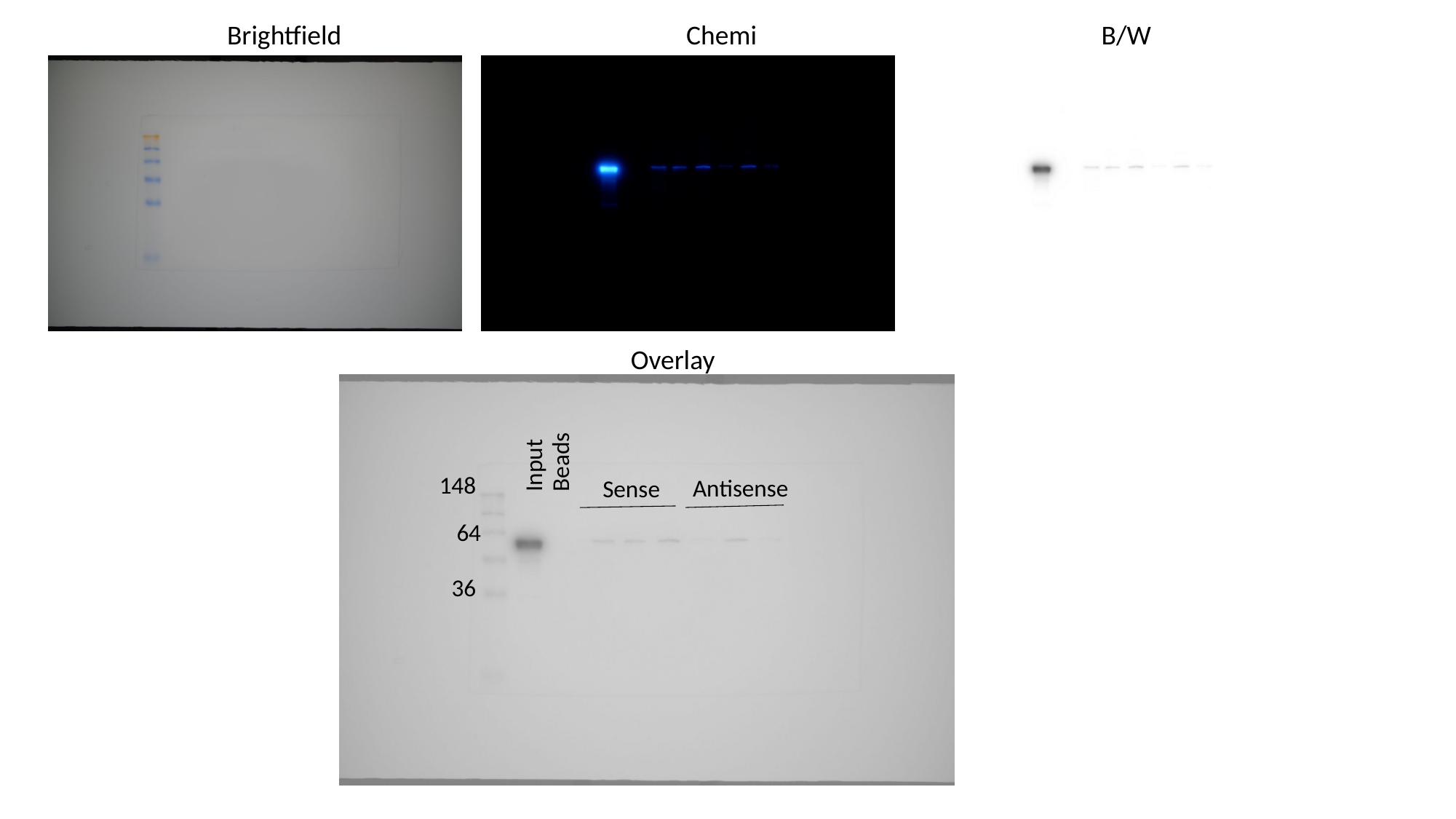

Brightfield
Chemi
B/W
Overlay
Beads
Input
148
Antisense
Sense
64
36

Supplement: Supplementary file 15 — Source Data [file 41467_2024_46972_MOESM15_ESM.zip › Espadas et al. 2024 Source Files/Espadas et al. 2024 Western Blots/Figure 6F CamKII pull down uncropped.pptx]

## Slide 1
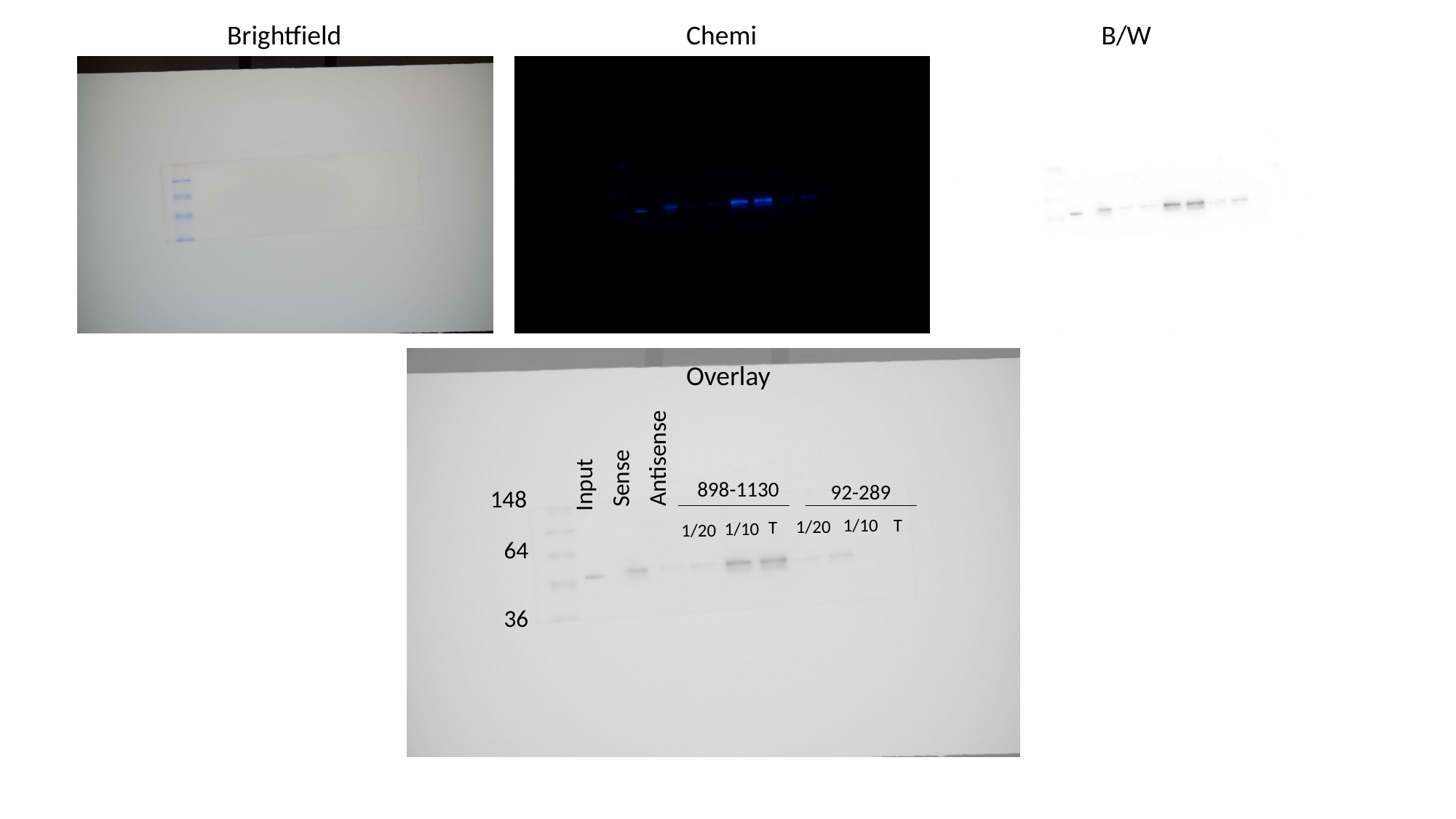

Brightfield
Chemi
B/W
Overlay
Antisense
Sense
Input
898-1130
92-289
148
1/10
T
1/20
T
1/10
1/20
64
36

Supplement: Supplementary file 15 — Source Data [file 41467_2024_46972_MOESM15_ESM.zip › Espadas et al. 2024 Source Files/Espadas et al. 2024 Western Blots/Figure 8D Vimentin protected fragments uncropped.pptx]

## Slide 1
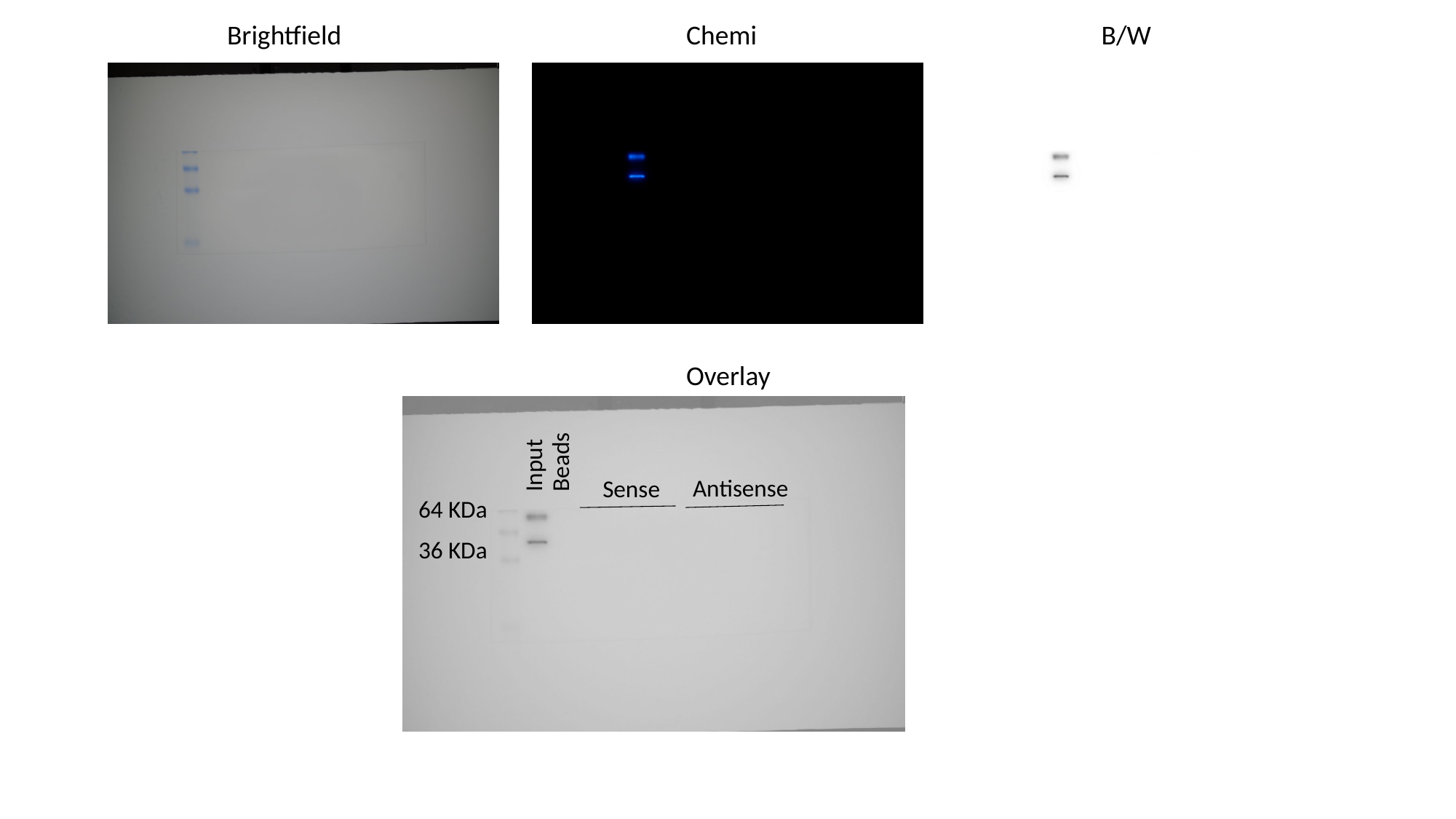

Brightfield
Chemi
B/W
Overlay
Beads
Input
Antisense
Sense
64 KDa
36 KDa

Supplement: Supplementary file 15 — Source Data [file 41467_2024_46972_MOESM15_ESM.zip › Espadas et al. 2024 Source Files/Espadas et al. 2024 Western Blots/Figure 6F Gapdh (CamKII) pull down uncropped.pptx]

## Slide 1
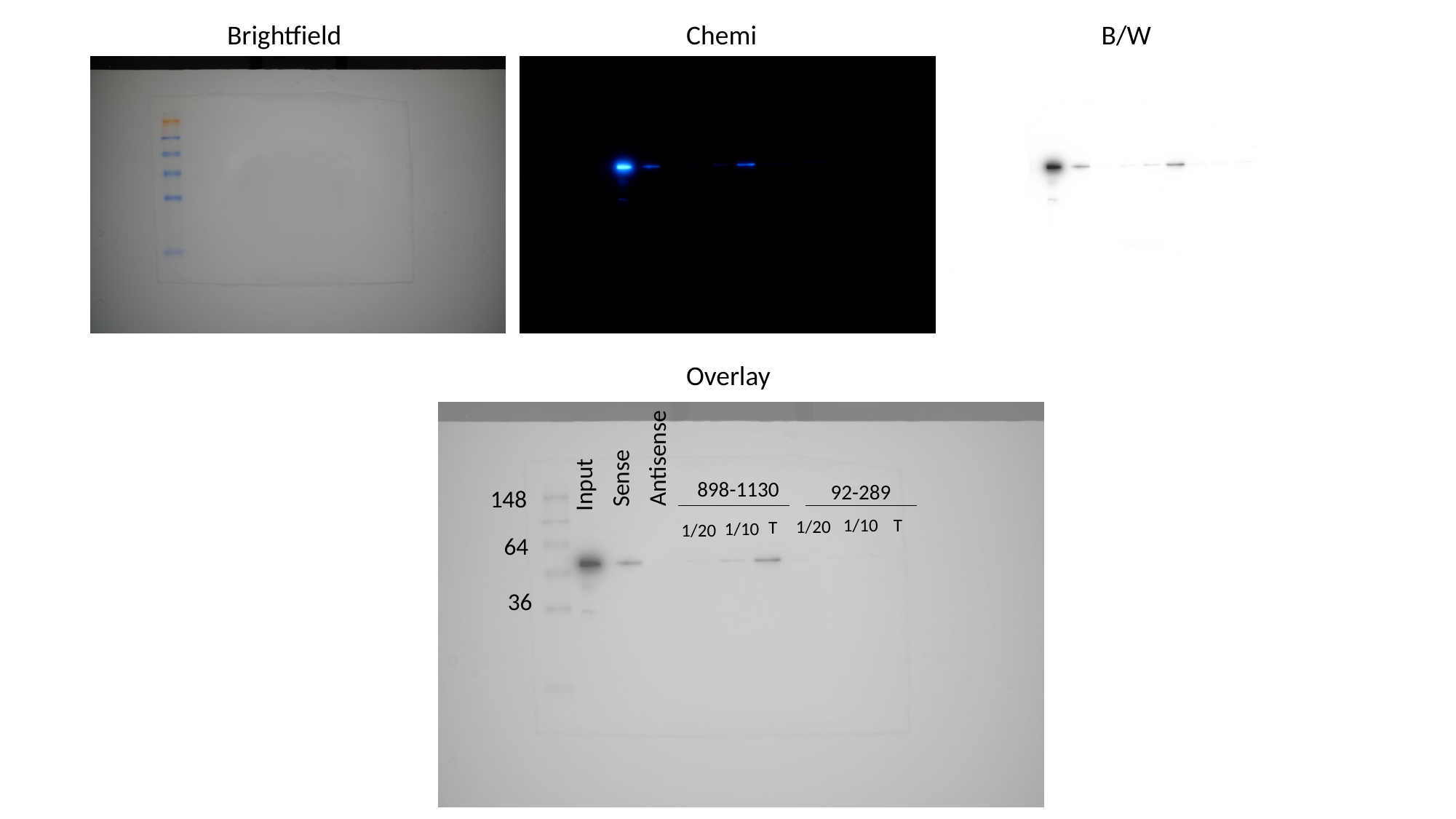

Brightfield
Chemi
B/W
Overlay
Antisense
Sense
Input
898-1130
92-289
148
1/10
T
1/20
T
1/10
1/20
64
36

Supplement: Supplementary file 15 — Source Data [file 41467_2024_46972_MOESM15_ESM.zip › Espadas et al. 2024 Source Files/Espadas et al. 2024 Western Blots/Figure 8D CaMKIIa protected fragments uncropped.pptx]

## Slide 1
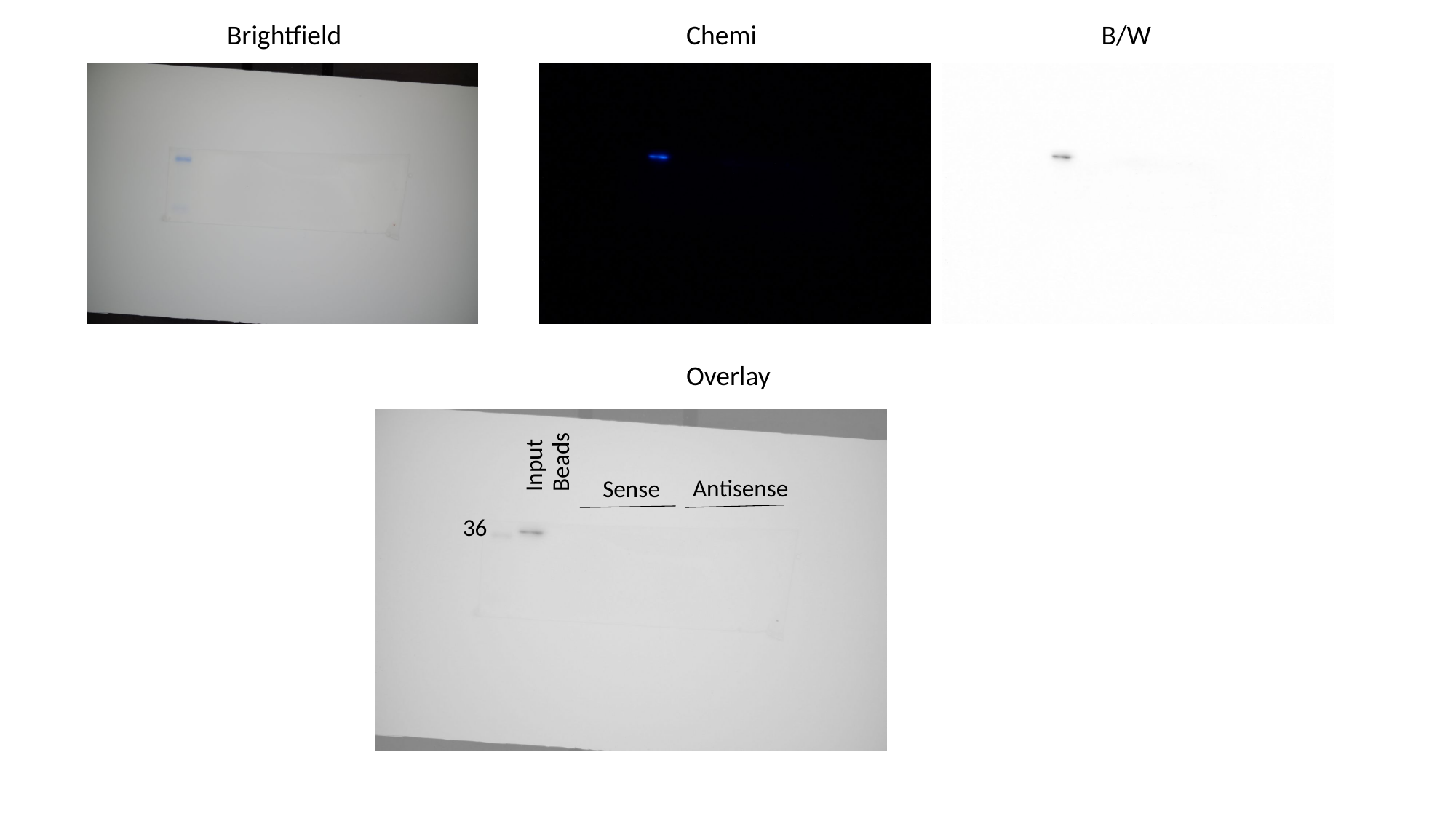

Brightfield
Chemi
B/W
Overlay
Beads
Input
Antisense
Sense
36

Supplement: Supplementary file 15 — Source Data [file 41467_2024_46972_MOESM15_ESM.zip › Espadas et al. 2024 Source Files/Espadas et al. 2024 Western Blots/Figure 6J Gapdh (Vimentin) pull down uncropped.pptx]

## Slide 1
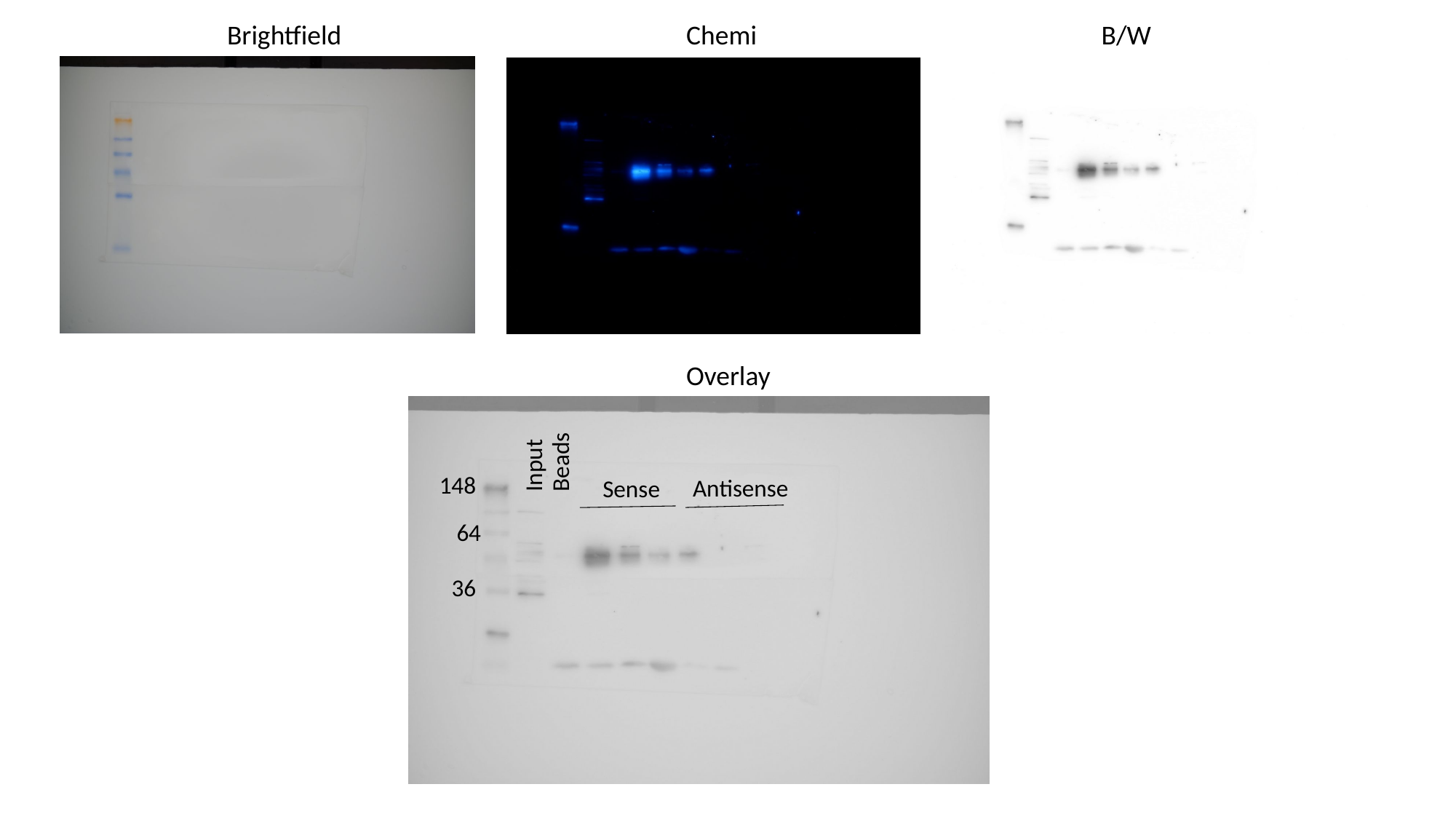

Brightfield
Chemi
B/W
Overlay
Beads
Input
148
Antisense
Sense
64
36

Supplement: Supplementary file 15 — Source Data [file 41467_2024_46972_MOESM15_ESM.zip › Espadas et al. 2024 Source Files/Espadas et al. 2024 Western Blots/Figure 6J Vimentin pull down uncropped.pptx]

## Slide 1
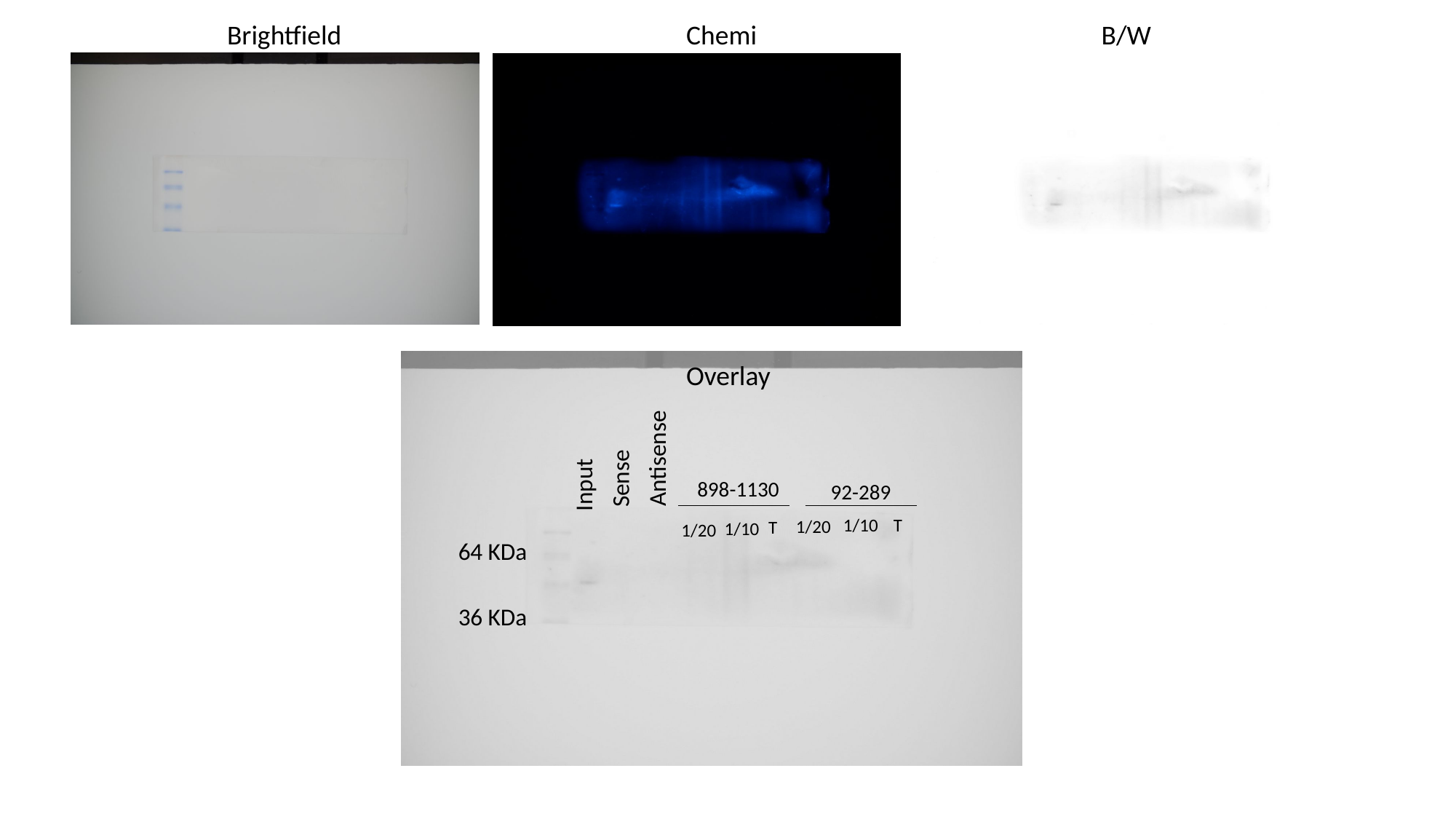

Brightfield
Chemi
B/W
Overlay
Antisense
Sense
Input
898-1130
92-289
1/10
T
1/20
T
1/10
1/20
64 KDa
36 KDa

Supplement: Supplementary file 15 — Source Data [file 41467_2024_46972_MOESM15_ESM.zip › Espadas et al. 2024 Source Files/Espadas et al. 2024 Western Blots/Figure 8D Actin protected fragments uncropped.pptx]

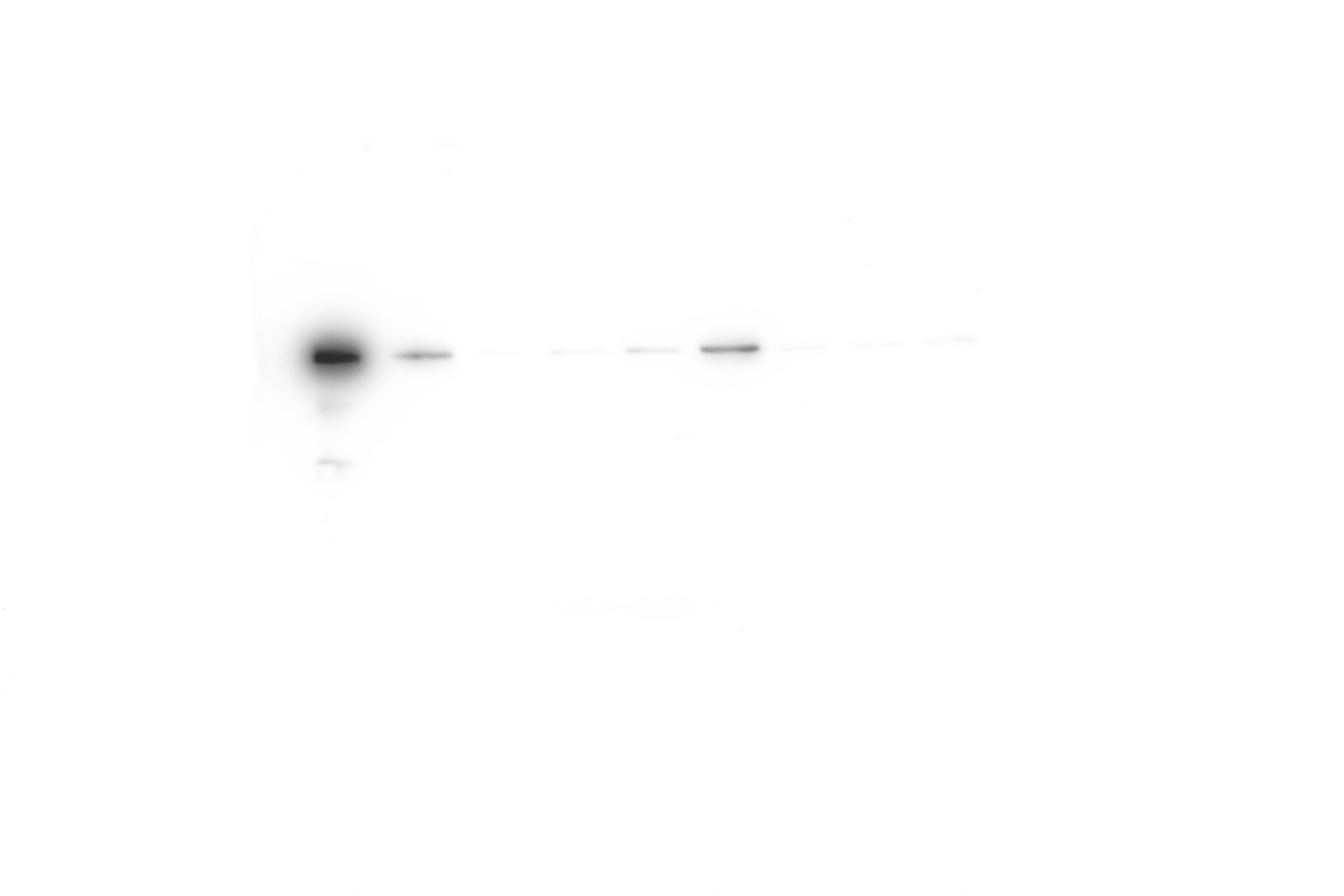

Supplement: Supplementary file 15 — Source Data [file 41467_2024_46972_MOESM15_ESM.zip › Espadas et al. 2024 Source Files/Espadas et al. 2024 Western Blots/TIF files of Western Blots/Figure 8D CamKIIa protected fragments uncropped.jpg]

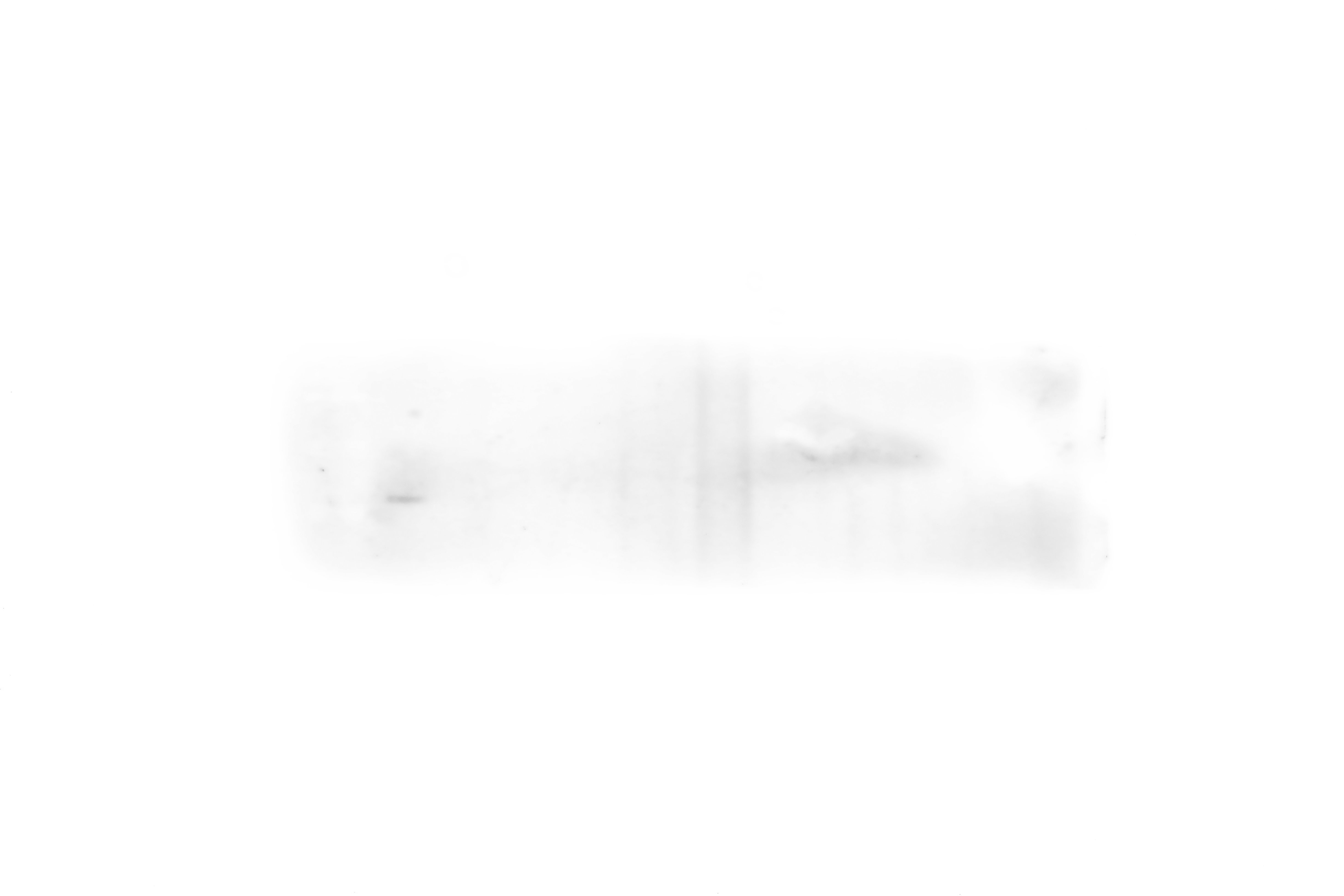

Supplement: Supplementary file 15 — Source Data [file 41467_2024_46972_MOESM15_ESM.zip › Espadas et al. 2024 Source Files/Espadas et al. 2024 Western Blots/TIF files of Western Blots/Figure 8D actin protected fragments.jpg]

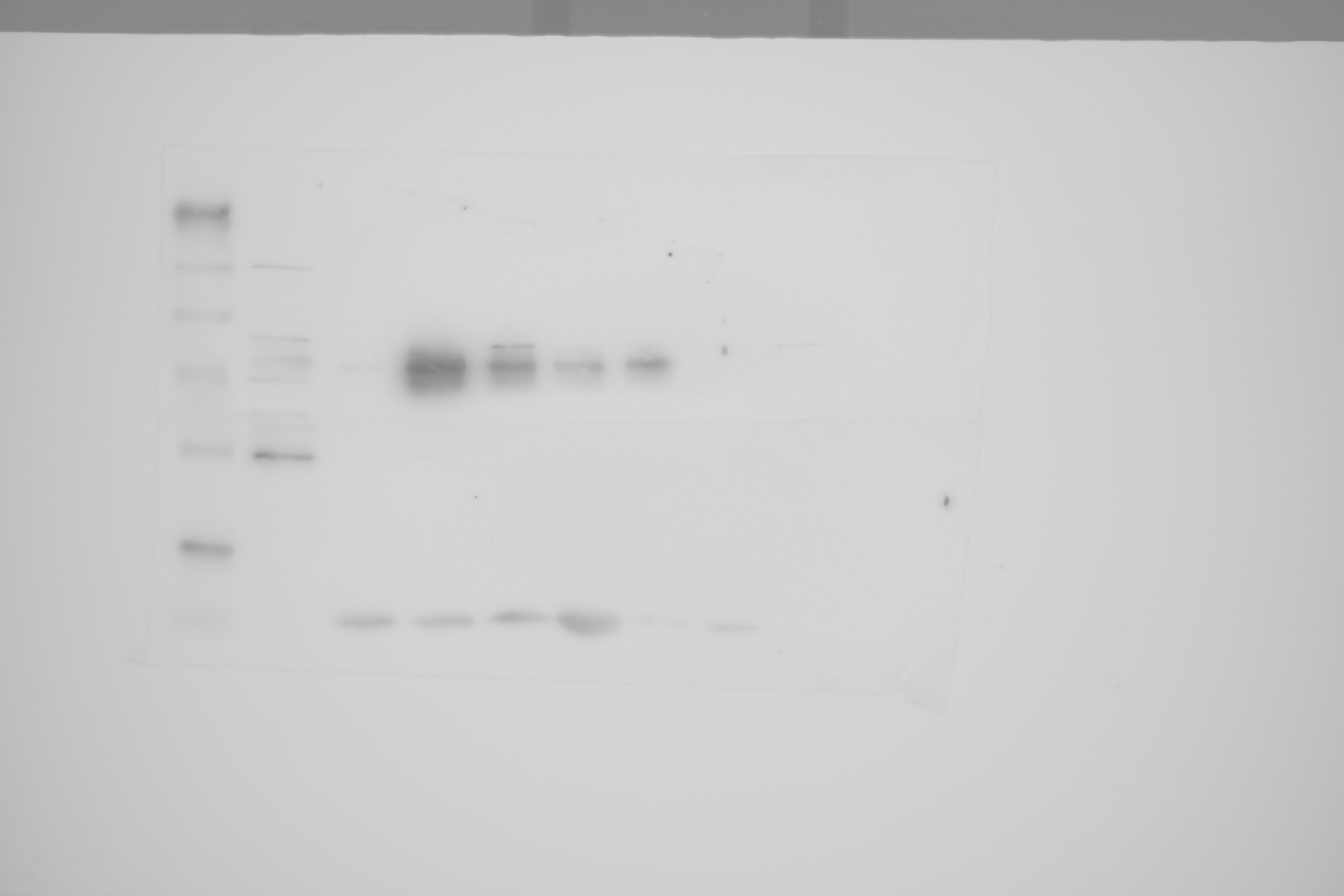

Supplement: Supplementary file 15 — Source Data [file 41467_2024_46972_MOESM15_ESM.zip › Espadas et al. 2024 Source Files/Espadas et al. 2024 Western Blots/TIF files of Western Blots/Figure 6J overlay vimentin pull down.jpg]

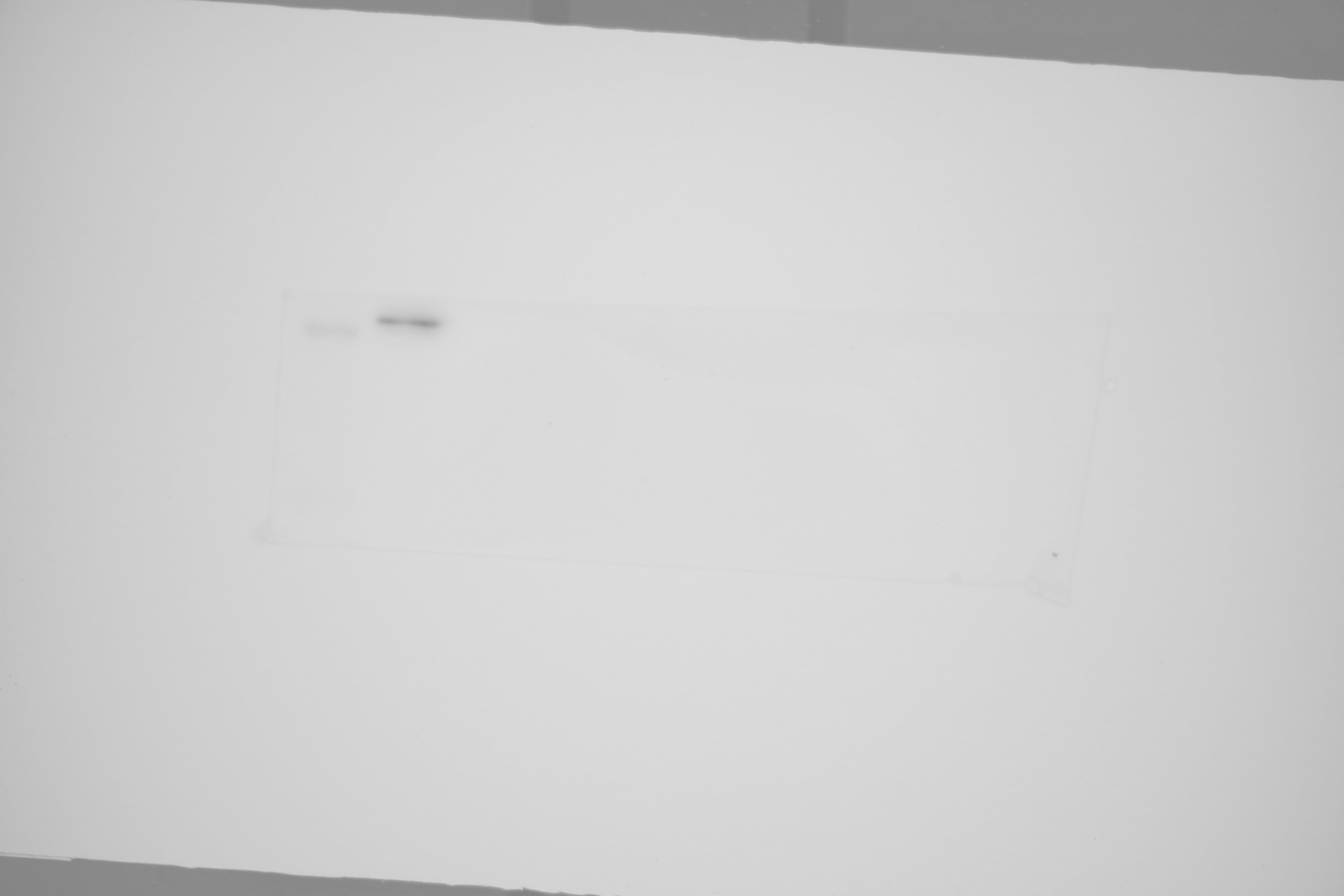

Supplement: Supplementary file 15 — Source Data [file 41467_2024_46972_MOESM15_ESM.zip › Espadas et al. 2024 Source Files/Espadas et al. 2024 Western Blots/TIF files of Western Blots/Figure 6J Overlay Gapdh (vimentin) pull down.jpg]

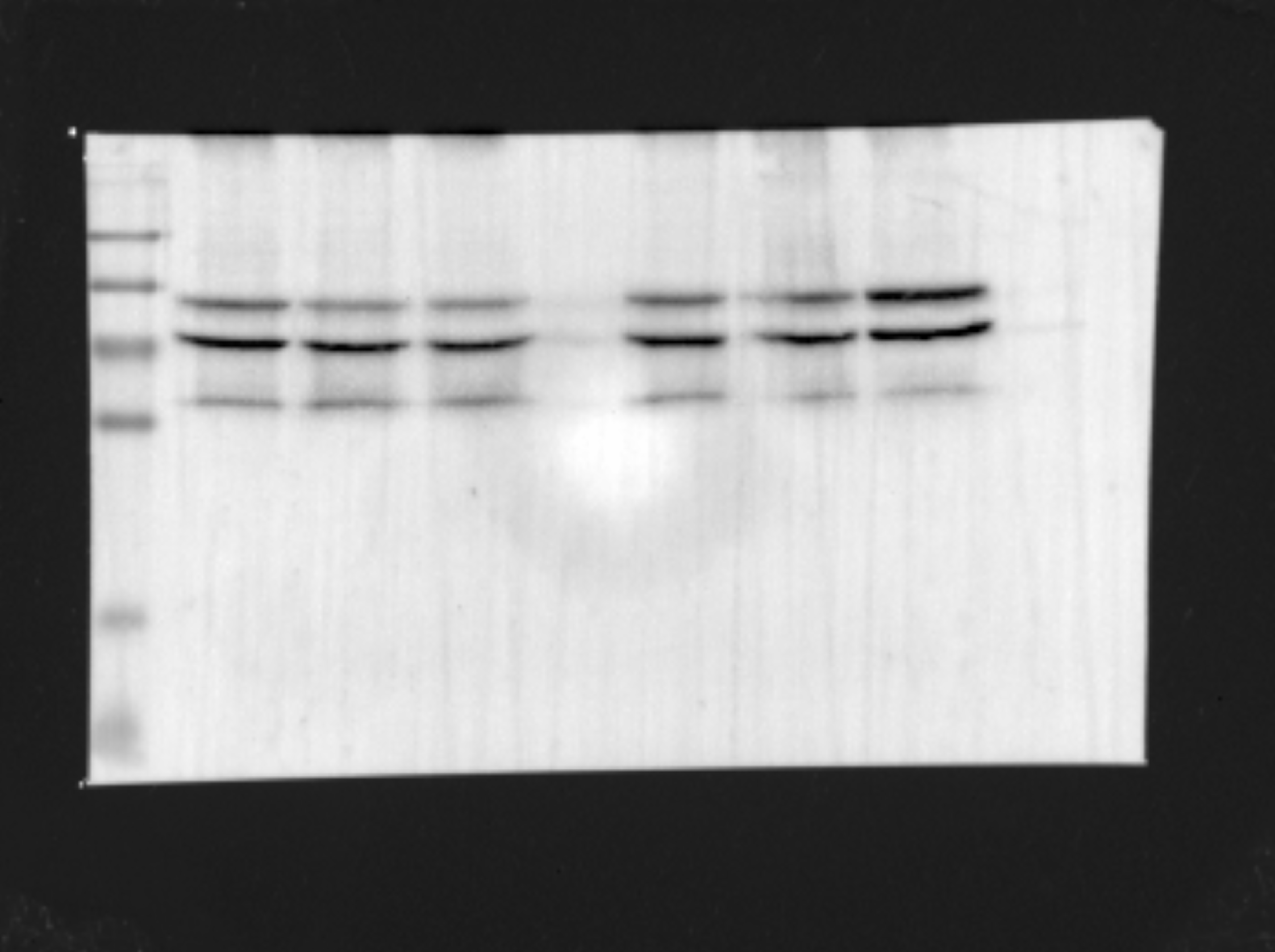

Supplement: Supplementary file 15 — Source Data [file 41467_2024_46972_MOESM15_ESM.zip › Espadas et al. 2024 Source Files/Espadas et al. 2024 Western Blots/TIF files of Western Blots/Figure S4C b-actin.tif]

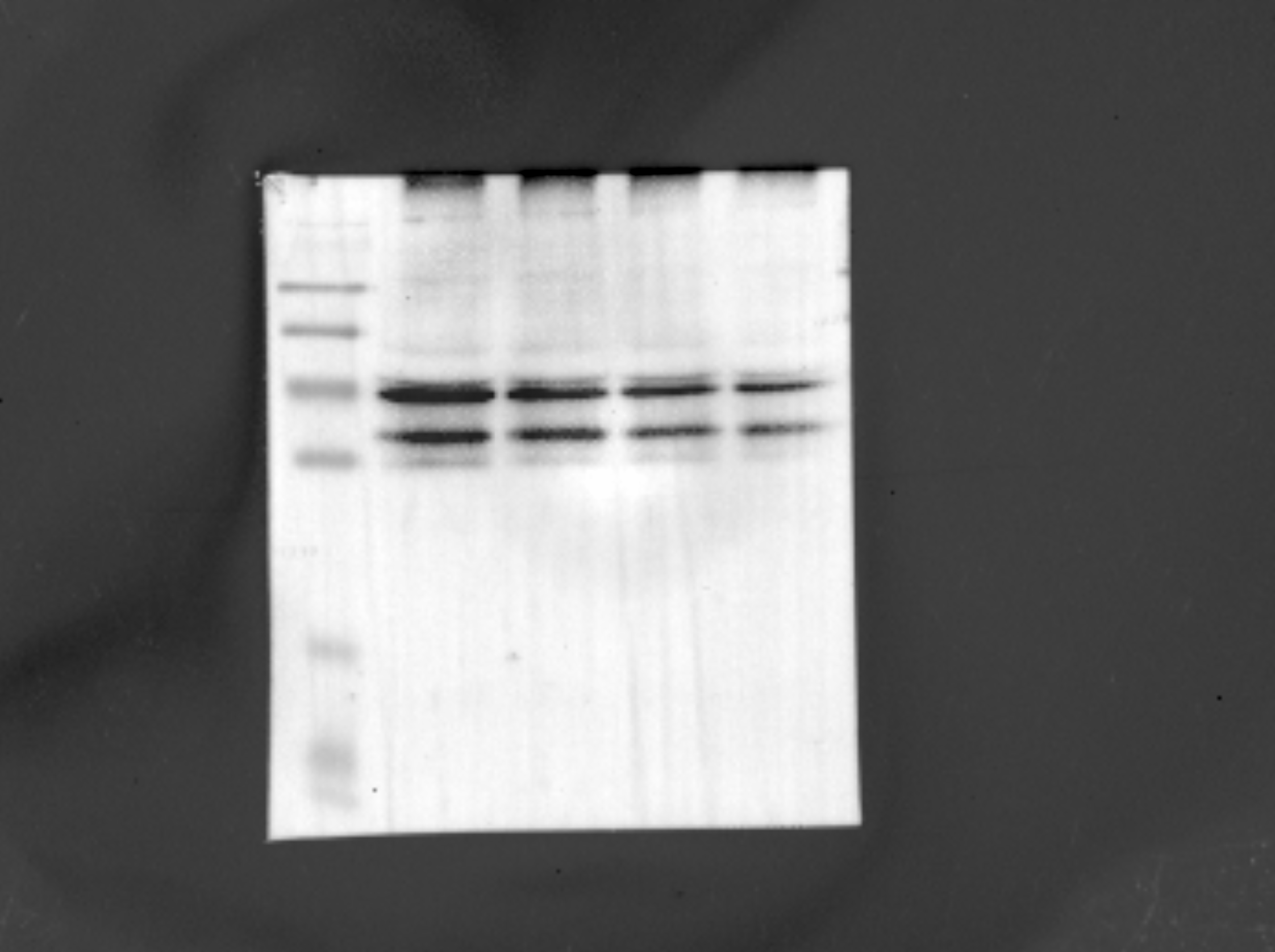

Supplement: Supplementary file 15 — Source Data [file 41467_2024_46972_MOESM15_ESM.zip › Espadas et al. 2024 Source Files/Espadas et al. 2024 Western Blots/TIF files of Western Blots/Figure 5L eif2a.tif]

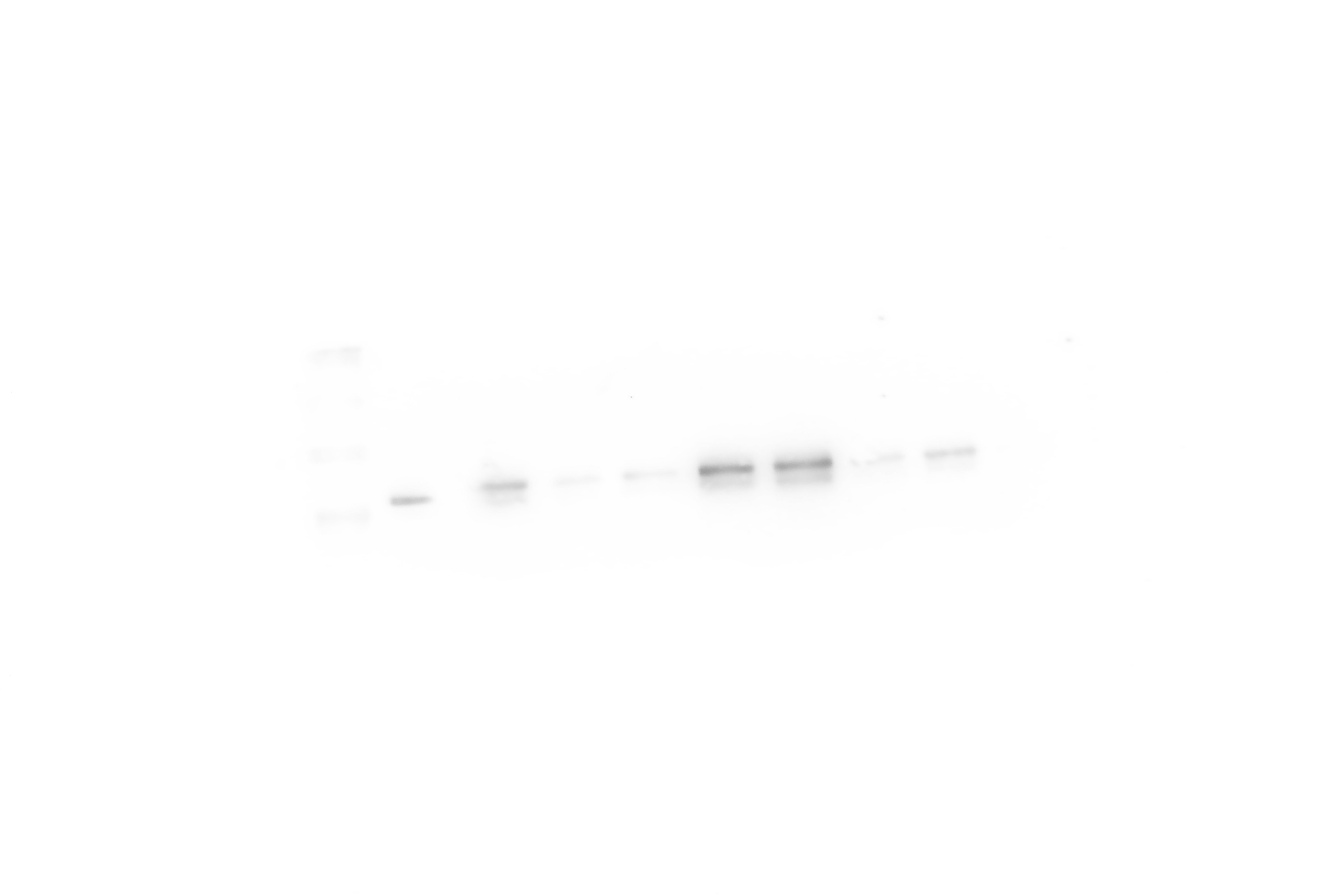

Supplement: Supplementary file 15 — Source Data [file 41467_2024_46972_MOESM15_ESM.zip › Espadas et al. 2024 Source Files/Espadas et al. 2024 Western Blots/TIF files of Western Blots/Figure 8D Vimentin protected fragments.jpg]

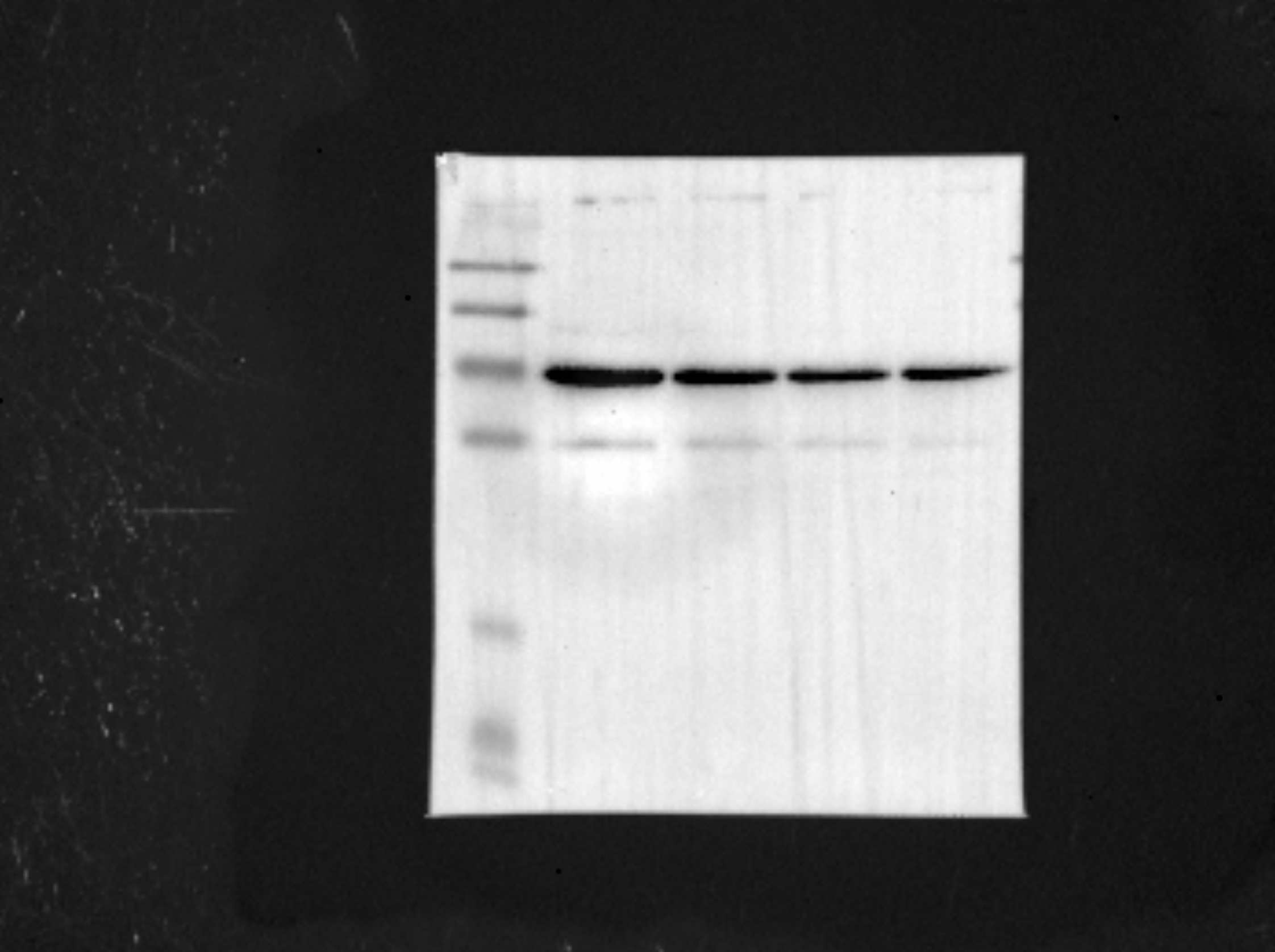

Supplement: Supplementary file 15 — Source Data [file 41467_2024_46972_MOESM15_ESM.zip › Espadas et al. 2024 Source Files/Espadas et al. 2024 Western Blots/TIF files of Western Blots/Figure 5L eif3g.tif]

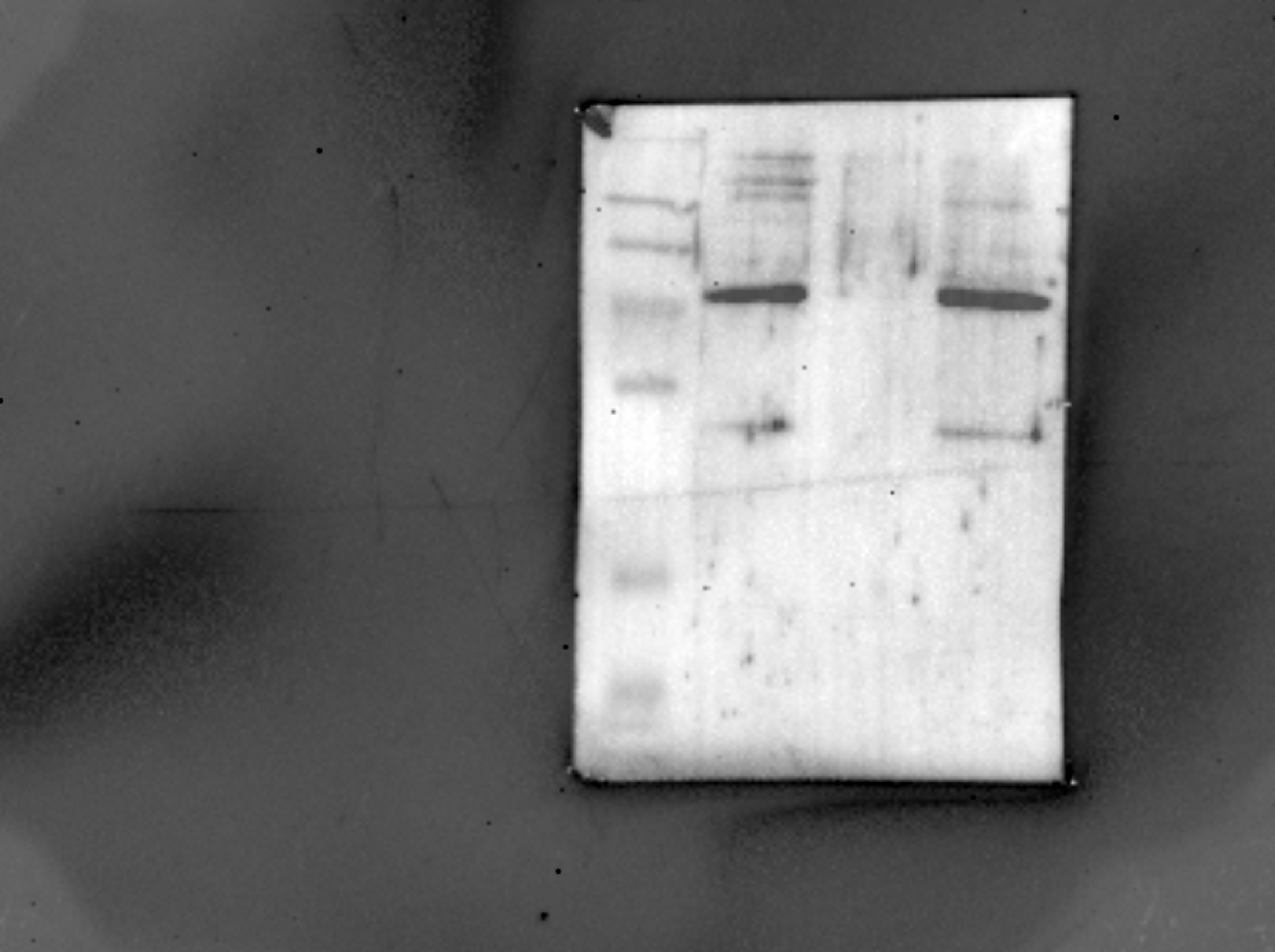

Supplement: Supplementary file 15 — Source Data [file 41467_2024_46972_MOESM15_ESM.zip › Espadas et al. 2024 Source Files/Espadas et al. 2024 Western Blots/TIF files of Western Blots/Supplementary Figure S4C synaptophysin synaptoneurosome.tif]

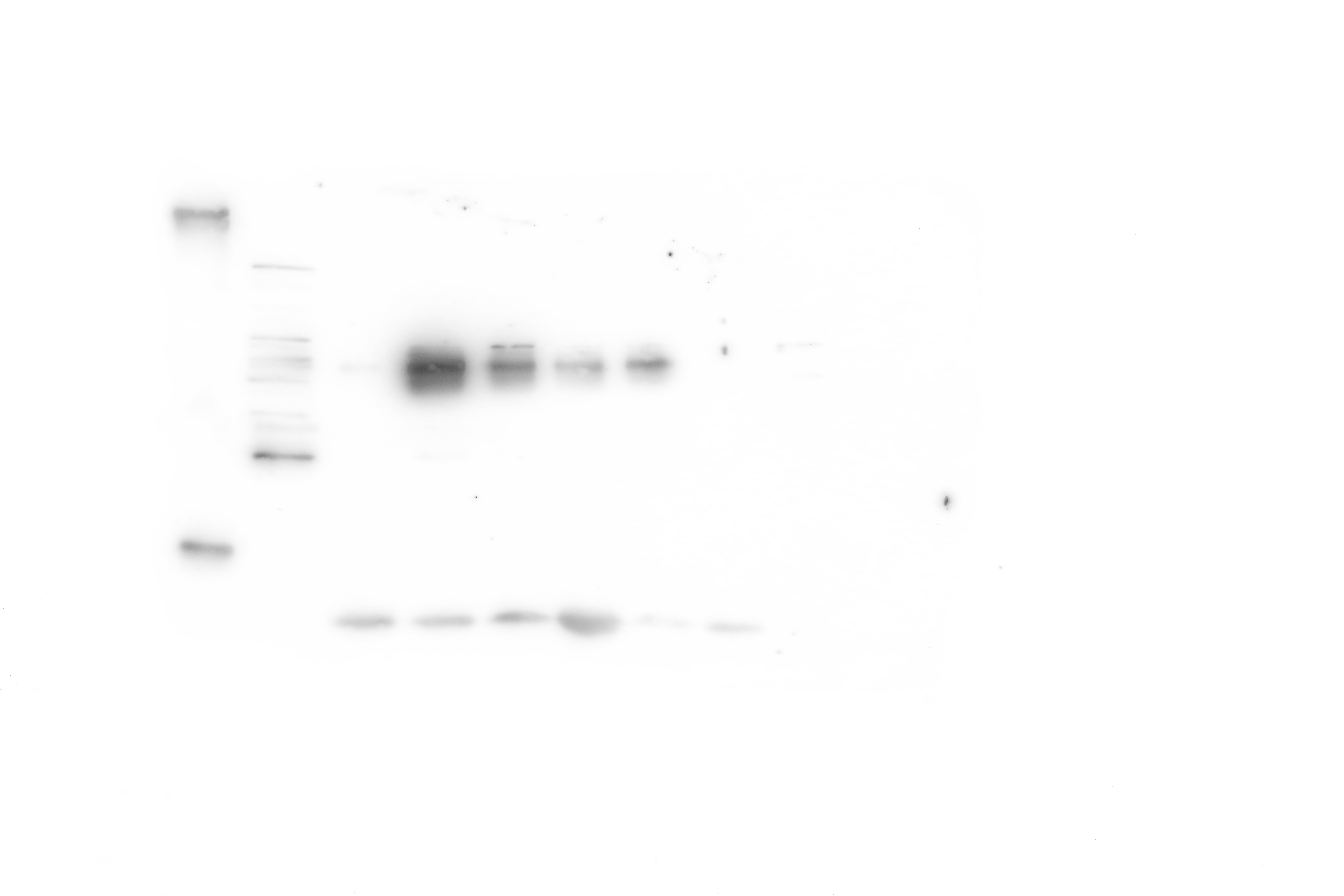

Supplement: Supplementary file 15 — Source Data [file 41467_2024_46972_MOESM15_ESM.zip › Espadas et al. 2024 Source Files/Espadas et al. 2024 Western Blots/TIF files of Western Blots/Figure 6J Vimentin pull down.jpg]

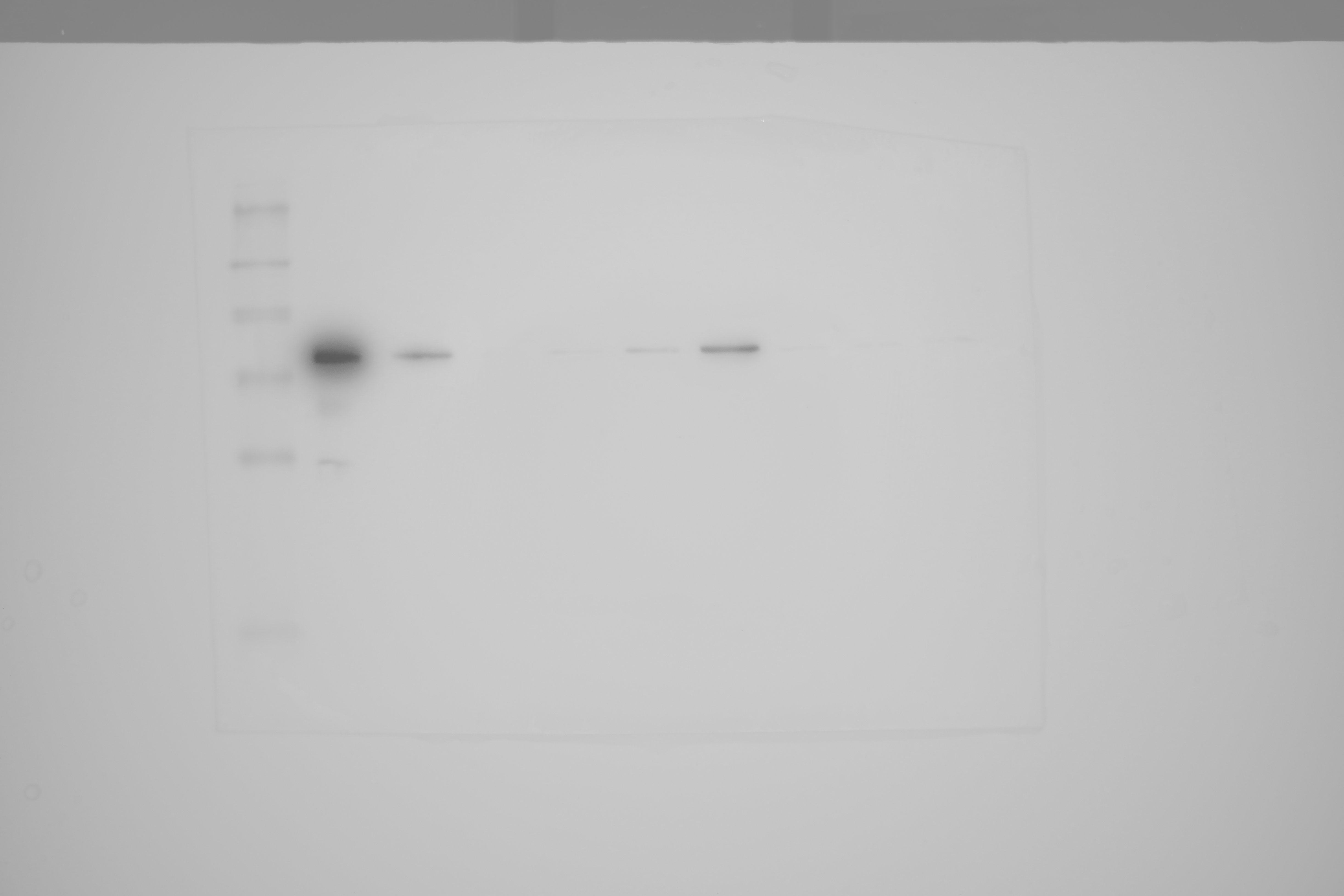

Supplement: Supplementary file 15 — Source Data [file 41467_2024_46972_MOESM15_ESM.zip › Espadas et al. 2024 Source Files/Espadas et al. 2024 Western Blots/TIF files of Western Blots/Figure 8D overlay protected camkIIa.jpg]

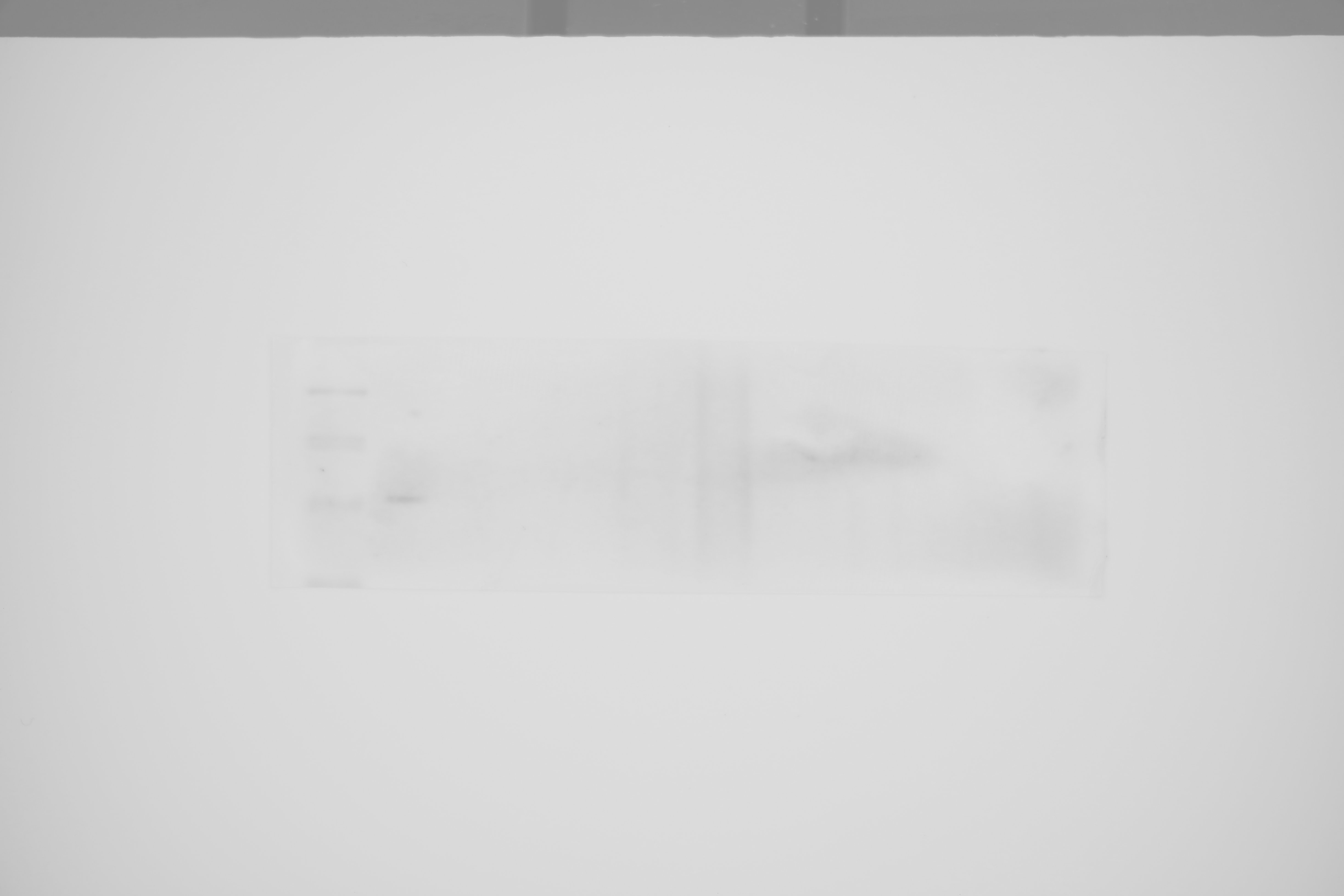

Supplement: Supplementary file 15 — Source Data [file 41467_2024_46972_MOESM15_ESM.zip › Espadas et al. 2024 Source Files/Espadas et al. 2024 Western Blots/TIF files of Western Blots/Figure 8D overlay Actin protected fragments.jpg]

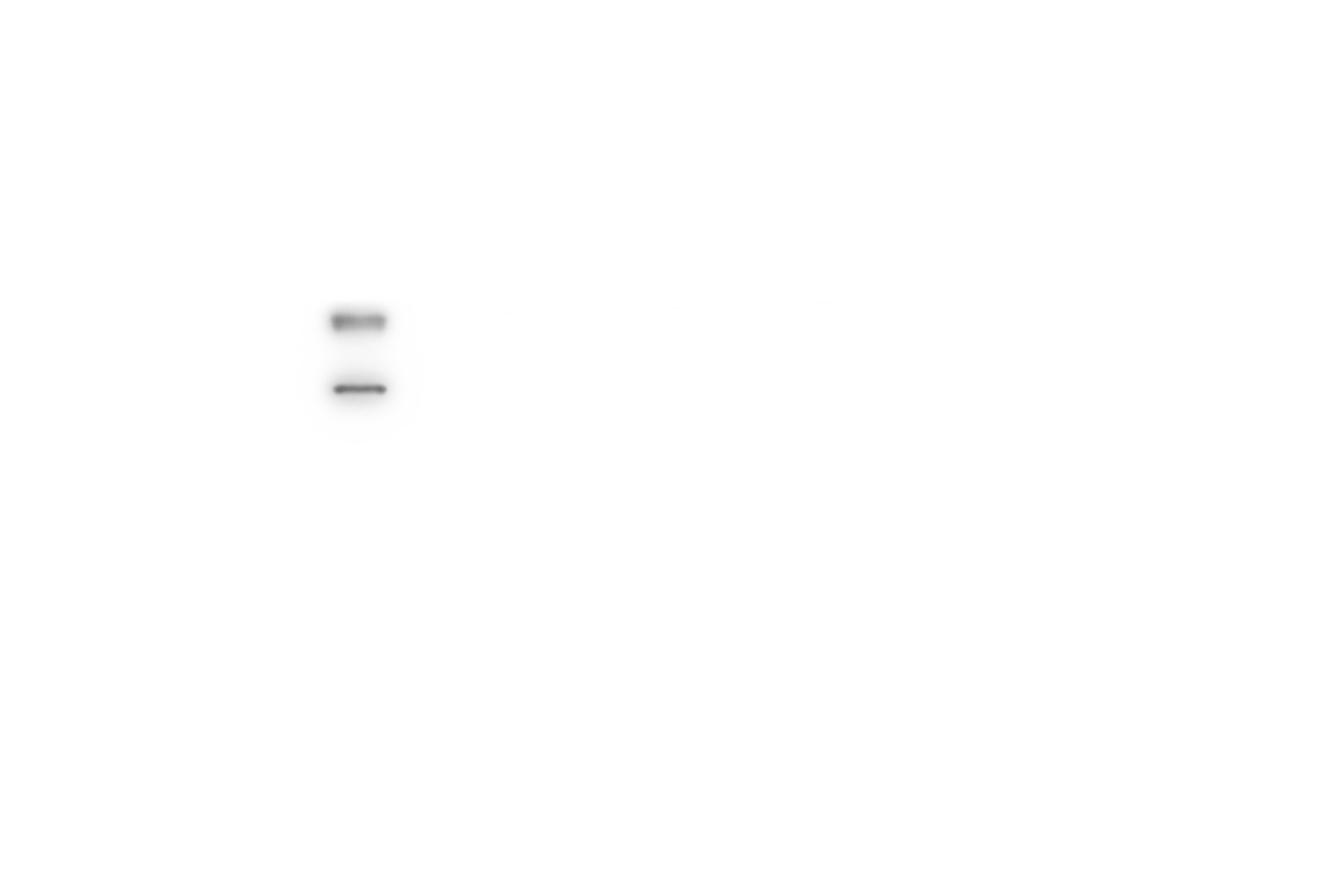

Supplement: Supplementary file 15 — Source Data [file 41467_2024_46972_MOESM15_ESM.zip › Espadas et al. 2024 Source Files/Espadas et al. 2024 Western Blots/TIF files of Western Blots/Figure 6F Gapdh (camkII) pull down.jpg]

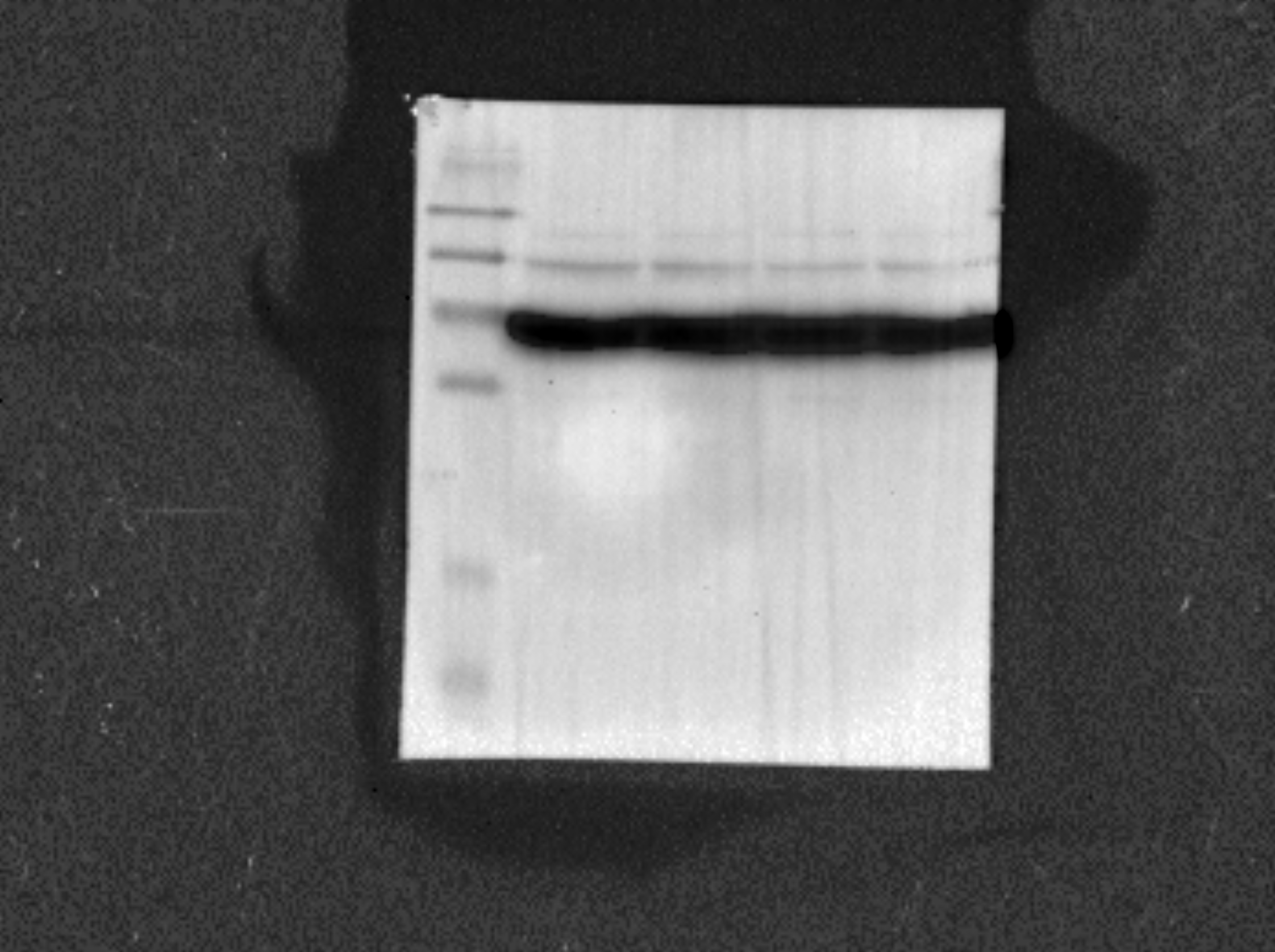

Supplement: Supplementary file 15 — Source Data [file 41467_2024_46972_MOESM15_ESM.zip › Espadas et al. 2024 Source Files/Espadas et al. 2024 Western Blots/TIF files of Western Blots/Figure 5L P70s6k.tif]

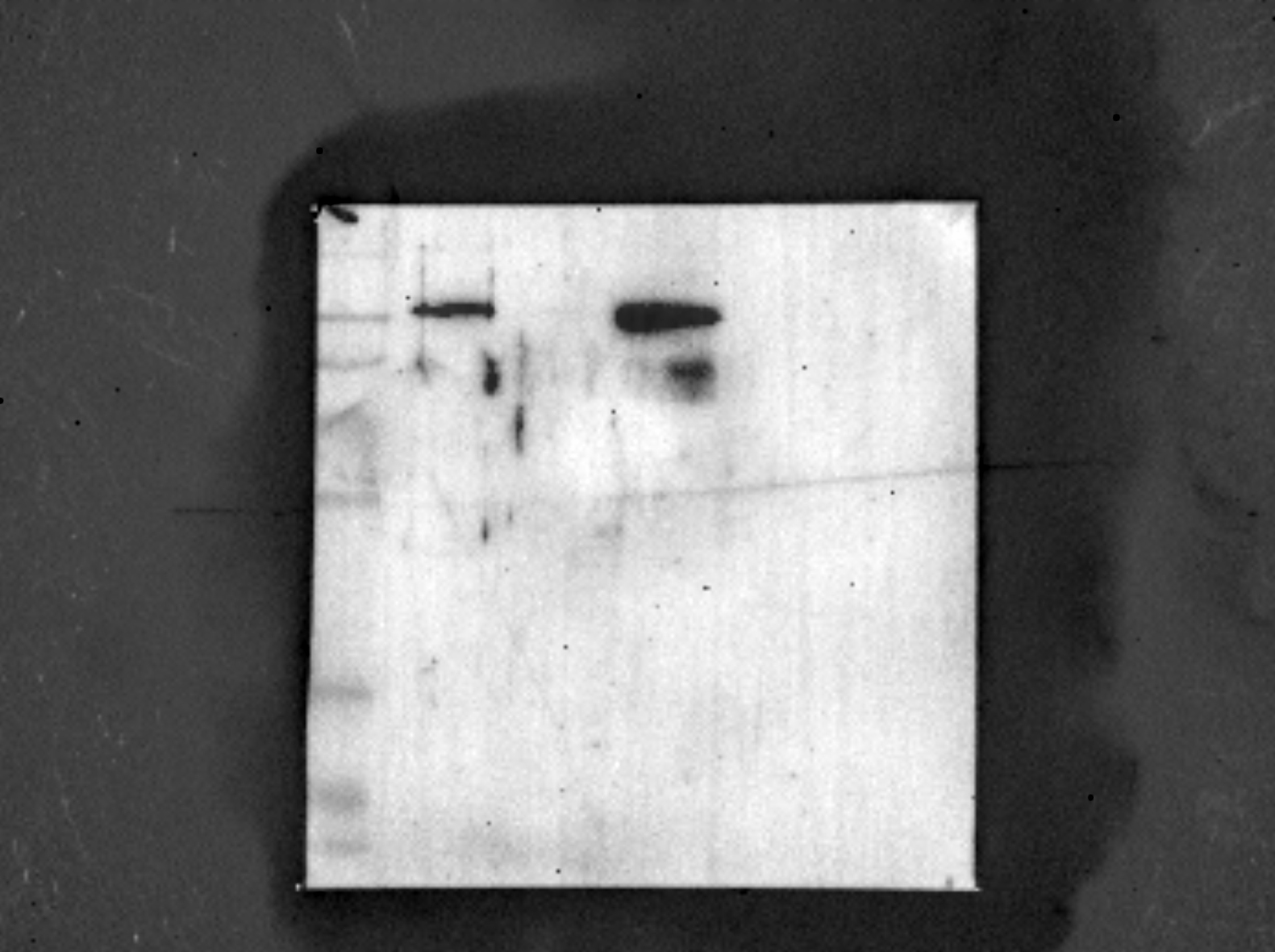

Supplement: Supplementary file 15 — Source Data [file 41467_2024_46972_MOESM15_ESM.zip › Espadas et al. 2024 Source Files/Espadas et al. 2024 Western Blots/TIF files of Western Blots/Supplementary Figure S4C glur2 synaptoneurosome.tif]

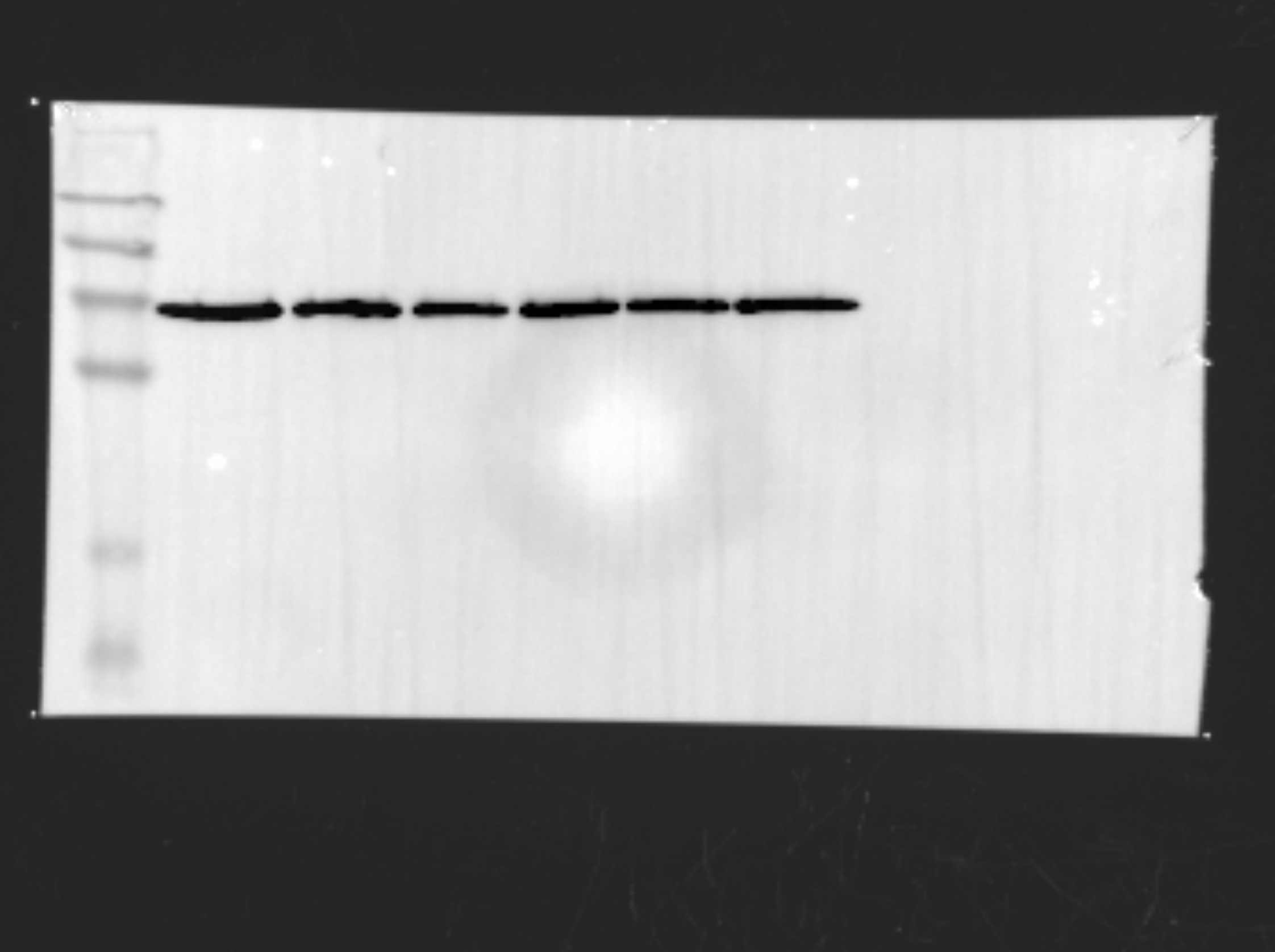

Supplement: Supplementary file 15 — Source Data [file 41467_2024_46972_MOESM15_ESM.zip › Espadas et al. 2024 Source Files/Espadas et al. 2024 Western Blots/TIF files of Western Blots/Figure 6H pcam-cam beta actin.tif]

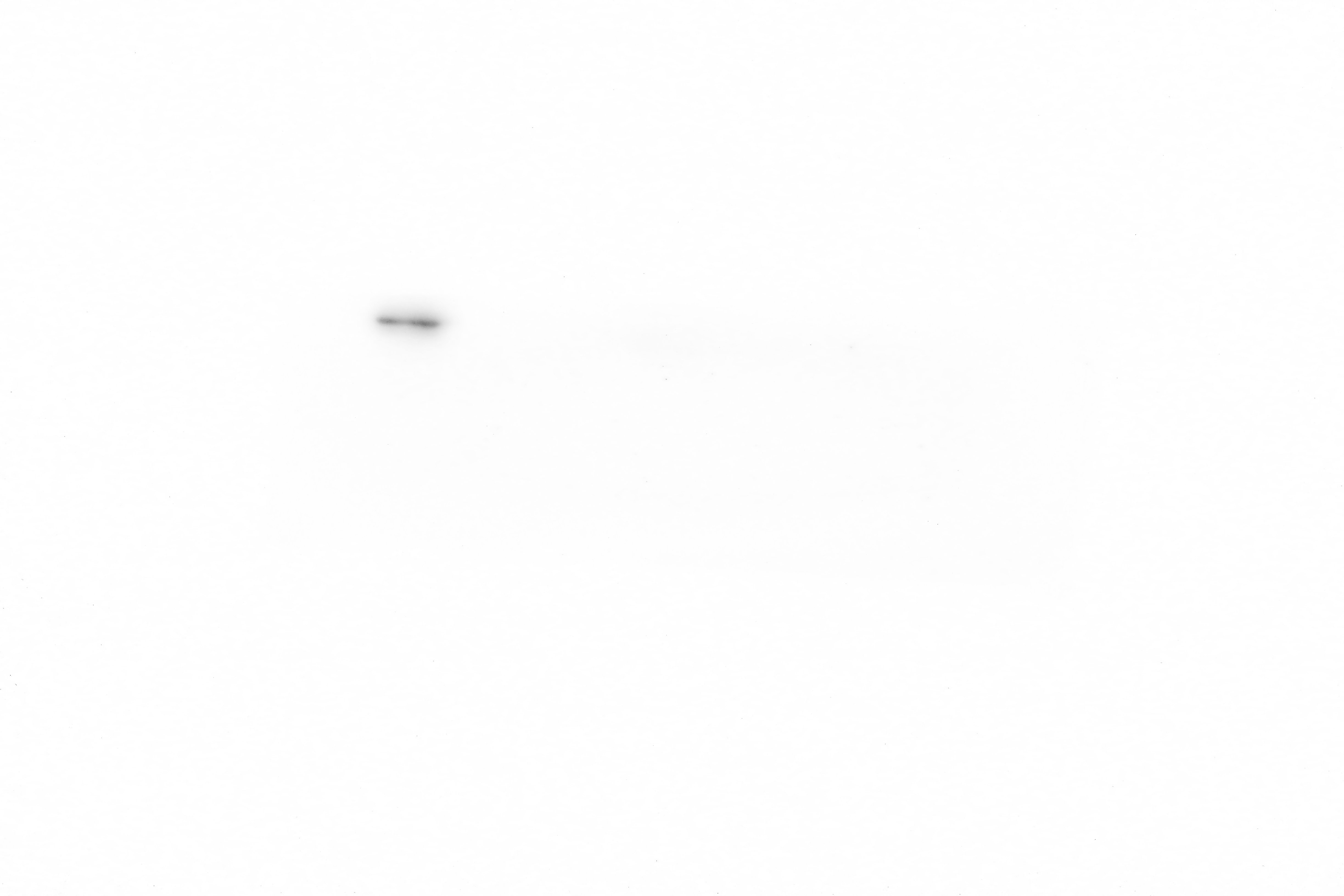

Supplement: Supplementary file 15 — Source Data [file 41467_2024_46972_MOESM15_ESM.zip › Espadas et al. 2024 Source Files/Espadas et al. 2024 Western Blots/TIF files of Western Blots/Figure 6J Gapdh (vimentin) pull down.jpg]

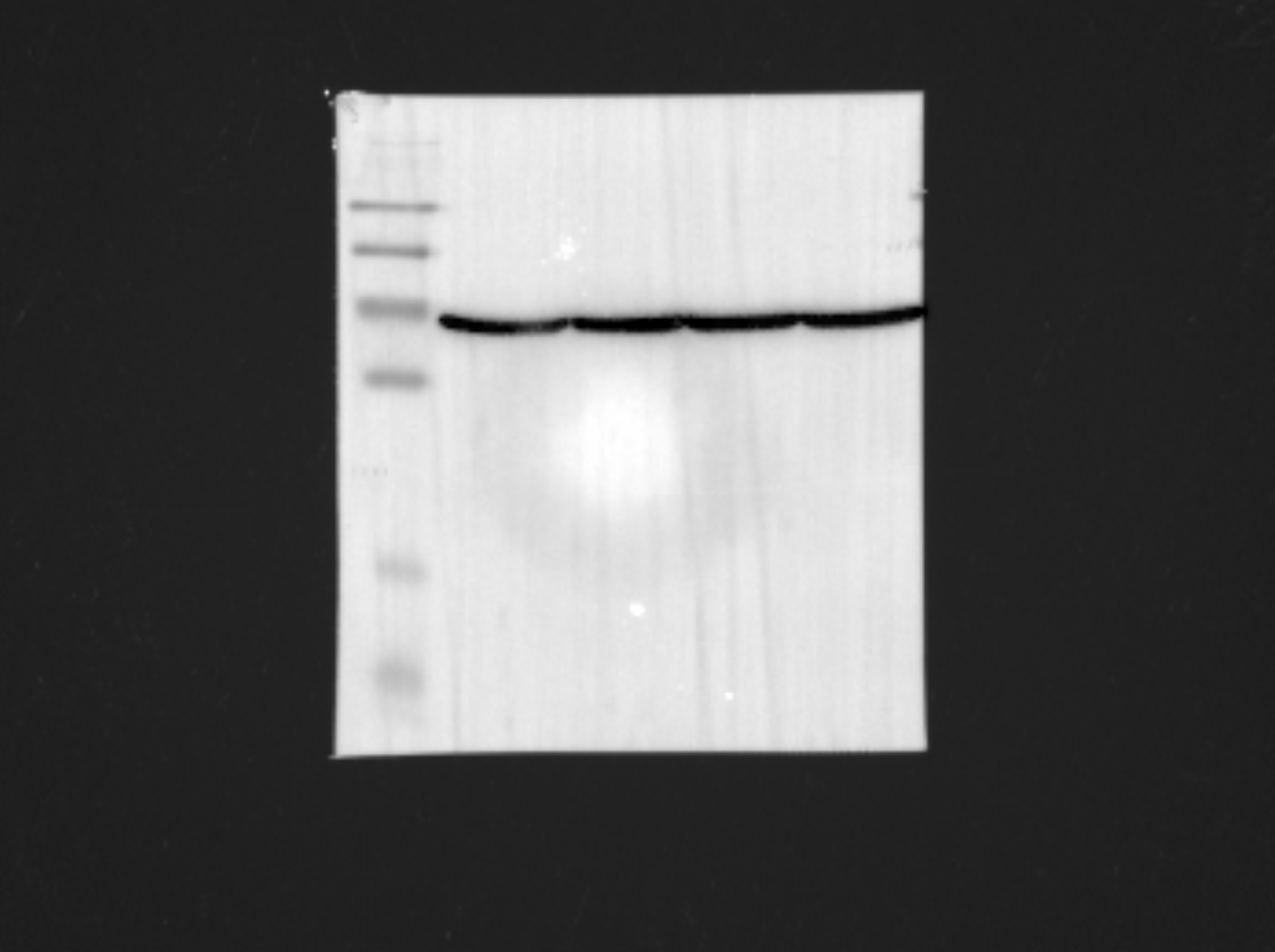

Supplement: Supplementary file 15 — Source Data [file 41467_2024_46972_MOESM15_ESM.zip › Espadas et al. 2024 Source Files/Espadas et al. 2024 Western Blots/TIF files of Western Blots/Figure 5L beta actin.tif]

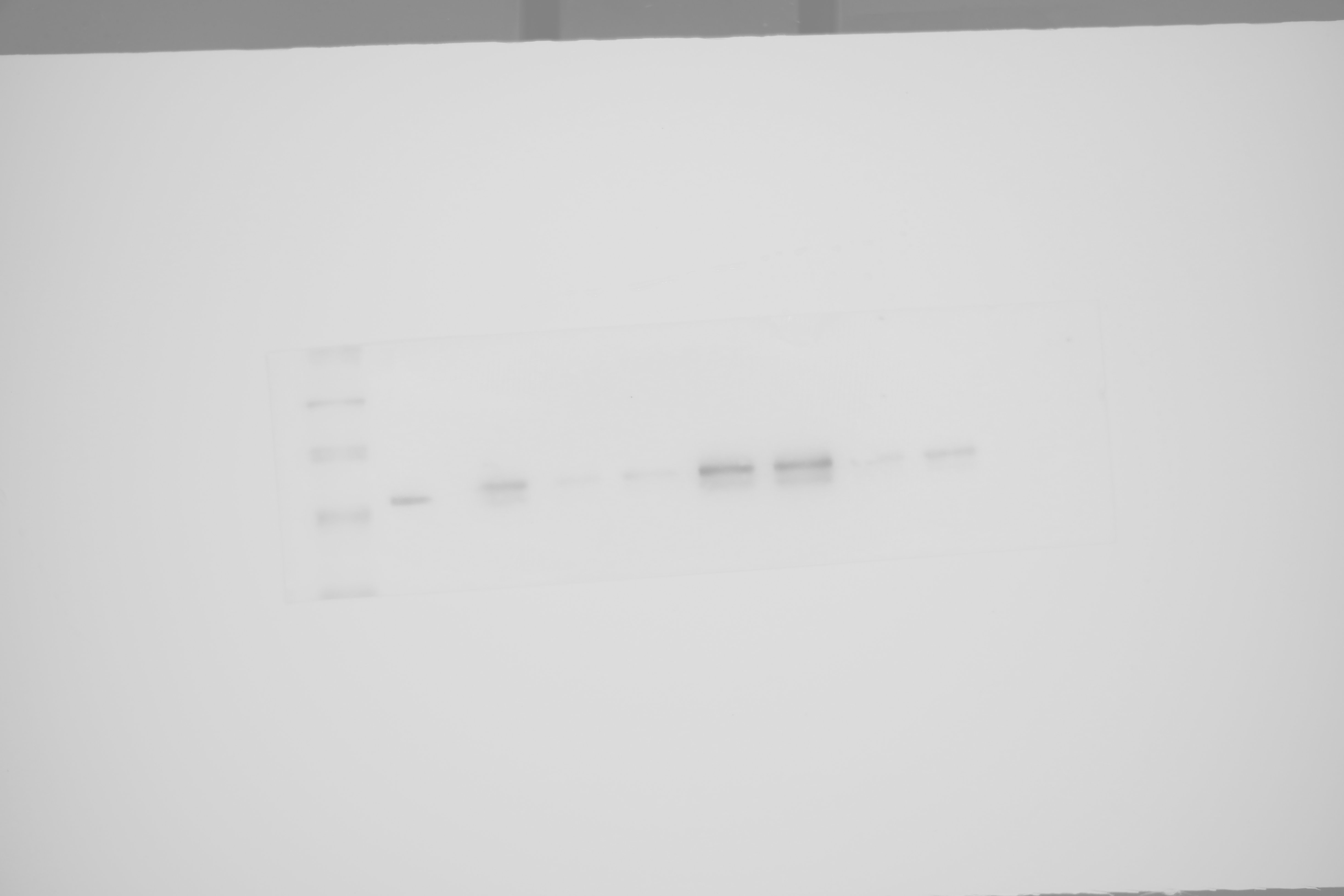

Supplement: Supplementary file 15 — Source Data [file 41467_2024_46972_MOESM15_ESM.zip › Espadas et al. 2024 Source Files/Espadas et al. 2024 Western Blots/TIF files of Western Blots/Figure 8D overlay Vimentin protected fragments.jpg]

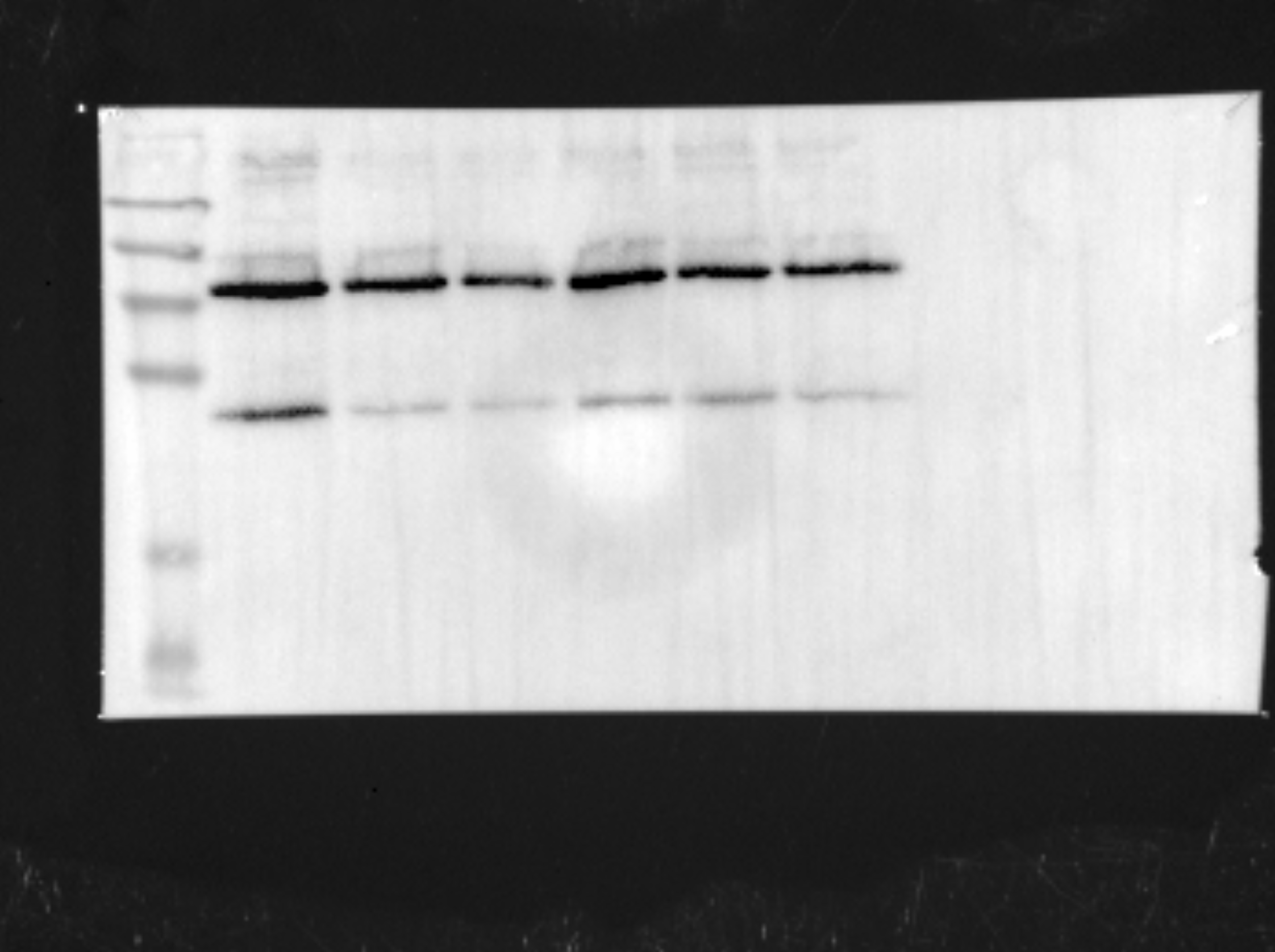

Supplement: Supplementary file 15 — Source Data [file 41467_2024_46972_MOESM15_ESM.zip › Espadas et al. 2024 Source Files/Espadas et al. 2024 Western Blots/TIF files of Western Blots/Figure 6H Camk2.tif]

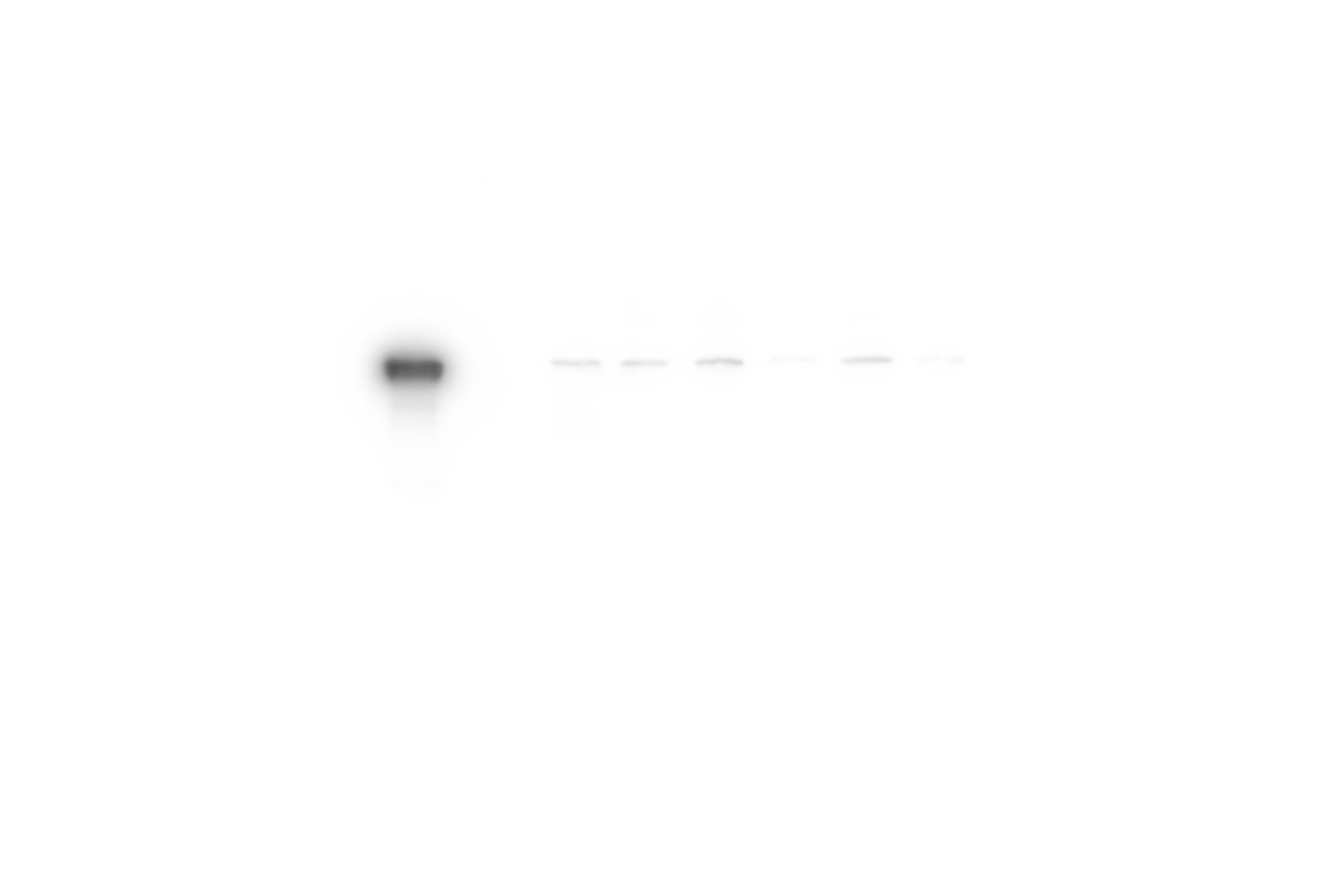

Supplement: Supplementary file 15 — Source Data [file 41467_2024_46972_MOESM15_ESM.zip › Espadas et al. 2024 Source Files/Espadas et al. 2024 Western Blots/TIF files of Western Blots/Figure 6F CamkII pull down.jpg]

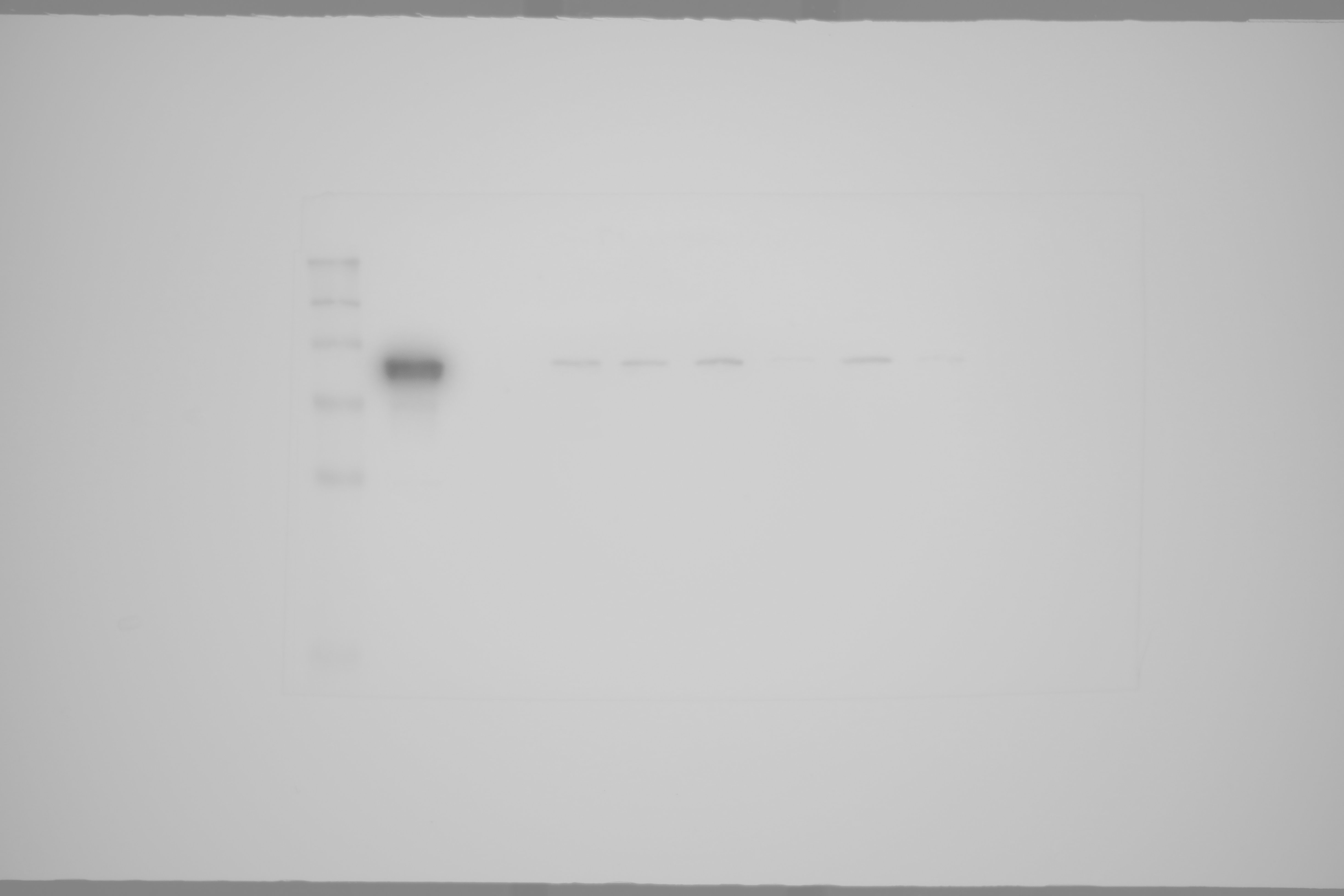

Supplement: Supplementary file 15 — Source Data [file 41467_2024_46972_MOESM15_ESM.zip › Espadas et al. 2024 Source Files/Espadas et al. 2024 Western Blots/TIF files of Western Blots/Figure 6F overlay CaMKII pull down.jpg]

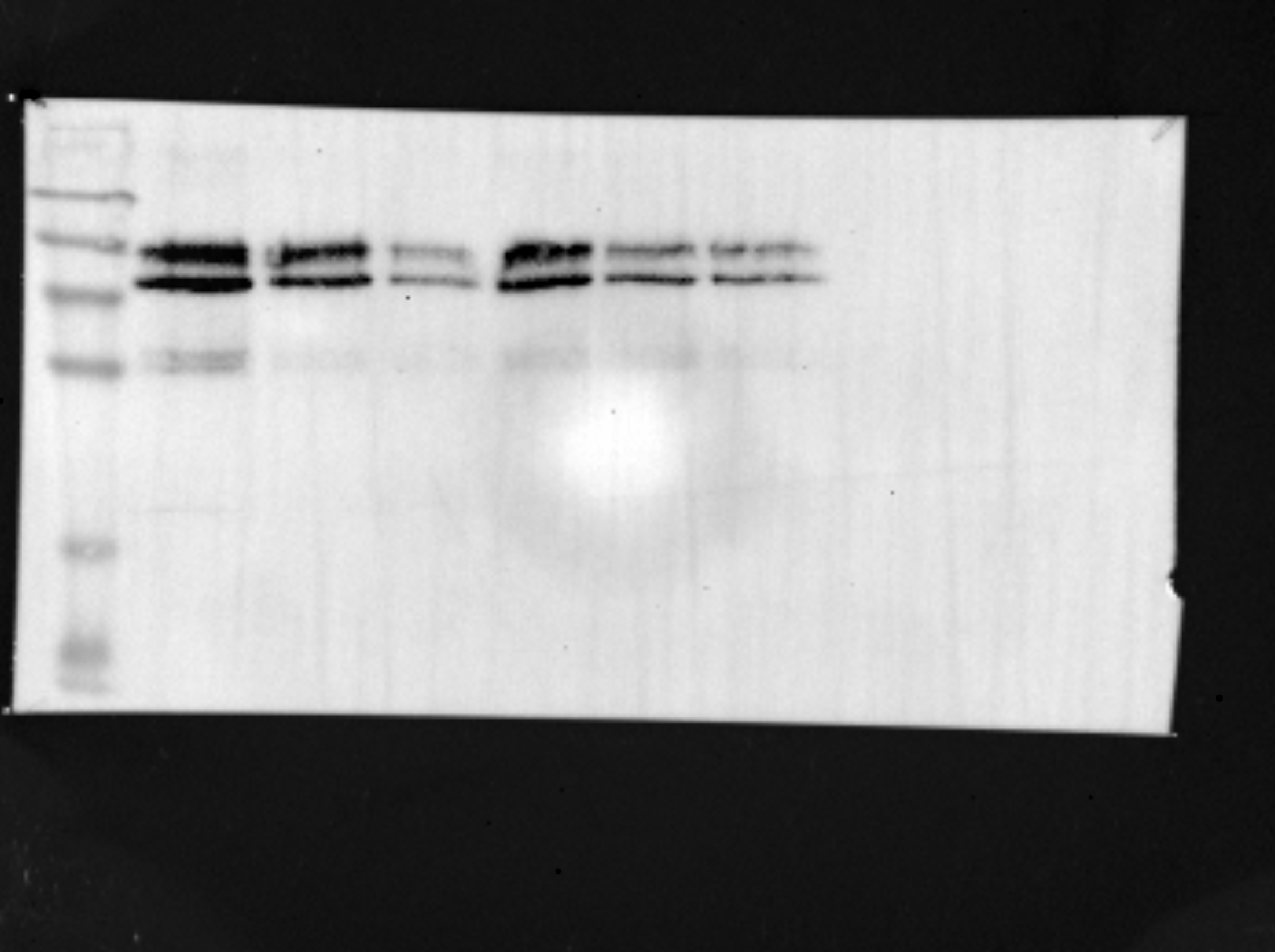

Supplement: Supplementary file 15 — Source Data [file 41467_2024_46972_MOESM15_ESM.zip › Espadas et al. 2024 Source Files/Espadas et al. 2024 Western Blots/TIF files of Western Blots/Figure 6H Pcamk2.tif]
